# CCL5 Orchestrates Paradoxical Immune Landscapes in NSCLC: Simultaneous Recruitment of Effector and Suppressor Cells Shapes Immunotherapy Resistance

**DOI:** 10.3390/cancers18081271

**Published:** 2026-04-16

**Authors:** Shuzhan Li, Jiali Zhang, Yang Wang, Zhenzhen Hui, Xinwei Zhang, Li Zhou, Xiubao Ren

**Affiliations:** 1 Tianjin Medical University Cancer Institute and Hospital, National Clinical Research Center for Cancer, Tianjin 300060, China; lishuzhan@tjmuch.com (S.L.);; 2 Tianjin’s Clinical Research Center for Cancer, Tianjin 300060, China; 3 Key Laboratory of Cancer Immunology and Biotherapy, Tianjin 300060, China; 4 Department of Biotherapy, Tianjin Medical University Cancer Institute and Hospital, Tianjin 300060, China; 5 Department of Immunology, Tianjin Medical University Cancer Institute and Hospital, Tianjin 300060, China

**Keywords:** CCL5, non-small cell lung cancer, immunotherapy, tumor microenvironment, biomarker

## Abstract

**Background:** The chemokine CCL5 exhibits a complex role in cancer immunotherapy, yet its dual immunomodulatory functions in non-small cell lung cancer (NSCLC) remain poorly understood. **Methods and Results:** Based on a newly analyzed clinical cohort of 33 advanced NSCLC patients receiving anti-PD-1 therapy combined with platinum-based chemotherapy, we found that elevated baseline peripheral blood CCL5 levels significantly predicted shorter overall survival (27.6 months vs. not reached, HR = 2.779, *p* = 0.038) and a higher incidence of immune-related pneumonitis (*p* = 0.0072). These clinical observations were supported by the re-analysis of a previously published single-cell RNA sequencing (scRNA-seq) dataset (*n* = 8), which indicated that high CCL5 expression in peripheral blood T/NK cells was associated with a lower major pathological response (*p* = 0.029). To explore the underlying mechanisms, we conducted detailed analyses using a large, publicly available tumor scRNA-seq dataset (GSE243013, *n* = 234). These analyses revealed that high intratumoral CCL5 simultaneously promoted the recruitment of both immune effector cells (CD8^+^ T cells, NK cells) and immunosuppressive populations (Tregs, MDSCs). This paradoxical immune landscape correlated with elevated immune checkpoint expression and significantly higher TIDE scores (1.47 vs. 0.83, *p* < 0.001). CellChat and SCENIC network analyses identified intensified T cell–myeloid communication and key transcription factors (e.g., FOXP3, EOMES) mediating this dichotomy. **Conclusions:** This hypothesis-generating study raises the possibility that CCL5 orchestrates paradoxical immune responses and may serve as a biomarker in NSCLC. Further validation in larger prospective, independent cohorts is required.

## Simple Summary

Non-small cell lung cancer (NSCLC) is a deadly cancer worldwide. Combining immunotherapy with chemotherapy has helped many patients. However, challenges such as resistance and side effects such as lung inflammation have yet to be overcome. This study investigated the role of CCL5, an immune-signaling protein, in advanced NSCLC patients. By newly analyzing a clinical cohort of 33 patients, we found that higher baseline CCL5 levels in the blood predicted shorter survival and a higher risk of lung inflammation. To understand these results, we re-analyzed previously published and publicly available single-cell datasets. These analyses suggest that high CCL5 attracts cancer-fighting and immune-suppressing cells simultaneously, creating a paradoxical environment that helps the cancer escape treatment. These findings raise the possibility that CCL5 could serve as a biomarker and therapeutic target, although further validation in larger independent studies is required.

## 1. Introduction

Non-small cell lung cancer (NSCLC), including lung squamous carcinoma (LUSC) and lung adenocarcinoma (LUAD), accounts for over 80% of all lung cancer cases [1]. With an estimated 2.2 million new diagnoses and 1.8 million deaths annually as of 2020, NSCLC poses a significant public health challenge, particularly in its advanced stages, for which systemic therapies are the cornerstone of management [2]. The advent of immune checkpoint inhibitors (ICIs), particularly programmed death-1 (PD-1) and programmed death-ligand 1 (PD-L1) antibodies combined with platinum-based chemotherapy, has revolutionized the treatment landscape for patients with advanced and metastatic NSCLC [3]. These treatment regimens have shown greater effectiveness than chemotherapy alone, with landmark trials such as KEYNOTE-189 and CheckMate-9LA reporting improved overall survival (OS) rates of up to 50% at 2 years in PD-L1-positive patients [4,5]. However, despite these therapeutic advances, drug resistance persists as an inevitable reality, with only 15–30% of patients achieving durable clinical benefits, while others develop primary or acquired resistance [6,7]. Factors contributing to this variability include tumor mutational burden (TMB), PD-L1 expression, and microsatellite instability, which are limited by accuracy and differences between assays [8]. Moreover, heterogeneous responses to immunotherapy are complicated by immune-related adverse events (irAEs), including potentially life-threatening conditions such as immune-related pneumonitis, which occurs in 3–6% of patients and significantly impacts treatment outcomes [9]. The limitations of these established biomarkers underscore the need for novel predictive markers for optimal therapeutic strategies and the prediction of irAEs.

It is now understood that the tumor microenvironment (TME) is crucial in the development and management of human cancer [10]. Tumor cells influence the TME through inflammatory cytokines and chemokines, which restrict the immune response, thereby promoting tumor progression and impeding cancer therapy [11]. Chemokine ligand 5 (CCL5), also known as regulated upon activation, normal T cell expressed and secreted (RANTES), is a member of the C-C motif chemokine family that plays a pivotal role in orchestrating immune responses by recruiting and activating various immune cells, including T and natural killer (NK) cells [12,13]. CCL5 binds to receptors such as CCR1, CCR3, and CCR5, facilitating chemotaxis and modulating inflammatory processes [14]. CCL5 exhibits a multifaceted role in cancer and inflammatory diseases. For example, CCL5 promotes anti-tumor immunity by attracting cytotoxic T lymphocytes and NK cells to the TME, thereby enhancing immune surveillance and potentially sensitizing tumors to ICIs [15,16]. It may also promote pro-tumorigenic effects by attracting immunosuppressive cells, such as regulatory T cells (Tregs) and myeloid-derived suppressor cells (MDSCs), resulting in immune evasion and tumor progression [17,18,19]. Circulating CCL5 reflects systemic inflammation or immune function [20], whereas its expression within tumors may indicate local TME dynamics [16,17]. In pan-cancer studies, CCL5 was positively correlated with prolonged OS in urothelial carcinoma (UC) and esophageal squamous cell carcinoma (ESCC) [21]. In NSCLC, several studies have identified a correlation between poor prognosis and elevated CCL5 [22,23,24,25]. High concentrations of CCL5 in the peripheral blood may indicate a higher incidence of irAEs in NSCLC patients undergoing nivolumab monotherapy [26]. However, CCL5 recruits more CD8^+^ T cells and improves the outcome of PD-1/PD-L1 blockade combined with anlotinib in NSCLC [27]. This complexity is further emphasized by the findings that although CCL5 can attract immune effector cells that fight tumors, it might also encourage the entry of immunosuppressive cell groups, resulting in immune evasion and resistance to treatment.

Despite the growing interest in chemokines as immunomodulators, the multifaceted role of CCL5 in NSCLC immunotherapy remains underexplored. In this study, we conducted a comprehensive multi-omics investigation. First, we analyzed a newly collected clinical cohort (*n* = 33) of advanced NSCLC patients receiving anti-PD-1 monoclonal antibodies combined with platinum-based chemotherapy, revealing a relationship between overall survival and the baseline concentration of peripheral blood CCL5. Second, to investigate the underlying cellular mechanisms, we re-analyzed a previously published single-cell RNA sequencing (scRNA-seq) dataset (*n* = 8) from our group and extensively mined a large, publicly available tumor scRNA-seq dataset (GSE243013, *n* = 234). These combined analyses demonstrated a correlation between CCL5 expression and the simultaneous infiltration of immune-activating and immune-suppressive cells, elevated immune checkpoint expression, and potential drug resistance. CellChat analysis indicated that CCL5 influences the TME through T cell–myeloid cell communication via immune-activating and immune-suppressive signals. Ultimately, this hypothesis-generating study raises the possibility that circulating CCL5 may serve as a biomarker associated with clinical outcomes and toxicity in NSCLC. Supported by these comprehensive single-cell analyses, we also propose a potential mechanism underlying the paradoxical immune modulatory functions of CCL5, providing insights for future combination immunotherapy strategies.

## 2. Materials and Methods

### 2.1. Patient Cohorts and Clinical Data Collection

Clinical cohort (*n* = 33): This study included a total of 33 patients with advanced NSCLC who received PD-1 inhibitors (sintilimab, Innovent Biologics, Suzhou, China) combined with platinum-based chemotherapy between 17 May 2019, and 15 January 2021, at Tianjin Medical University Cancer Institute and Hospital. This cohort represents a retrospective analysis of clinical and baseline plasma cytokine data from our previously published Phase Ib clinical trial [28]. The primary inclusion criteria for the original trial were as follows: (1) histologically confirmed Stage III or IV NSCLC; (2) no prior systemic immunotherapy; and (3) adequate organ function. Exclusion criteria included active autoimmune diseases or symptomatic brain metastases. Immune-related pneumonitis was graded according to the CTCAE, version 5.0.

scRNA-seq datasets (*n* = 8 and *n* = 234): To investigate the cellular origin and mechanistic role of CCL5, we utilized two previously generated single-cell transcriptomic datasets. First, we re-analyzed PBMC data from 8 advanced NSCLC patients who received neoadjuvant chemoimmunotherapy, which was previously published by our group [29]. Second, a publicly available large-scale scRNA-seq dataset (GSE243013) composed of tumor samples from 234 advanced NSCLC patients was downloaded from the Gene Expression Omnibus (GEO). For both datasets, we utilized the pre-processed, normalized gene expression matrices and the original cell type annotations provided by the respective authors. Consequently, no additional primary normalization or cell clustering algorithms were applied in this study; all downstream bioinformatics analyses were conducted based on these pre-processed matrices.

### 2.2. Peripheral Blood CCL5 Measurement

Plasma samples and hematological parameters were collected within the week prior to treatment. To isolate plasma, EDTA-anticoagulated whole blood samples were centrifuged (1000× *g*, 15 min). CCL5 levels were measured in plasma samples using the 45-ProcartaPlex Human Cytokine/Chemokine/Growth Factor Panel (Affymetrix, Inc., Santa Clara, CA, USA) and the 14-ProcartaPlex Human Immuno-Oncology Checkpoint Panel (Affymetrix, Inc.). After log2 transformation of the concentration values, a signature score was calculated by averaging the included cytokines.

### 2.3. Public Database Analysis

#### 2.3.1. Lung Cancer Explorer Analysis

CCL5 mRNA expression levels in NSCLC tumor tissues versus normal lung tissues were analyzed using the online Lung Cancer Explorer tool (http://lce.biohpc.swmed.edu/lungcancer). The analysis included datasets for LUAD and LUSC. Expression data were log2-transformed, and differential expression between tumor and normal tissues was assessed using the Wilcoxon rank-sum test.

#### 2.3.2. Kaplan–Meier Plotter Analysis

The prognostic significance of CCL5 expression in lung cancer was evaluated using the Kaplan–Meier Plotter database (http://www.kmplot.com). The analysis included 1411 NSCLC patients with OS and PFS data from publicly available microarray datasets. Patients were dichotomized into high and low CCL5 expression groups using the auto-selected best cutoff function. Subgroup analyses were performed separately for adenocarcinoma and squamous cell carcinoma.

#### 2.3.3. Circulating Tumor Cell Analysis

CCL5 expression measurement of circulating tumor cells (CTCs) in LUSC was performed using an online website (http://www.origin-gene.cn/database/ctcRbase/index.html). GSE74639 was analyzed using the Wilcoxon rank-sum test. A *p*-value < 0.05 was considered statistically significant.

### 2.4. Tumor Microenvironment Analysis

#### 2.4.1. Single-Sample Gene Set Enrichment Analysis

The infiltration levels of various immune cell subsets in the tumor microenvironment were quantitatively evaluated using single-sample gene set enrichment analysis (ssGSEA) implemented in the GSVA R package (version 1.52.3). Gene expression data from The Cancer Genome Atlas (TCGA)-LUAD and TCGA-LUSC were downloaded and processed using DESeq2 for normalization. Gene signatures for 28 immune cell types were obtained from previous studies [30,31]. ssGSEA scores were calculated for each immune cell type in each sample, representing the relative enrichment of each cell type. Samples were stratified into CCL5-high and CCL5-low groups based on median expression, and immune infiltration scores were compared using the Wilcoxon rank-sum test.

#### 2.4.2. Multi-Algorithm Immune Cell Infiltration Analysis

Seven independent algorithms were employed to enhance the robustness of the immune infiltration analysis: XCELL, TIMER, QUANTISEQ, MCPcounter, EPIC, CIBERSORT, and CIBERSORT-ABS. These analyses were performed using the TIMER2.0 web platform (http://timer.cistrome.org/) and the immunedeconv R package (version 2.1.3).

#### 2.4.3. Estimate Score Calculation

The Estimation of Stromal and Immune cells in Tumor tissues using Expression data (ESTIMATE) algorithm was employed to calculate three scores for each sample from TCGA-LUAD and TCGA-LUSC. ESTIMATE scores were calculated using the estimate R package (version 1.0.13) with normalized gene expression data as input. Scores were compared between CCL5-high and CCL5-low groups using the Wilcoxon rank-sum test.

#### 2.4.4. Immune Checkpoint Expression Analysis

Expression levels of immune checkpoint molecules, including PD-1 (PDCD1), PD-L1 (CD274), CTLA-4, LAG3, and TIM-3 (HAVCR2), were extracted from TCGA-LUAD and TCGA-LUSC. The expression levels of these checkpoint molecules were compared between CCL5-high and CCL5-low groups using the Wilcoxon rank-sum test.

#### 2.4.5. Tumor Immune Dysfunction and Exclusion Score Analysis

Tumor immune dysfunction and exclusion (TIDE) scores were calculated to predict immune evasion potential and immunotherapy response using the TIDE web platform (http://tide.dfci.harvard.edu). Normalized gene expression data and sample annotations were uploaded to the platform. Higher TIDE scores indicate greater likelihood of immune evasion and poor response to immunotherapy. TIDE scores were compared between CCL5-high and CCL5-low groups using the Wilcoxon rank-sum test.

#### 2.4.6. Drug Sensitivity Analysis

Drug sensitivity data were obtained from the Genomics of Drug Sensitivity in Cancer (GDSC) database (http://www.cancerrxgene.org). The association between CCL5 expression and drug sensitivity was analyzed for targeted therapies and chemotherapeutic agents commonly used in NSCLC treatment. Gene expression data from GDSC cancer cell lines were correlated with drug sensitivity measurements (IC50 values) using the Wilcoxon rank-sum test. A negative correlation indicates that higher CCL5 expression is associated with increased drug resistance (higher IC50 values). Analysis was performed using the oncoPredict R package (version 1.2).

### 2.5. CCL5 Expression Analysis in Single-Cell Data

For the PBMC cohort, patients were stratified based on their treatment responses. Pseudobulk analysis was performed by aggregating gene expression across all T/NK cells within each patient to obtain patient-level CCL5 expression values. Pseudobulk CCL5 expression was then compared between major pathological response (MPR) and non-MPR groups. For the publicly available tumor tissue dataset (GSE243013), samples were stratified into CCL5-high and CCL5-low groups based on the median CCL5 expression across all cells.

### 2.6. T Cell Functional Scoring Using UCell

T cell functional states were quantified using the UCell method (version 2.8.0), which calculates enrichment scores for gene signatures at the single-cell level without requiring normalization or differential expression analysis. Gene signatures for the following T cell states are provided in Supplementary Materials (Data S2). UCell scores were calculated for each T cell in the publicly available dataset GSE243013. Functional scores were compared between CCL5-high and CCL5-low groups using the Wilcoxon rank-sum test. Results were visualized using box plots showing the distribution of each functional score across CCL5 expression groups.

### 2.7. Differential Expression Analysis and Gene Set Enrichment Analysis

To identify genes and pathways associated with CCL5 expression, differential expression analysis was performed comparing CCL5-high versus CCL5-low groups within the publicly available GSE243013 dataset. Differentially expressed genes (DEGs) were identified using the FindMarkers function in Seurat (Wilcoxon rank-sum test). The thresholds for defining statistical significance were strictly set at an adjusted *p*-value < 0.05 (False Discovery Rate) and an absolute log2 fold-change > 0.25. Gene Set Enrichment Analysis (GSEA) was performed using the DAVID online tool (https://david.ncifcrf.gov/). The list of DEGs was tested for enrichment in Gene Ontology (GO) terms: Biological Process, Cellular Component, Molecular Function; Kyoto Encyclopedia of Genes and Genomes (KEGG) pathways; Reactome pathways; Hallmark gene sets from the Molecular Signatures Database (MSigDB). Significantly enriched pathways (adjusted *p*-value < 0.05, or FDR < 0.25 for GSEA) were identified, with particular focus on immune-related pathways including T cell activation, cytokine signaling, and immune regulation. Results were visualized using bar plots enrichment maps showing the most significantly enriched pathways and their associated genes.

### 2.8. Cell–Cell Communication Analysis Using CellChat

Intercellular communication networks were analyzed using CellChat (version 1.6.1) to investigate interactions between different cell types in the tumor microenvironment. Normalized gene expression data from the publicly available dataset GSE243013 and the provided cell type annotations were input into CellChat. Specifically, the CellChatDB database for humans was used. Chord diagrams illustrate specific ligand–receptor interactions, while heatmaps show differential communication patterns between CCL5-high and CCL5-low groups.

### 2.9. Gene Regulatory Network Analysis Using SCENIC

A single-cell regulatory network inference and clustering (SCENIC) analysis was performed to identify transcription factors (TFs) and their downstream target genes (regulons) that are differentially active between CCL5-high and CCL5-low T cells in the tumor microenvironment. This analysis was conducted using the pySCENIC Python package (version 0.12.1). Co-expression modules were identified using the GRNBoost2 algorithm, which infers regulatory relationships between transcription factors and potential target genes based on their co-expression patterns across cells. To refine the gene regulatory networks, RcisTarget was used to identify enriched transcription factor binding motifs in the promoter regions (and enhancers) of co-expressed target genes. Motif databases for humans (hg38 genome assembly) were utilized. Only regulons (TF plus target genes) with significant motif enrichment (Normalized Enrichment Score, NES > 3.0) were retained for downstream analysis. The activity of each validated regulon in individual cells was quantified using AUCell (Area Under the Curve). AUCell calculates an enrichment score representing how many genes from a regulon’s gene signature are expressed above a certain threshold in each cell, independent of other cells in the dataset. Network diagrams illustrate transcription factors and their top target genes.

### 2.10. Statistical Analysis

Statistical analyses were performed using R and R Studio (version 2025.05.1+513) and GraphPad Prism (version 10.1.1, GraphPad Software, San Diego, CA, USA). Continuous variables were expressed as the median with a range or the mean ± standard deviation (SD), as appropriate, and compared using Student’s *t*-test (for normally distributed data) or the Wilcoxon rank-sum test (Mann–Whitney U test for non-normally distributed data).

### 2.11. Data Availability

Single-cell RNA sequencing data from PBMC samples are available from the corresponding author on reasonable request as another study is being conducted. The publicly available tumor tissue scRNA-seq data used in this study can be accessed from GEO under accession number GSE243013.

### 2.12. Code Availability

This study did not generate any unique code. All code or software tools used in this study follow the official guidelines and are freely or commercially available.

## 3. Results

### 3.1. Predictive Value for Prognosis and irAEs of Initial CCL5 Concentration in Peripheral Blood of Advanced NSCLC

Our previous study cohort included 33 patients with advanced NSCLC who received PD-1 inhibitors combined with platinum-based chemotherapy [28]. The median age was 62.5 years (range: 40–73), and 29 (87.9%) patients were male. Histological examination revealed 14 (39.4%) adenocarcinomas and 19 (57.6%) squamous cell carcinomas. Most patients (27/33, 81.8%) presented with stage IV disease. The treatment regimens included sintilimab plus platinum-based chemotherapy.

The baseline peripheral blood CCL5 levels ranged from 7.08 to 250.51 pg/mL, with a median value of 150.83 pg/mL. The median value was set as the cutoff value. Based on this threshold, 16 patients (48.5%) were classified as CCL5-high and 17 patients (51.5%) as CCL5-low. No significant associations were observed between baseline CCL5 levels and patient demographics, tumor stage, or histological subtypes (all *p* > 0.05).

**Figure 1:**
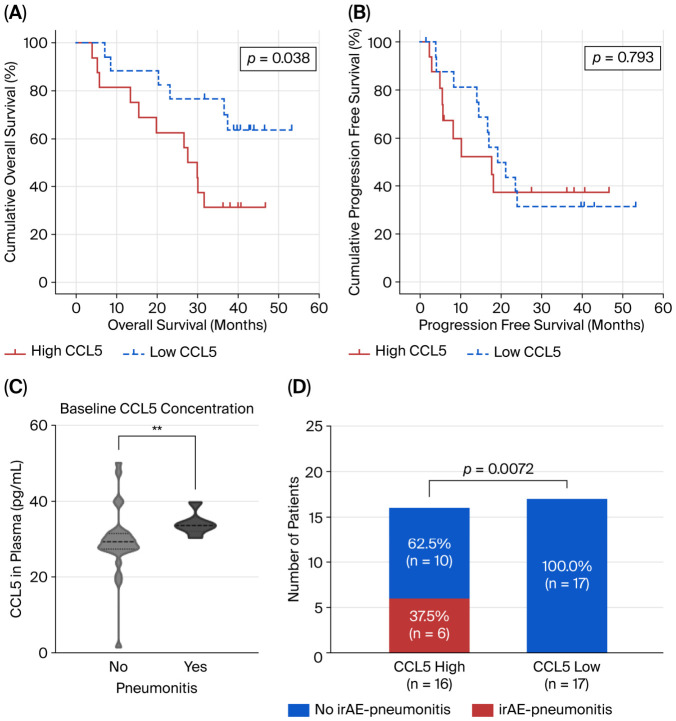
Baseline CCL5 levels in peripheral blood correlates with clinical outcomes and immunotherapy-related pneumonitis in NSCLC patients receiving chemoimmunotherapy. (**A**) Kaplan–Meier curves for overall survival (OS) stratified by baseline CCL5 levels. Patients with low CCL5 levels (blue line) demonstrated significantly longer OS compared with those with high CCL5 levels (red line). (**B**) Kaplan–Meier curves for progression-free survival (PFS) stratified by baseline CCL5 levels. No significant difference in PFS was observed between low CCL5 (blue line) and high CCL5 (red line) groups. (**C**) Violin plots showing baseline CCL5 plasma concentrations in patients with and without immune-related adverse event pneumonitis. Patients who developed irAE-pneumonitis had significantly higher baseline CCL5 levels compared with those without pneumonitis (** *p* < 0.01, Mann–Whitney U test). (**D**). Incidence of irAE-pneumonitis stratified by CCL5 levels. Patients with high baseline CCL5 showed a significantly higher rate of irAE-pneumonitis compared with those with low CCL5 (*p* = 0.0072, Fisher’s exact test). CCL5, C-C motif chemokine ligand 5; irAE, immune-related adverse event; NR, not reached.

Patients with elevated baseline CCL5 levels (≥150.83 pg/mL) demonstrated significantly shorter overall survival than those with low CCL5 levels (Figure 1A; median OS: 27.6 months vs. not reached; HR = 2.779, 95%CI: 1.017–7.597, *p* = 0.038). However, no significant difference was observed in progression-free survival (PFS) between the two groups (Figure 1B; median PFS: 8.4 vs. 4.2 months; HR = 1.126, 95%CI: 0.465–2.728, *p* = 0.793). Patients who achieved CR or PR exhibited a tendency towards lower baseline concentrations of CCL5 in the plasma compared with those who achieved stable disease (SD) as the best overall response (BoR). However, this difference did not reach statistical significance (Figure S1).

Only grade 1 immune-related pneumonia was observed in our study, which occurred in six patients (18.2%) during treatment. Notably, patients with elevated baseline CCL5 levels had a significantly higher incidence of immune-related pneumonitis than those with low CCL5 levels (Figure 1C,D).

### 3.2. Origin of CCL5 in Peripheral Blood

**Figure 2:**
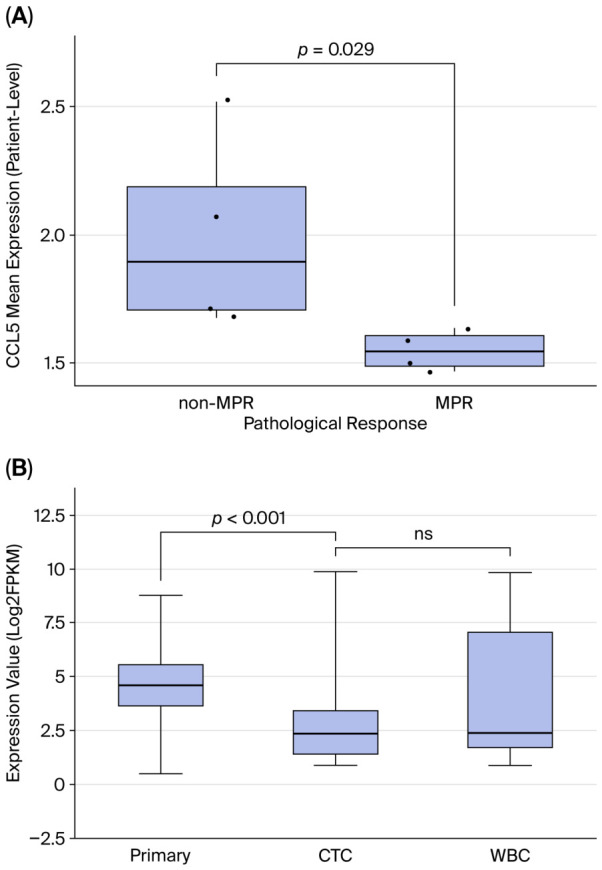
CCL5 expression patterns across sample types and association with pathological responses. (**A**) Box plots showing CCL5 mRNA expression from the baseline single-cell RNA sequencing pseudobulk analysis. Each dot represents the pseudobulk CCL5 expression level aggregated from all T/NK cells within one individual patient’s PBMC sample. Patients achieving a major pathological response (MPR, *n* = 4) exhibited significantly lower CCL5 expression compared with that of non-MPR patients (*n* = 4) (*p* = 0.029, Mann–Whitney U test). (**B**) Box plots comparing CCL5 expression levels (log2TPM) across different sample types using online ctcRbase. Primary tumor tissues showed significantly higher CCL5 expression compared with circulating tumor cells (CTC) (*p* < 0.001), while no significant difference was observed between CTC and white blood cells (WBC). CCL5, C-C motif chemokine ligand 5; CTC, circulating tumor cells; WBC, white blood cells; MPR, major pathological response; TPM, transcripts per million; ns, not significant.

scRNA-seq re-analysis of PBMCs from eight patients with advanced stage NSCLC (using our previously published dataset [29]) who received neoadjuvant immunotherapy plus chemotherapy revealed differential CCL5 expression patterns associated with the treatment response. Patients who failed to achieve a major pathological response (MPR) showed significantly higher T/NK cell CCL5 expression than those who achieved MPR (Figure 2A, *p* = 0.029). This finding corroborates our primary cohort results and suggests that elevated peripheral CCL5 levels may predict poor treatment responses across different clinical settings. The cellular sources were further investigated through re-analysis of PBMC scRNA-seq data, which showed predominant expression in T and NK cells (Figure S7).

CCL5 expression in CTCs was lower than that in primary tumors (Figure 2B, *p* < 0.001). White blood cells (WBC) showed a higher trend of CCL5 expression compared with CTCs (though not statistically significant), supporting the hypothesis that peripheral blood CCL5 primarily derives from immune cell sources.

### 3.3. CCL5 Expression in NSCLC Tumor Tissue

**Figure 3:**
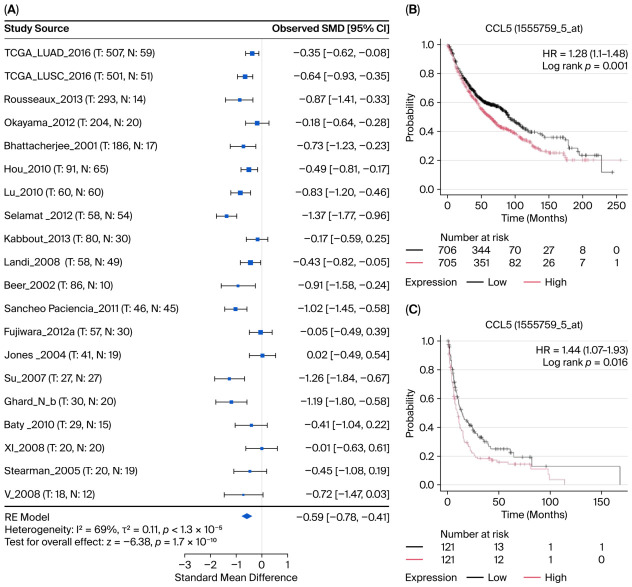
CCL5 expression and prognostic significance in lung cancer based on public database analysis. (**A**) Forest plot showing CCL5 expression differences between normal and lung cancer tissues across multiple datasets from online Lung Cancer Explorer. The standardized mean difference (SMD) with 95% confidence intervals (CI) is displayed for each dataset. Meta-analysis revealed significantly lower CCL5 expression in lung cancer tissues compared with that in normal tissues (pooled SMD = −0.59 [95%CI: −0.81 to −0.38], *p* < 0.001). Individual dataset results are shown with corresponding sample sizes and *p*-values. Heterogeneity statistics: I^2^ = 94%, τ^2^ = 0.15, *p* < 1 × 10^−5^. (**B**) Kaplan–Meier survival curves for overall survival (OS) stratified by CCL5 (gene ID: 1555759_a_at) expression levels in NSCLC patients from the online Kaplan–Meier Plotter database. Numbers at risk are shown at different time points. (**C**) Kaplan–Meier survival curves for progression-free survival (PFS) stratified by CCL5 (gene ID: 1555759_a_at) expression levels in NSCLC patients from the online Kaplan–Meier Plotter database. CCL5, C-C motif chemokine ligand 5; HR, hazard ratio; CI, confidence interval; SMD, standardized mean difference; OS, overall survival; PFS, progression-free survival.

Online lung cancer explorer tools were used to compare the expression of CCL5 in lung cancer and normal lung tissues. CCL5 mRNA expression was significantly lower in NSCLC tumor tissues than in adjacent normal lung tissues, in LUAD and LUSC (Figure 3A, *p* < 0.001). Kaplan–Meier plot analysis of microarray datasets demonstrated that high CCL5 expression in tumor tissues was associated with a worse prognosis in NSCLC patients. High CCL5 expression was linked to shorter OS (Figure 3B, 63 months vs. 90 months, HR = 1.28, 95%CI: 1.1–1.48, *p* = 0.001) in 1411 NSCLC patients. PFS also demonstrated a reduced time to progression in NSCLC patients with high CCL5 expression, with a median of 10 months compared with 14.13 months (Figure 3C, HR = 1.44, 95%CI: 1.07–1.93, *p* = 0.016). Notably, while statistical significance was achieved in the overall NSCLC cohort (*n* = 1411) due to large sample size, the absolute survival difference was modest. Subgroup analysis indicated that this effect was primarily driven by LUAD, with no significant difference observed in LUSC (Figure S2).

### 3.4. Multifaceted Function of CCL5 in TME of NSCLC

#### 3.4.1. CCL5 Attracts Multiple Types of Immune Cell Infiltration into the TME

**Figure 4:**
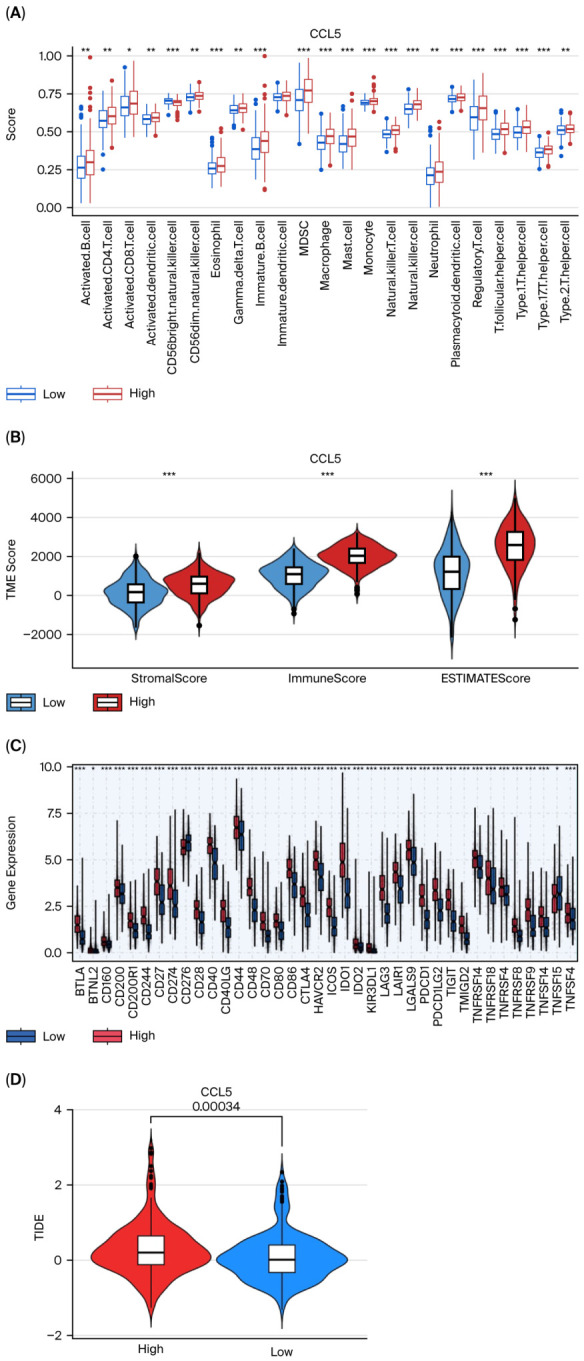
Immune landscape characterization and immunotherapy response prediction associated with CCL5 expression. (**A**) Single-sample gene set enrichment analysis (ssGSEA) comparing immune cell infiltration scores between CCL5 high and low expression groups. Each dot represents the extreme value (minimum or maximum) within the respective group. Multiple immune cell types showed significant differences between groups, with CCL5-high tumors displaying higher infiltrations (* *p* < 0.05, ** *p* < 0.01, *** *p* < 0.001, Wilcoxon rank-sum test). (**B**) Violin plots showing StromalScore, ImmuneScore, and ESTIMATEScore CCL5-high and CCL5-low groups. CCL5-high tumors (red) demonstrated significantly higher scores in stromal score, immune score, and comprehensive score compared with CCL5-low tumors (blue) (*** *p* < 0.001, Mann–Whitney U test). (**C**) Box plots displaying the expression levels of immune checkpoint genes stratified by CCL5 expression (high: red; low: blue). CCL5-high tumors showed significantly elevated expression of multiple immune checkpoint molecules (* *p* < 0.05, *** *p* < 0.001, Wilcoxon rank-sum test). (**D**) Violin plots comparing tumor immune dysfunction and exclusion (TIDE) scores between CCL5-high (red) and CCL5-low (blue) expression groups. CCL5-high tumors exhibited significantly higher TIDE scores (*p* = 0.00034). CCL5, C-C motif chemokine ligand 5; ssGSEA, single-sample gene set enrichment analysis; TIDE, Tumor Immune Dysfunction and Exclusion; MDSC, myeloid-derived suppressor cells.

To further explore the relationship between CCL5 expression and immune cell infiltration, the single-sample gene set enrichment analysis (ssGSEA) method was employed to quantitatively evaluate the infiltration levels of various immune cell subsets in the high and low CCL5 expression groups using TCGA-LUAD and TCGA-LUSC. The ssGSEA results showed that the infiltration levels of most immune cells in the high-expression group of CCL5 were significantly higher than those in the low-expression group, including activated B cells, activated CD4^+^ T cells, activated CD8^+^ T cells, activated dendritic cells (DCs), regulatory T cells (Tregs), myeloid suppressive cells (MDSCs), macrophages, monocytes, natural killer (NK) cells, and various helper T cell subsets. In contrast, the immune infiltration levels in the low-expression group were generally lower (Figure 4A).

Multiple algorithms were used to further investigate the correlation between CCL5 and immune cell infiltration. XCELL, TIMER, QUANTISEQ, MCPcounter, EPIC, CIBERSORT, and CIBERSORT-ABS analysis revealed significant positive correlations between the CCL5 expression and infiltration of multiple immune cell types. The correlation analysis was conducted using the Spearman correlation coefficient (Figure S3). Although these positive correlations are statistically significant, the correlation coefficients are relatively low. This result suggests that while CCL5 plays a role in recruiting anti-tumor (e.g., CD8^+^ T cells) and immunosuppressive cells (e.g., Tregs, MDSCs), it remains only one of many factors shaping this complex tumor microenvironment. The analysis results indicate that the expression level of CCL5 is significantly correlated with the infiltration of various immune cells. Specifically, the expression level shows a positive correlation with CD8^+^ T cells, CD4^+^ memory T cells, activated DCs, macrophages, NK cells, and plasma, suggesting its important role in promoting anti-tumor immune responses. The expression of CCL5 is also positively correlated with some immunosuppressive cells, such as MDSCs and Tregs, indicating that this cytokine may play a dual role in regulating the immune microenvironment.

Subsequently, the matrix score (StromalScore), immune score (ImmuneScore), and comprehensive score (ESTIMATEScore) of each sample were calculated, indirectly reflecting the infiltration of the tumor microenvironment in different CCL5 expression groups. The analysis results showed that the CCL5 high-expression group was significantly higher than the low-expression group regarding StromalScore, ImmuneScore, and ESTIMATEScore (Figure 4B, *p* < 0.001). This result suggests that the high expression of CCL5 is not only closely related to the increase in the level of immune cell infiltration but also significantly correlated with an increase in the proportion of matrix components, indicating that CCL5 may play an important role in shaping the tumor microenvironment, promoting enhanced immune activity and remodeling the tumor stroma.

#### 3.4.2. CCL5 Correlates with Checkpoint Molecule Expression and Immune Escape

The Wilcoxon test demonstrated significant positive correlations between high CCL5 and multiple immune checkpoint molecules expression, including BTLA, CD200R1, CD244, CD274 (PD-L1), CD276, CD28, CTLA4, HAVCR2, IDO1, LAG3, PDCD1 (PD-1), PDCD1LG2 (PD-L2), TIGIT, TNFRSF14, TNFRSF18, TNFRSF4, TNFRSF9, and VTCN1, indicating an immunosuppressive tumor microenvironment (Figure 4C, all *p* < 0.001).

TIDE score analysis indicated that patients with high CCL5 expression had significantly higher TIDE scores than those with low expression (Figure 4D, median TIDE score: 1.47 vs. 0.83, *p* < 0.001), suggesting increased immune evasion potential in CCL5-high tumors.

#### 3.4.3. CCL5 Induces Multiple Anti-Tumor Drug Resistance

Analysis of GDSC databases revealed that high CCL5 expression was associated with decreased sensitivity to multiple targeted therapies and chemotherapeutic drugs commonly used in NSCLC, including cisplatin, vinorelbine, gemcitabine, crizotinib, and savolitinib (Figure S4).

#### 3.4.4. scRNA Analysis Indicates the Complicated Influence of CCL5 Within the TME

**Figure 5:**
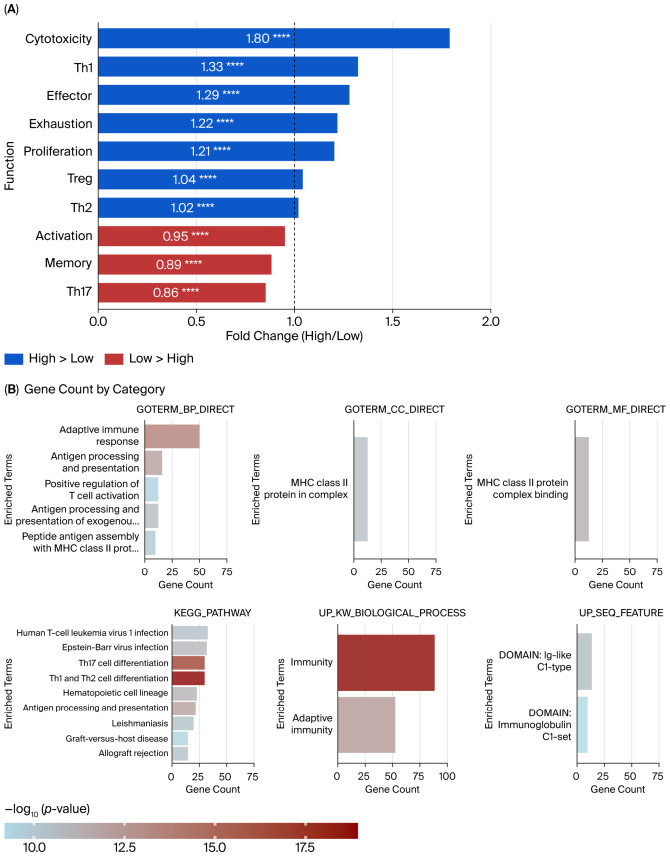
Functional characterization of CCL5-associated immune signatures based on GSE243013 single-cell RNA sequencing data. (**A**) Bar plot showing fold changes in T cell functional states between CCL5-high and CCL5-low groups based on UCell scoring analysis. CCL5-high tumors exhibited significantly elevated scores in cytotoxicity, Th1, effector, exhaustion, proliferation, Treg, and Th2 signatures (blue bars), while CCL5-low tumors showed enrichment in activation, memory, and Th17 signatures (red bars, low > high). All comparisons reached statistical significance (**** *p* < 0.0001, Wilcoxon rank-sum test). The dashed vertical line at fold change = 1.0 indicates the reference baseline. (**B**) Multi-panel bar charts displaying gene counts for enriched pathways stratified by different annotation databases associated with CCL5 expression. Bar colors represent the −log10 (*p*-value) intensity, with darker red indicating higher statistical significance and lighter colors (blue/gray) indicating moderate significance. CCL5, C-C motif chemokine ligand 5; Th, T helper cells; Treg, regulatory T cells. UCell, Universal Cell scoring; MHC, major histocompatibility complex; KEGG, Kyoto Encyclopedia of Genes and Genomes; GO, Gene Ontology; UP, UniProt.

To further investigate the possible underlying mechanism of the intricate modulation of CCL5, we analyzed a publicly available scRNA-seq dataset (GSE243013) with tumor samples collected from 234 patients with advanced stage NSCLC who received neoadjuvant immunotherapy plus chemotherapy post-operatively. The Ucell score method was used to measure the differences in T cell function between different CCL5 expression levels. The cytotoxic, exhaustion, effector, T helper 1 cell (Th1), and Tregs scores were associated with high CCL5 levels, while the activation, memory, and T helper 17 cell (Th17) scores were higher in the low CCL5 group (Figure 5A). DEG and GSEA analysis using the online DAVID tools showed several immune-activating signals among the T cells within the TME, which could partially explain the high frequency of irAEs and high CCL5 level (Figure 5B).

#### 3.4.5. scRNA Analysis Indicates Communication Between T Cells and Myeloid Cells

**Figure 6:**
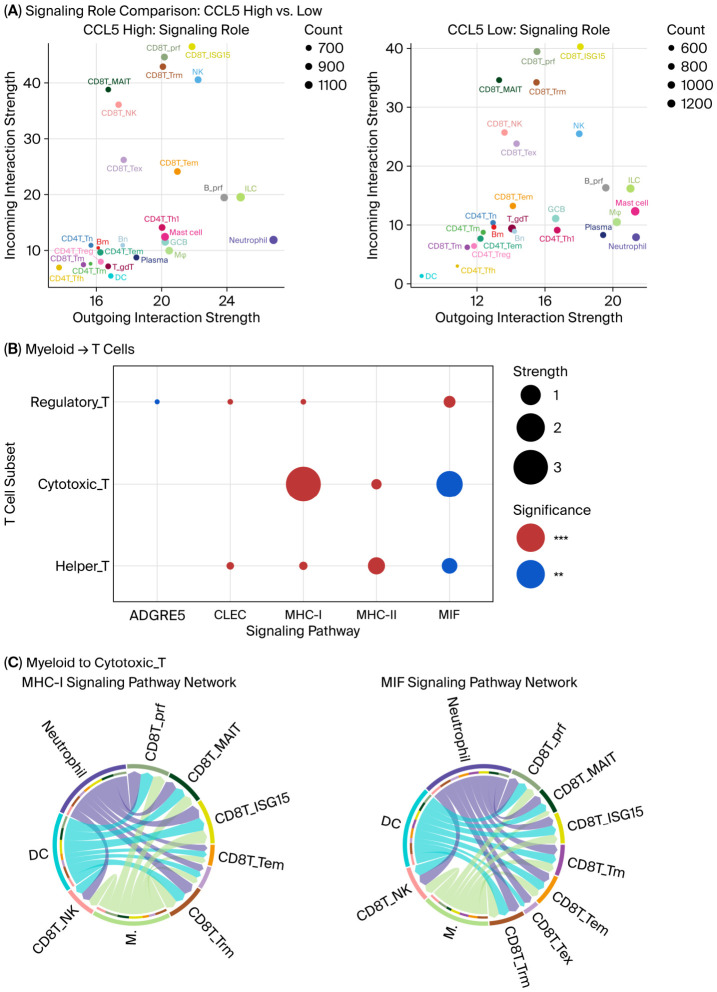

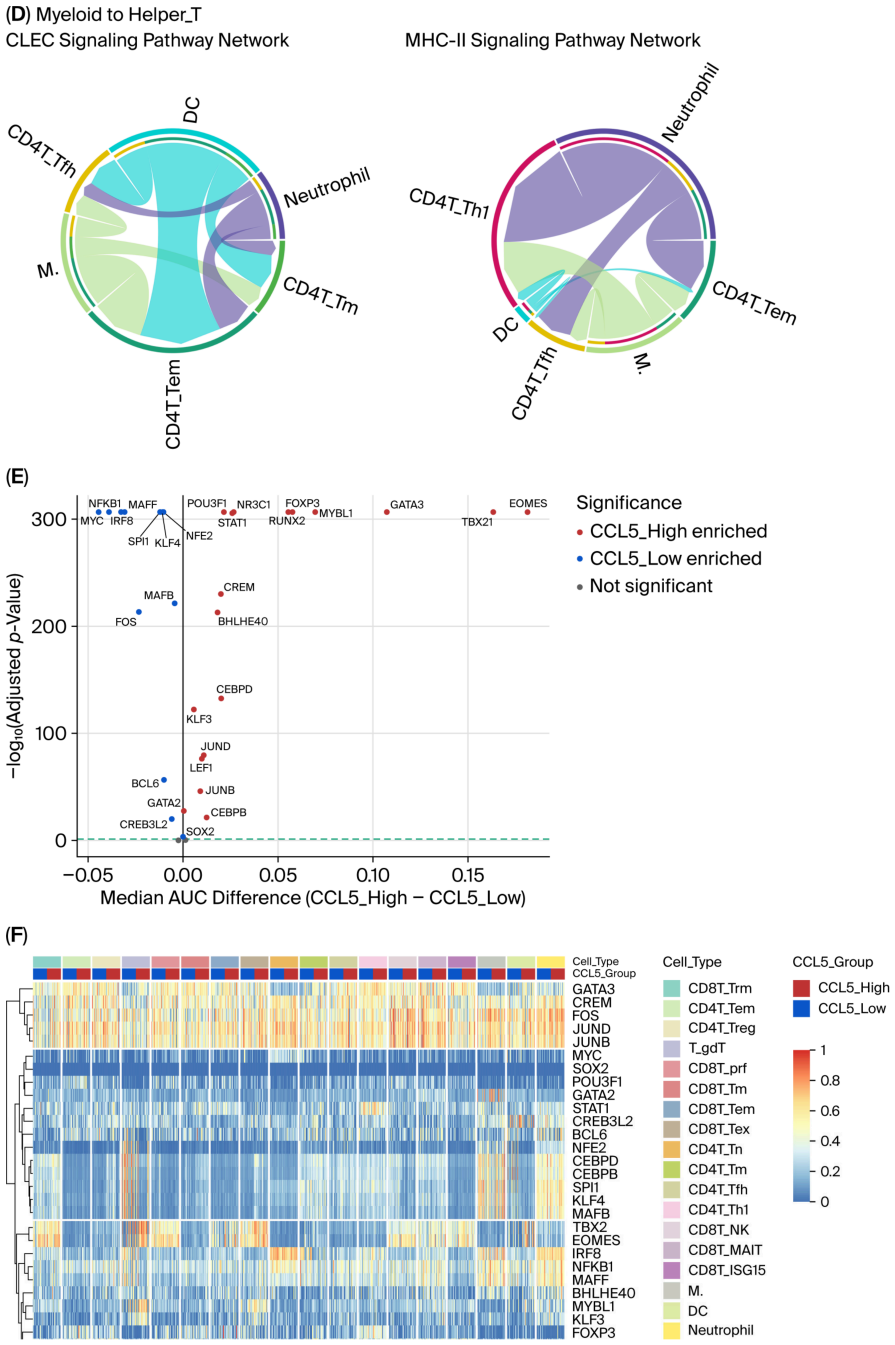
Cell–cell communication and signaling pathway analysis stratified by CCL5 expression. (**A**) Scatter plots comparing the signaling role between CCL5-high and CCL5-low groups based on the CellChat analysis. The left panel presents the outgoing communication strength (sender role) on the *x*-axis and the incoming communication strength (receiver role) on the *y*-axis. (**B**) Bubble plot showing the signaling pathway activity between myeloid cells and T cell subsets (Regulatory_T, Cytotoxic_T, Helper_T). Circle size represents the interaction strength, and the color indicates statistical significance (red: ***, blue: **). (**C**) Chord diagrams illustrating MHC-I and MIF signaling pathway networks from myeloid cells to cytotoxic T cells. The thickness of the ribbons represents signaling strength with colored ribbons indicating communication flow. (**D**) Chord diagrams showing the CLEC and MHC-II signaling pathway networks from myeloid cells to helper T cells. The thickness of the ribbons represents signaling strength, with colored ribbons indicating communication flow. (**E**) Volcano plot presenting the differential expression of ligands and receptors between CCL5-high and CCL5-low groups. The *y*-axis shows −log10 (FDR-adjusted *p*-value), and the *x*-axis represents the median area under the curve (AUC) difference. The dashed horizontal line indicates the FDR significance threshold. (**F**) Heatmap showing transcription factor activity scores across individual cells (columns) for multiple genes (rows). Cells are grouped by CCL5 expression status in the top annotation bar. The CCL5 group classification is shown on the right-side bar (CCL5_High: orange; CCL5_Low: blue). Key transcription factors including GATA3, GEM, FOS, JUND, KLF2, MYC, POU2F1, SATB1, CREM, NFIB, CEBPD, IRF4, MAFB, TBX3, EOMES, NFKB1, NFKB2, BHLHE40, JUNB, BCL6, and FOXP3 show distinct expression patterns between the CCL5-high and CCL5-low groups. Color intensity indicates scaled expression levels (blue: low; orange/red: high). CCL5, C-C motif chemokine ligand 5; MHC, major histocompatibility complex; MIF, macrophage migration inhibitory factor; CLEC, C-type lectin; AUC, area under the curve; FDR, false discovery rate; DC, dendritic cells; M, Macrophages; Tem, effector memory T cells; Trm, tissue-resident memory T cells; Tfh, T follicular helper cells; Th, T helper cells.

CellChat analysis identified enhanced communication networks involving T cell subsets and myeloid cell populations (Figure 6A,B and Data S1, Data S5). The communication strength between T cells and DCs was significantly increased in tumors with high CCL5 expression (Figure 6A). Notably, the major histocompatibility complex I/II (MHC-I/II), macrophage migration inhibitory factor (MIF), and C-type lectin receptor (CLEC) pathways were activated in the CCL5-high group. Among these pathways, MHC-I/II plays a vital role in antigen presentation, while MIF-CD74/CXCR4 and CLEC2B-KLRB1 signaling are always considered immune-suppressive. Details of ligand–receptor binding are shown in Figure 6C,D and the Supplement Materials (Data S4).

**Figure 7:**
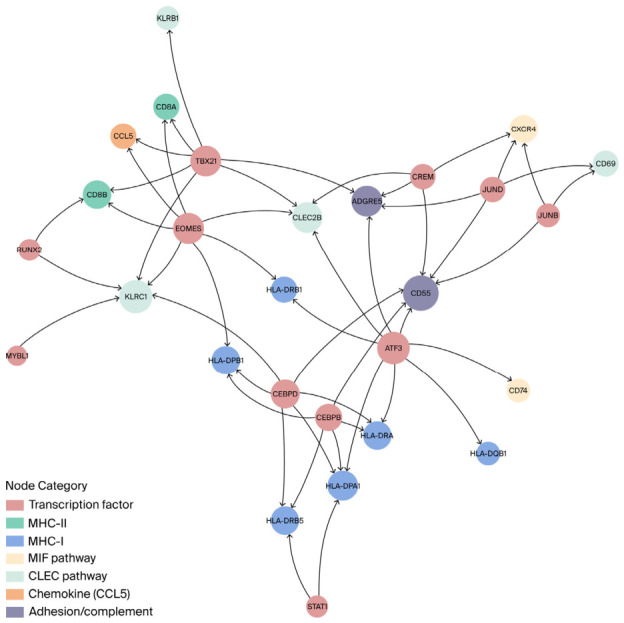
Network visualization of regulons and target genes enriched in CCL5-high expressing samples identified via SCENIC analysis. Edges connecting nodes and direction of the arrows indicate regulatory interactions form regulons towards target genes. The node size reflects the number of predicted regulatory relationships. SCENIC, Single-Cell Regulatory Network Inference and Clustering; CCL5, C-C motif chemokine ligand 5; MHC, major histocompatibility complex; MIF, macrophage migration inhibitory factor; CLEC, C-type lectin receptor.

Subsequent SCENIC network analysis revealed that 31 regulons exhibited differential expression between the high and low CCL5 groups, with statistical significance (Figure 6E,F and Data S3). Differences in the top six upregulated regulons are shown in Figure S6. Notably, EOMES, CREM, CEBPD, TBX21, JUNB, and JUND were implicated in the regulation of the MHC-I/II, MIF-CD74/CXCR4, and CLEC2B/CD69-KLRB1 pathways (Figure 7). Additionally, FOXP3 might be associated with the CCL5-mediated suppressive TME. Detailed information on the regulon-target gene network is provided in the Supplementary Materials (Data S6). The network topology suggests that CCL5 functions as a critical coordinator of immune cell recruitment, activation, effector, exhaustion, and suppressive functions in the tumor microenvironment. This result indicates the potential establishment of a complex network between CCL5 and intricate immune pathways. However, further research is required to elucidate this complex network.

## 4. Discussion

In the treatment of advanced and metastatic NSCLC, a combination of immunotherapy with platinum-based chemotherapy has emerged as the standard first-line therapeutic approach. Recent research focusing on chemokines as immunomodulators within the TME and their impact on the outcome of immunotherapy has garnered notable interest. The value of chemokines in predicting the prognosis and incidence of irAEs in cancer immunotherapy outcomes has also attracted significant attention. Among the identified human chemokines, C-C motif chemokines are crucial in the pathogenesis of inflammatory diseases and cancer [14]. Our clinical findings suggest that elevated initial concentrations of CCL5, a chemokine with diverse functional roles, in the peripheral blood are predictive of reduced OS and an increased risk of immune-related pneumonia in patients with advanced and metastatic NSCLC undergoing treatment with PD-1 inhibitors in combination with chemotherapy. The scRNA data showed elevated CCL5 levels in T/NK cells in PBMC from pretreated NSCLC patients who did not achieve MPR. These findings are consistent with those of previously published studies [22,23,24,25,26]. We verified that the source of CCL5 in the peripheral blood originated from T/NK cells in PBMC rather than from CTCs. Circulating CCL5 serves as an indicator of systemic inflammation or immune activity [20]. The potential use of CCL5 as a prognostic predictor and convenient method for the real-time measurement of irAEs requires further investigation through prospective studies involving larger cohorts.

Within the TME, chemokines play a crucial role in modulating local tumor dynamics [11]. Consequently, chemokines found in peripheral circulation may not adequately capture the intricate functions present within the TME. The results of the survival analysis conducted using the online Kaplan–Meier Plotter with mRNA gene chip data were consistent with our clinical findings. In contrast, a meta-analysis showed decreased CCL5 expression in tumor tissues compared with that in normal lung tissues. This apparent contradiction underscores the complex biological functions of CCL5 in the TME and highlights the importance of considering biomarker compartmentalization in immunotherapy predictions. We used multiple public datasets and models to investigate the underlying mechanisms of these paradoxical findings. High CCL5 levels are associated with chemotherapeutic resistance, elevated immune cell infiltration (encompassing immune-activating and immune-suppressive cells), the increased expression of immune checkpoint molecules, and a high immune escape score. Although direct immunohistochemistry (IHC) to localize CCL5-expressing cells within the neoplastic epithelial compartment was not performed in this retrospective study due to limited remaining tissue samples, single-cell RNA-seq analysis of the publicly available GSE243013 dataset provided high-resolution evidence of CCL5 expression patterns and intercellular communication within the TME, including potential interactions that may influence neoplastic epithelial cells. This orthogonal bioinformatics approach complements our clinical findings and supports the observed paradoxical immune landscape.

The complex role of CCL5 may be partially attributed to its diverse receptors expressed on various immune cells. CCL5 initiates a signaling response that is specific to certain cell types by binding to distinct G protein-coupled receptors, including CCR1, CCR3, CCR4, and CCR5, which are present on the surfaces of target cells [11,32]. CD44 acts as a co-receptor for CCL5 [33]. Additionally, CCL5 influences tumor development either through autocrine or paracrine mechanisms. CCL5 can directly impact cancer cell growth, movement, and survival via its autocrine role or indirectly modify the TME by attracting inflammatory cells through its paracrine function, thereby adapting the TME to support its own persistence [34]. CCL5 is produced and released by macrophages, T and NK cells, tubular epithelial cells, synovial fibroblasts, and certain cancer cell types [35]. CCL5 facilitates the participation of various cells in the inflammatory process, including the migration and localization of T and NK cells [36]. Notably, CCL5 can attract a range of myeloid cell types, such as monocytes, macrophages, mast cells, eosinophils, basophils, and DCs [37,38]. The expression of CCL5 in breast cancer not only facilitated the migration of Treg cells to tumor sites but also augmented the cytotoxic capacity of Treg cells against CD8+ T cells [39]. CCL5 is involved in the development of new blood vessels and is associated with increased levels of vascular endothelial growth factor in cancerous and vascular endothelial cells [40], which have been demonstrated to facilitate immune evasion [41]. In colorectal cancer (CRC), CCL5 stabilizes PD-L1 expression by facilitating the formation of the nuclear factor kappa (NF-κ)-p65/signal transducer and activator of transcription (STAT3) complex, which enhances COP9 signal 5 (CSN5) gene expression. CSN5 is responsible for the deubiquitination of PD-L1. Elevated CSN5 levels in CRC patients are associated with notably reduced survival duration [42]. The expression of PD-L1 is well-known to be correlated with an improved prognosis in patients undergoing anti-PD-1 antibody monotherapy for NSCLC [43]. Our study suggests that CCL5 may mediate resistance to platinum-based chemotherapy, indicating the complex influence of CCL5 on the TME. GSEA reveals several immune-activating signals among the T cells within the TME. A pro-inflammatory microenvironment contributes to favorable conditions for irAEs [44]. In cases of CRC, variations in the CCL5/CCR5 pathway can serve as indicators for predicting severe hand–foot skin reactions in patients with metastatic CRC undergoing treatment with regorafenib [45]. The increased state of inflammation might play a role in the onset of irAEs in cancer patients receiving immunotherapy.

To further elucidate the mechanism by which CCL5 modulates the TME in advanced stages, we performed a single-cell RNA sequencing analysis using a dataset from the GEO public repository. Our findings indicate enhanced communication between T cells and DCs, with increased activity in the major MHC-I/II, MIF, and CLEC pathways in the CCL5-high group. MHC-I molecules are essential for the immune system as they present peptides on the cell surface, allowing CD8+ T cells to detect and eliminate cancerous or infected cells [46]. CCL5 plays a traditional role in the immune response by enhancing MHC-I antigen presentation in infectious diseases. This outcome is achieved by promoting the migration and maturation of DCs, which, in turn, activates CD8+ cytotoxic T cells [47]. In cancerous diseases, increased activity of the MHC-I pathway is often linked to a better prognosis because of the effectiveness of CD8+ T cells [48]. Meanwhile, the MHC-II pathway between antigen-presenting cells and CD4+ helper T cells orchestrates an adaptive immune response [49], which also falls within the indirect modulatory spectrum of CCL5 in the context of infectious and cancerous diseases [50,51]. Based on the present study, we conclude that CCL5 partially enhances the MHC-I/II-mediated immune response within the TME. However, we observed an elevated infiltration frequency of immune-suppressive cells and inhibitory signals, such as the MIF and CLEC pathways. MIF itself serves as a multifaceted chemokine in physiological and pathological processes [52]. Moreover, the interaction of MIF with various receptor complexes, such as CD74/CD44 and CD74/CXCR4, determines its distinct biological effects, which can range from the chemotaxis of immune cells to the proliferation of cells, underscoring a complex level of functional regulation [52]. Notably, CD74 serves as an invariant chain of MHC-II, avoiding the premature binding of MHC-II to external antigen peptides while transporting them from the endoplasmic reticulum (ER) to the Golgi apparatus within antigen presentation cells [53]. Previous research suggested that MIF impairs the antigen presentation from DCs to CD4+ T cells by downregulating MHC-II in melanoma [54]. Targeting the MIF-CD74 axis was demonstrated to enhance the effectiveness of anti-tumor therapies in NSCLC and triple-negative breast cancer (TNBC) [55,56]. We observed that the CLEC2B/CD69-KLRB1 pathway may also impede the immune response within the TME, as this pathway is recognized as a suppressive signal that downregulates the activity of T and NK cells in ESCC [57]. Collectively, elevated CCL5 levels contribute to an immunosuppressive tumor TME in advanced NSCLC by facilitating the infiltration of immune-suppressive cells and activating immune-suppressive signaling pathways. Further SCENIC analysis indicated that the transcriptons FOXP3, EOMES, CEBPD, CREM, TBX21, JUNB, and JUND might be associated with the CCL5-mediated suppressive immune TME. We hypothesize that high CCL5 levels induce a complicated TME and regulate the expression of CD74 and CXCR4, which are the targets of CREM and CEBPD. EOMES, JUNB/D, and TBX21 are involved in the regulation of the CLEC2B/CD69-KLRB1 and MHC-I/II pathways. CCL5 was found to be target of EOMES, which is considered an inhibitor of sustained T cell-mediated anti-tumor immunity [58]. FOXP3, a key transcription factor in regulatory Tregs, plays a pivotal role in promoting immune suppression within the TME by inhibiting effector T cell activity. The expression of FOXP3 in Tregs and tumor cells facilitates tumor immune escape, correlating with poor prognosis and metastasis [59]. Further investigation is required to unveil this intricate network.

Beyond its role as a biomarker, CCL5 presents a promising therapeutic target for overcoming immunotherapy resistance in NSCLC. Our finding that CCL5 recruits effector and suppressor cells suggests that simply blocking CCL5 might be a double-edged sword. The role of CCL5 in NSCLC is considered “paradoxical” because it simultaneously promotes the infiltration of anti-tumor effector cells (CD8^+^ T cells, NK cells, activated dendritic cells) and immunosuppressive populations (Tregs, MDSCs). This dual recruitment leads to elevated immune checkpoint expression, higher TIDE scores, and potential resistance despite increased overall immune infiltration. Specific targeting of the CCL5-CCR5 axis (e.g., using the CCR5 antagonist Maraviroc) in combination with PD-1 blockade could potentially reduce the recruitment of Tregs and MDSCs while maintaining or enhancing effector T cell activity [60,61]. Furthermore, CCL5 levels could be integrated into patient stratification strategies. Patients with high baseline peripheral CCL5 might benefit from more intensive monitoring for immune-related pneumonitis and could be candidates for earlier intervention with CCR5 inhibitors to remodel the TME from a ‘paradoxical’ to a ‘pro-inflammatory’ state.

However, several limitations of this study should be acknowledged. First, the size of our discovery clinical cohort (*n* = 33) is relatively small, which may constrain the statistical power of the survival and toxicity analyses. To mitigate this limitation, we employed an integrated multi-omics approach, validating our findings across multiple independent public datasets (TCGA and GSE243013), which showed high consistency with our clinical observations. Second, although re-analysis of the *n* = 8 PBMC scRNA-seq dataset identified T/NK cells as the primary source of circulating CCL5, we did not perform T cell receptor (TCR) repertoire sequencing in the current study. Such analysis in future work could help trace the clonal origin and functional state (e.g., tumor-reactive versus exhausted) of these CCL5-expressing T cells, thereby strengthening the mechanistic link to poorer overall survival in the *n* = 33 cohort. Third, due to limited tissue availability, we did not perform immunohistochemistry or spatial transcriptomics to directly localize CCL5 within the neoplastic compartment. Future studies incorporating TCR sequencing, IHC, or spatial transcriptomics, together with larger prospective cohorts and dedicated in vitro/in vivo functional assays, are warranted to fully elucidate how CCL5 shifts from an immune-activating to an immune-suppressive mediator in NSCLC.

## 5. Conclusions

This study highlights the potential of circulating CCL5 as a predictive biomarker for clinical outcomes and immune-related toxicity in advanced NSCLC patients receiving chemoimmunotherapy. Supported by multi-omics re-analyses of single-cell datasets, our findings raise the hypothesis that CCL5 orchestrates a paradoxical tumor microenvironment by simultaneously recruiting immune-activating and immunosuppressive cells, thereby driving therapeutic resistance. While these observations provide valuable mechanistic insights and suggest potential combination strategies targeting the CCL5 axis, the present study is primarily hypothesis-generating. Future investigations, including TCR repertoire analysis to trace peripheral T cell clones expressing CCL5, larger prospective clinical cohorts, and rigorous experimental validation (e.g., IHC and functional assays), will be essential to firmly establish the clinical utility of CCL5 as a robust biomarker and therapeutic target in NSCLC chemoimmunotherapy.

## Data Availability

The data presented in this study include publicly available data from NCBI GEO (https://www.ncbi.nlm.nih.gov/geo) and TCGA (https://www.cancer.gov/ccg/research/genome-sequencing/tcga). Patient-specific data are available on request from the corresponding authors due to privacy and ethical restrictions (patient confidentiality and applicable data protection regulations). The patient data are not publicly available to protect the privacy of the participants.

## Supplementary Materials

The following supporting information can be downloaded at: https://www.mdpi.com/article/10.3390/cancers18081271/s1. Supplementary Figures: Figure S1: Comparison of plasma CCL5 levels between patients with different responses; Figure S2: Association between CCL5 expression and survival outcomes in lung adenocarcinoma and lung squamous cell carcinoma; Figure S3: CCL5 orchestrates dual immune cell infiltration patterns in NSCLC; Figure S4: Association between CCL5 expression and drug sensitivity across multiple anti-cancer therapeutic agents; Figure S5: Differential cell–cell communication patterns between CCL5 high and low expression groups revealed with CellChat analysis; Figure S6: Comparison of regulon activity between high and low CCL5 expression groups in top six differentially active regulons. Figure S7: Violin plot showing CCL5 mRNA expression across major immune cell types in PBMCs. Supplementary Data: Data S1: CCL5_Master_LR_Communications.csv; Data S2: Ucell_gene_Signature.csv; Data S3: ccl5_regulon_diff_analysis.csv; Data S4: Cellchat_supplementary_materials.pdf; Data S5: CCL5_Upregulated_CellChat.csv; Data S6: regulon_target_info_complete.csv.

## References

[1] Sung H., Ferlay J., Siegel R.L., Laversanne M., Soerjomataram I., Jemal A., Bray F. (2021). Global Cancer Statistics 2020: GLOBOCAN Estimates of Incidence and Mortality Worldwide for 36 Cancers in 185 Countries. CA A Cancer J. Clin..

[2] Gridelli C., Rossi A., Carbone D.P., Guarize J., Karachaliou N., Mok T., Petrella F., Spaggiari L., Rosell R. (2015). Non-small-cell lung cancer. Nat. Rev. Dis. Primers.

[3] Jeon H., Wang S., Song J., Gill H., Cheng H. (2025). Update 2025: Management of Non-Small-Cell Lung Cancer. Lung.

[4] Reck M., Ciuleanu T.E., Cobo M., Schenker M., Zurawski B., Menezes J., Richardet E., Bennouna J., Felip E., Juan-Vidal O. (2021). First-line nivolumab plus ipilimumab with two cycles of chemotherapy versus chemotherapy alone (four cycles) in advanced non-small-cell lung cancer: CheckMate 9LA 2-year update. ESMO Open.

[5] Garassino M.C., Gadgeel S., Speranza G., Felip E., Esteban E., Dómine M., Hochmair M.J., Powell S.F., Bischoff H.G., Peled N. (2023). Pembrolizumab Plus Pemetrexed and Platinum in Nonsquamous Non-Small-Cell Lung Cancer: 5-Year Outcomes From the Phase 3 KEYNOTE-189 Study. J. Clin. Oncol. Off. J. Am. Soc. Clin. Oncol..

[6] Rother C., John T., Wong A. (2024). Biomarkers for immunotherapy resistance in non-small cell lung cancer. Front. Oncol..

[7] Reck M., Frost N., Peters S., Fox B.A., Ferrara R., Savai R., Barlesi F. (2025). Treatment of NSCLC after chemoimmunotherapy-are we making headway?. Nat. Rev. Clin. Oncol..

[8] Yang X., Luo B., Tian J., Wang Y., Lu X., Ni J., Yang Y., Jiang L., Ren S. (2025). Biomarkers and ImmuneScores in lung cancer: Predictive insights for immunotherapy and combination treatment strategies. Biol. Proced. Online.

[9] Rached L., Laparra A., Sakkal M., Danlos F.X., Barlesi F., Carbonnel F., De Martin E., Ducreux M., Even C., Le Pavec J. (2024). Toxicity of immunotherapy combinations with chemotherapy across tumor indications: Current knowledge and practical recommendations. Cancer Treat. Rev..

[10] Bader J.E., Voss K., Rathmell J.C. (2020). Targeting Metabolism to Improve the Tumor Microenvironment for Cancer Immunotherapy. Mol. Cell.

[11] Nagarsheth N., Wicha M.S., Zou W. (2017). Chemokines in the cancer microenvironment and their relevance in cancer immunotherapy. Nat. Rev. Immunol..

[12] Mohs A., Kuttkat N., Reißing J., Zimmermann H.W., Sonntag R., Proudfoot A., Youssef S.A., de Bruin A., Cubero F.J., Trautwein C. (2017). Functional role of CCL5/RANTES for HCC progression during chronic liver disease. J. Hepatol..

[13] Marques R.E., Guabiraba R., Russo R.C., Teixeira M.M. (2013). Targeting CCL5 in inflammation. Expert. Opin. Ther. Targets.

[14] Zeng Z., Lan T., Wei Y., Wei X. (2022). CCL5/CCR5 axis in human diseases and related treatments. Genes. Dis..

[15] Böttcher J.P., Bonavita E., Chakravarty P., Blees H., Cabeza-Cabrerizo M., Sammicheli S., Rogers N.C., Sahai E., Zelenay S., Reis e Sousa C. (2018). NK Cells Stimulate Recruitment of cDC1 into the Tumor Microenvironment Promoting Cancer Immune Control. Cell.

[16] Araujo J.M., Gomez A.C., Aguilar A., Salgado R., Balko J.M., Bravo L., Doimi F., Bretel D., Morante Z., Flores C. (2018). Effect of CCL5 expression in the recruitment of immune cells in triple negative breast cancer. Sci. Rep..

[17] Melese E.S., Franks E., Cederberg R.A., Harbourne B.T., Shi R., Wadsworth B.J., Collier J.L., Halvorsen E.C., Johnson F., Luu J. (2022). CCL5 production in lung cancer cells leads to an altered immune microenvironment and promotes tumor development. Oncoimmunology.

[18] Zhang Y., Lv D., Kim H.J., Kurt R.A., Bu W., Li Y., Ma X. (2013). A novel role of hematopoietic CCL5 in promoting triple-negative mammary tumor progression by regulating generation of myeloid-derived suppressor cells. Cell Res..

[19] Umansky V., Blattner C., Gebhardt C., Utikal J. (2017). CCR5 in recruitment and activation of myeloid-derived suppressor cells in melanoma. Cancer Immunol. Immunother..

[20] Ji S., Chen H., Yang K., Zhang G., Mao B., Hu Y., Zhang H., Xu J. (2020). Peripheral cytokine levels as predictive biomarkers of benefit from immune checkpoint inhibitors in cancer therapy. Biomed. Pharmacother..

[21] Chen Y.C., Zheng W.Z., Liu C.P., Zhao Y.Q., Li J.W., Du Z.S., Zhai T.T., Lin H.Y., Shi W.Q., Cai S.Q. (2024). Pan-cancer analysis reveals CCL5/CSF2 as potential predictive biomarkers for immune checkpoint inhibitors. Cancer Cell Int..

[22] Taylor C., Cheema A.S., Asleh K., Finn N., Abdelsalam M., Ouellette R.J. (2025). Sex-specific cytokine signatures as predictors of anti-PD1 therapy response in non-small cell lung cancer. Front. Immunol..

[23] Tsai Y.T., Schlom J., Donahue R.N. (2024). Blood-based biomarkers in patients with non-small cell lung cancer treated with immune checkpoint blockade. J. Exp. Clin. Cancer Res..

[24] Umekawa K., Kimura T., Kudoh S., Suzumura T., Oka T., Nagata M., Mitsuoka S., Matsuura K., Nakai T., Yoshimura N. (2013). Plasma RANTES, IL-10, and IL-8 levels in non-small-cell lung cancer patients treated with EGFR-TKIs. BMC Res. Notes.

[25] Huang C.Y., Fong Y.C., Lee C.Y., Chen M.Y., Tsai H.C., Hsu H.C., Tang C.H. (2009). CCL5 increases lung cancer migration via PI3K, Akt and NF-kappaB pathways. Biochem. Pharmacol..

[26] Oyanagi J., Koh Y., Sato K., Mori K., Teraoka S., Akamatsu H., Kanai K., Hayata A., Tokudome N., Akamatsu K. (2019). Predictive value of serum protein levels in patients with advanced non-small cell lung cancer treated with nivolumab. Lung Cancer.

[27] Luo J., Cheng K., Ji X., Gao C., Zhu R., Chen J., Xue W., Huang Q., Xu Q. (2024). Anlotinib enhanced CD8(+) T cell infiltration via induction of CCL5 improves the efficacy of PD-1/PD-L1 blockade therapy in lung cancer. Cancer Lett..

[28] Zhou L., Xiong Y., Wang Y., Meng Y., Zhang W., Shen M., Zhang X., Li S., Ren B., Li R. (2022). A Phase IB Trial of Autologous Cytokine-Induced Killer Cells in Combination with Sintilimab, Monoclonal Antibody Against Programmed Cell Death-1, plus Chemotherapy in Patients with Advanced Non-Small-Cell Lung Cancer. Clin. Lung Cancer.

[29] Hui Z., Zhang J., Ren Y., Li X., Yan C., Yu W., Wang T., Xiao S., Chen Y., Zhang R. (2022). Single-cell profiling of immune cells after neoadjuvant pembrolizumab and chemotherapy in IIIA non-small cell lung cancer (NSCLC). Cell Death Dis..

[30] Charoentong P., Finotello F., Angelova M., Mayer C., Efremova M., Rieder D., Hackl H., Trajanoski Z. (2017). Pan-cancer Immunogenomic Analyses Reveal Genotype-Immunophenotype Relationships and Predictors of Response to Checkpoint Blockade. Cell Rep..

[31] Bindea G., Mlecnik B., Tosolini M., Kirilovsky A., Waldner M., Obenauf A.C., Angell H., Fredriksen T., Lafontaine L., Berger A. (2013). Spatiotemporal dynamics of intratumoral immune cells reveal the immune landscape in human cancer. Immunity.

[32] Bronte V., Bria E. (2016). Interfering with CCL5/CCR5 at the Tumor-Stroma Interface. Cancer Cell.

[33] Roscic-Mrkic B., Fischer M., Leemann C., Manrique A., Gordon C.J., Moore J.P., Proudfoot A.E., Trkola A. (2003). RANTES (CCL5) uses the proteoglycan CD44 as an auxiliary receptor to mediate cellular activation signals and HIV-1 enhancement. Blood.

[34] Broekman M.L., Maas S.L.N., Abels E.R., Mempel T.R., Krichevsky A.M., Breakefield X.O. (2018). Multidimensional communication in the microenvirons of glioblastoma. Nat. Rev. Neurol..

[35] Soria G., Ben-Baruch A. (2008). The inflammatory chemokines CCL2 and CCL5 in breast cancer. Cancer Lett..

[36] Hinrichs A.C., Blokland S.L.M., Kruize A.A., Lafeber F.P.J., Leavis H.L., van Roon J.A.G. (2022). CCL5 Release by CCR9+ CD8 T Cells: A Potential Contributor to Immunopathology of Primary Sjögren’s Syndrome. Front. Immunol..

[37] Aldinucci D., Casagrande N. (2018). Inhibition of the CCL5/CCR5 Axis against the Progression of Gastric Cancer. Int. J. Mol. Sci..

[38] Marcuzzi E., Angioni R., Molon B., Calì B. (2018). Chemokines and Chemokine Receptors: Orchestrating Tumor Metastasization. Int. J. Mol. Sci..

[39] Chang L.Y., Lin Y.C., Mahalingam J., Huang C.T., Chen T.W., Kang C.W., Peng H.M., Chu Y.Y., Chiang J.M., Dutta A. (2012). Tumor-derived chemokine CCL5 enhances TGF-β-mediated killing of CD8(+) T cells in colon cancer by T-regulatory cells. Cancer Res..

[40] Sax M.J., Gasch C., Athota V.R., Freeman R., Rasighaemi P., Westcott D.E., Day C.J., Nikolic I., Elsworth B., Wei M. (2016). Cancer cell CCL5 mediates bone marrow independent angiogenesis in breast cancer. Oncotarget.

[41] Tzeng H.T., Huang Y.J. (2023). Tumor Vasculature as an Emerging Pharmacological Target to Promote Anti-Tumor Immunity. Int. J. Mol. Sci..

[42] Liu C., Yao Z., Wang J., Zhang W., Yang Y., Zhang Y., Qu X., Zhu Y., Zou J., Peng S. (2020). Macrophage-derived CCL5 facilitates immune escape of colorectal cancer cells via the p65/STAT3-CSN5-PD-L1 pathway. Cell Death Differ..

[43] Xu Y., Wan B., Chen X., Zhan P., Zhao Y., Zhang T., Liu H., Afzal M.Z., Dermime S., Hochwald S.N. (2019). The association of PD-L1 expression with the efficacy of anti-PD-1/PD-L1 immunotherapy and survival of non-small cell lung cancer patients: A meta-analysis of randomized controlled trials. Transl. Lung Cancer Res..

[44] Bardoscia L., Pasinetti N., Triggiani L., Cozzi S., Sardaro A. (2021). Biological Bases of Immune-Related Adverse Events and Potential Crosslinks With Immunogenic Effects of Radiation. Front. Pharmacol..

[45] Suenaga M., Schirripa M., Cao S., Zhang W., Yang D., Ning Y., Cremolini C., Antoniotti C., Borelli B., Mashima T. (2018). Gene Polymorphisms in the CCL5/CCR5 Pathway as a Genetic Biomarker for Outcome and Hand-Foot Skin Reaction in Metastatic Colorectal Cancer Patients Treated With Regorafenib. Clin. Color. Cancer.

[46] Sykulev Y. (2023). Factors contributing to the potency of CD8(+) T cells. Trends Immunol..

[47] Crawford A., Angelosanto J.M., Nadwodny K.L., Blackburn S.D., Wherry E.J. (2011). A role for the chemokine RANTES in regulating CD8 T cell responses during chronic viral infection. PLoS Pathog..

[48] Turcotte S., Katz S.C., Shia J., Jarnagin W.R., Kingham T.P., Allen P.J., Fong Y., D’Angelica M.I., DeMatteo R.P. (2014). Tumor MHC class I expression improves the prognostic value of T-cell density in resected colorectal liver metastases. Cancer Immunol. Res..

[49] Alspach E., Lussier D.M., Miceli A.P., Kizhvatov I., DuPage M., Luoma A.M., Meng W., Lichti C.F., Esaulova E., Vomund A.N. (2019). MHC-II neoantigens shape tumour immunity and response to immunotherapy. Nature.

[50] Nesbeth Y.C., Martinez D.G., Toraya S., Scarlett U.K., Cubillos-Ruiz J.R., Rutkowski M.R., Conejo-Garcia J.R. (2010). CD4+ T cells elicit host immune responses to MHC class II-negative ovarian cancer through CCL5 secretion and CD40-mediated licensing of dendritic cells. J. Immunol..

[51] Ottaviani C., Nasorri F., Bedini C., de Pità O., Girolomoni G., Cavani A. (2006). CD56brightCD16(-) NK cells accumulate in psoriatic skin in response to CXCL10 and CCL5 and exacerbate skin inflammation. Eur. J. Immunol..

[52] Sumaiya K., Langford D., Natarajaseenivasan K., Shanmughapriya S. (2022). Macrophage migration inhibitory factor (MIF): A multifaceted cytokine regulated by genetic and physiological strategies. Pharmacol. Ther..

[53] Germain R.N. (2011). Uncovering the role of invariant chain in controlling MHC class II antigen capture. J. Immunol..

[54] Figueiredo C.R., Azevedo R.A., Mousdell S., Resende-Lara P.T., Ireland L., Santos A., Girola N., Cunha R., Schmid M.C., Polonelli L. (2018). Blockade of MIF-CD74 Signalling on Macrophages and Dendritic Cells Restores the Antitumour Immune Response Against Metastatic Melanoma. Front. Immunol..

[55] Pellegrino B., David K., Rabani S., Lampert B., Tran T., Doherty E., Piecychna M., Meza-Romero R., Leng L., Hershkovitz D. (2024). CD74 promotes the formation of an immunosuppressive tumor microenvironment in triple-negative breast cancer in mice by inducing the expansion of tolerogenic dendritic cells and regulatory B cells. PLoS Biol..

[56] Liu L., Wang J., Wang Y., Chen L., Peng L., Bin Y., Ding P., Zhang R., Tong F., Dong X. (2024). Blocking the MIF-CD74 axis augments radiotherapy efficacy for brain metastasis in NSCLC via synergistically promoting microglia M1 polarization. J. Exp. Clin. Cancer Res..

[57] Yang J., Wu B., Li G., Zhang C., Xie Y., Kong W., Zeng Z. (2024). Landscape of epithelial cell subpopulations in the human esophageal squamous cell carcinoma microenvironment. Heliyon.

[58] Sun R., Wu Y., Zhou H., Wu Y., Yang Z., Gu Y., Jiang J., Lu B., Zhu Y. (2021). Eomes Impedes Durable Response to Tumor Immunotherapy by Inhibiting Stemness, Tissue Residency, and Promoting the Dysfunctional State of Intratumoral CD8(+) T Cells. Front. Cell Dev. Biol..

[59] Wang J., Gong R., Zhao C., Lei K., Sun X., Ren H. (2023). Human FOXP3 and tumour microenvironment. Immunology.

[60] Halama N., Zoernig I., Berthel A., Kahlert C., Klupp F., Suarez-Carmona M., Suetterlin T., Brand K., Krauss J., Lasitschka F. (2016). Tumoral Immune Cell Exploitation in Colorectal Cancer Metastases Can Be Targeted Effectively by Anti-CCR5 Therapy in Cancer Patients. Cancer Cell.

[61] Jiao X., Nawab O., Patel T., Kossenkov A.V., Halama N., Jaeger D., Pestell R.G. (2019). Recent Advances Targeting CCR5 for Cancer and Its Role in Immuno-Oncology. Cancer Res..

