# Supplementary material for: CCL5 Orchestrates Paradoxical Immune Landscapes in NSCLC: Simultaneous Recruitment of Effector and Suppressor Cells Shapes Immunotherapy Resistance
Source: Cancers (Basel). 2026 Apr 16;18(8):1271. doi: 10.3390/cancers18081271 (PMC13114919; doi:10.3390/cancers18081271)

Cell Communication Analysis: Myeloid to Helper_T

Pathway: MHC−II

CCL5 High vs Low Expression Group Comparison

MHC−II signaling pathway network

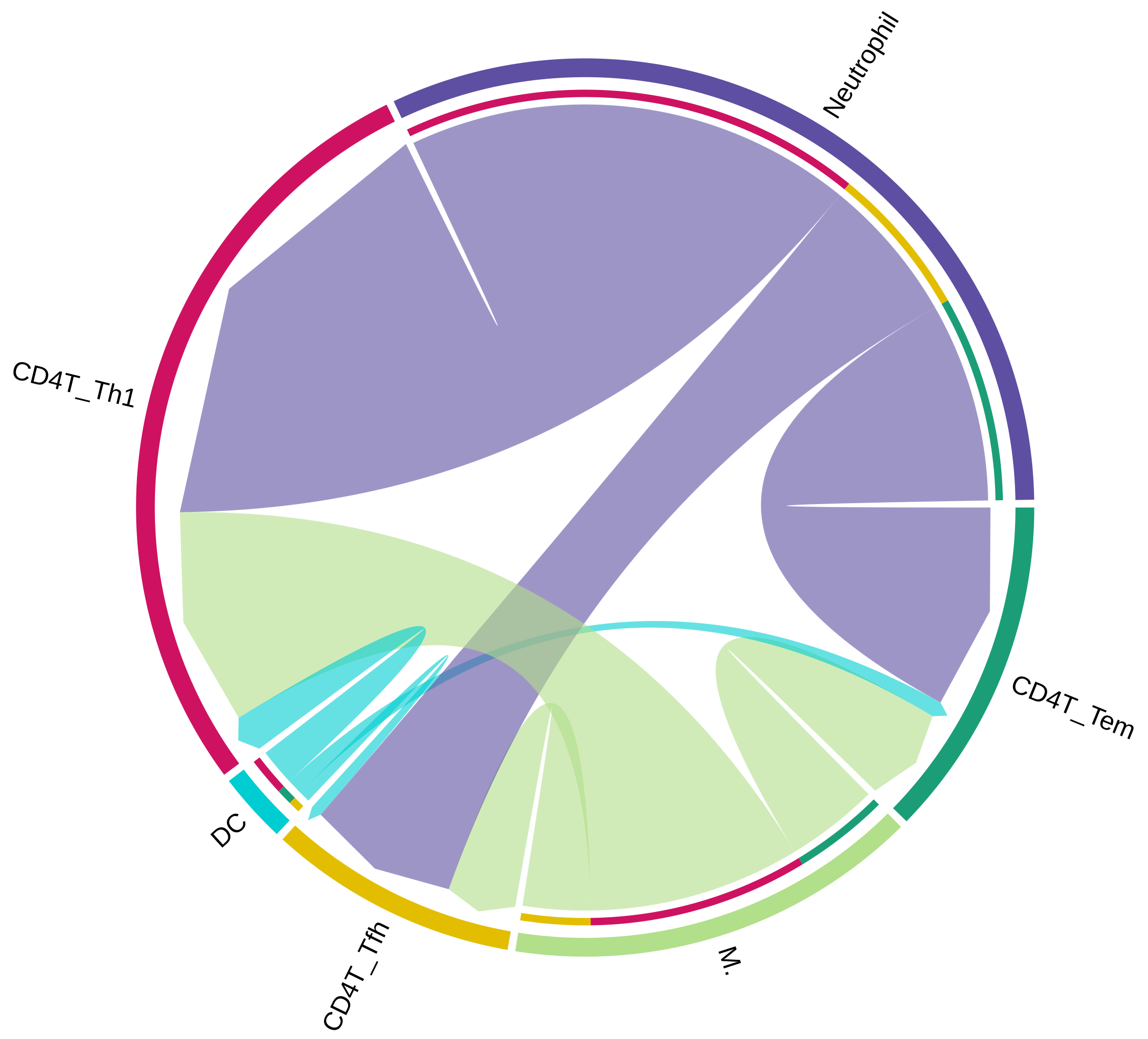

MHC−II signaling pathway network

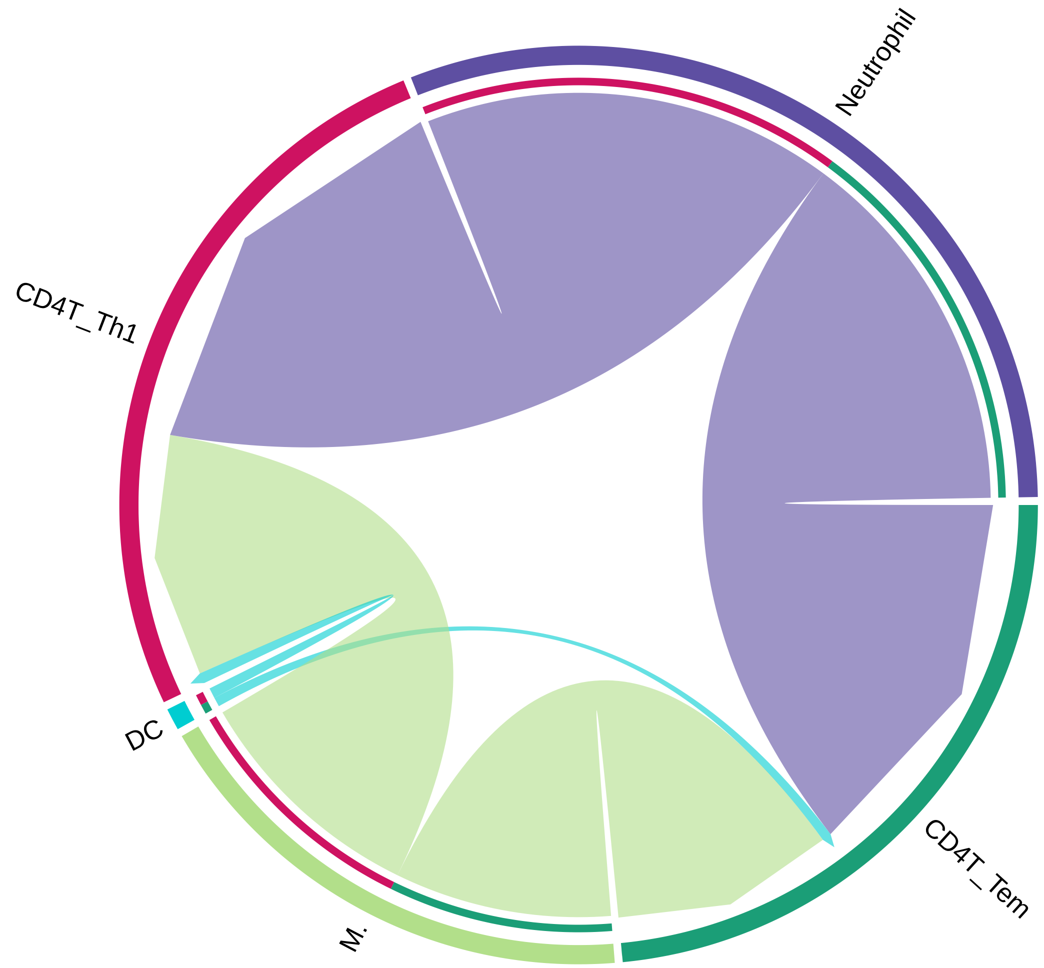

3

3

CCL5 High : MHC−II L−R pairs

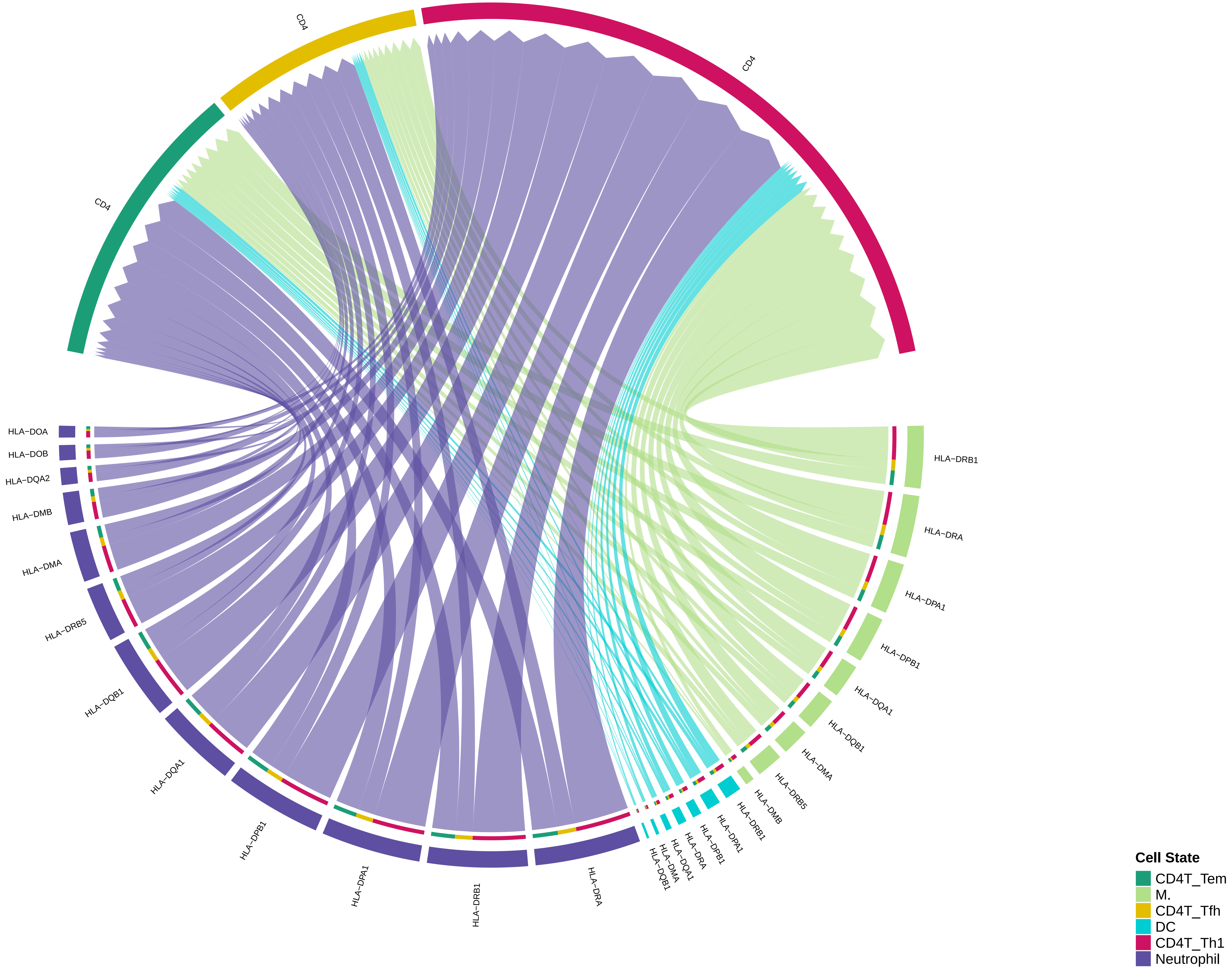

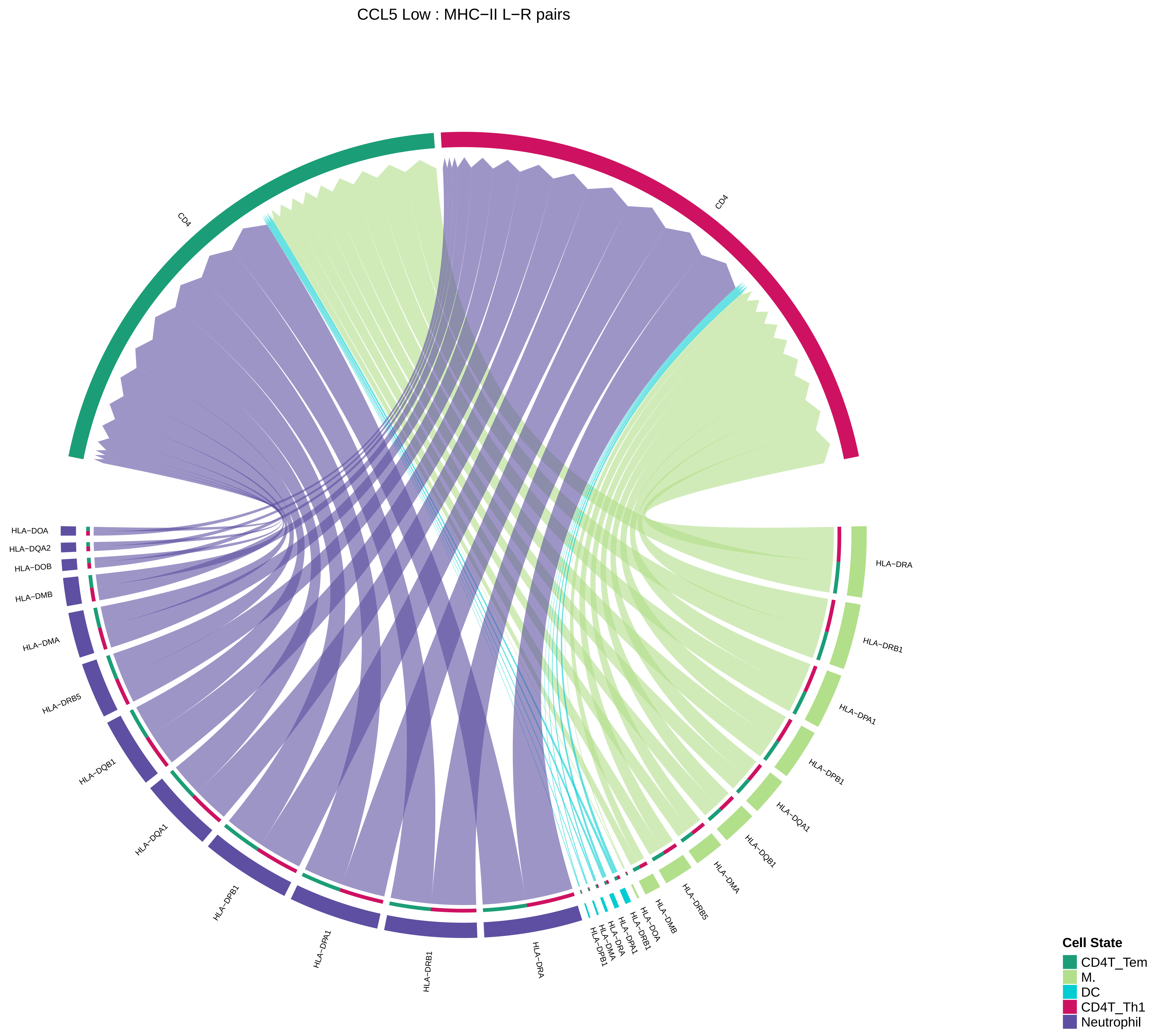

CCL5 Low : MHC−II L−R pairs
CD4
CD4
HLA−DOA
HLA−DQA2
HLA−DOB
HLA−DMB
HLA−DMA
HLA−DRB5
HLA−DQB1
HLA−DQA1
HLA−DPB1
HLA−DPA1
HLA−DRB1
HLA−DRA
HLA−DPB1
HLA−DMA
HLA−DRA
HLA−DPA1
HLA−DRB1
HLA−DOA
HLA−DMB
HLA−DRB5
HLA−DMA
HLA−DQB1
HLA−DQA1
HLA−DPB1
HLA−DPA1
HLA−DRB1
HLA−DRA
Cell State
CD4T_Tem
M.
DC
CD4T_Th1
Neutrophil

Cell Communication Analysis: Myeloid to Helper_T

Pathway: MIF

CCL5 High vs Low Expression Group Comparison

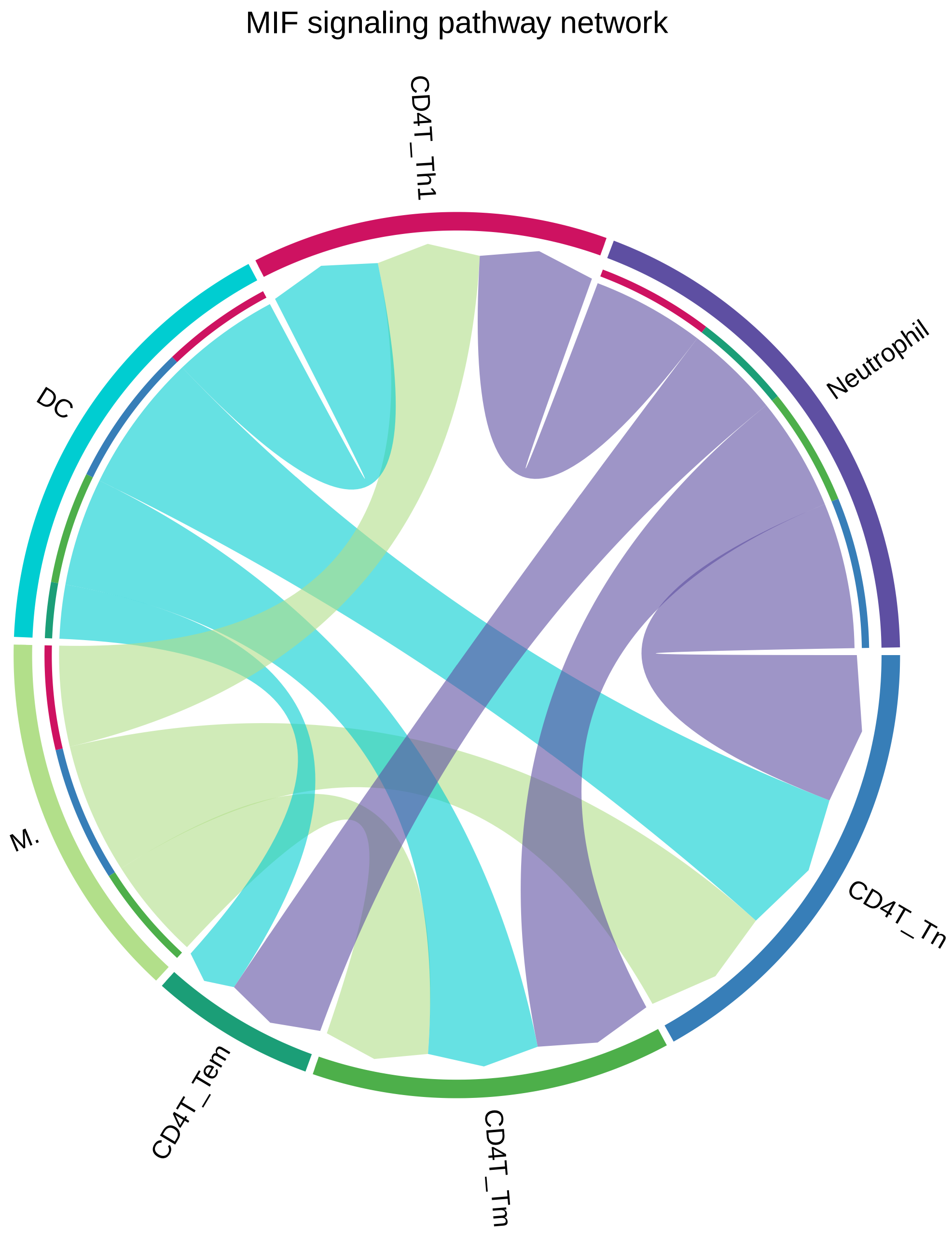

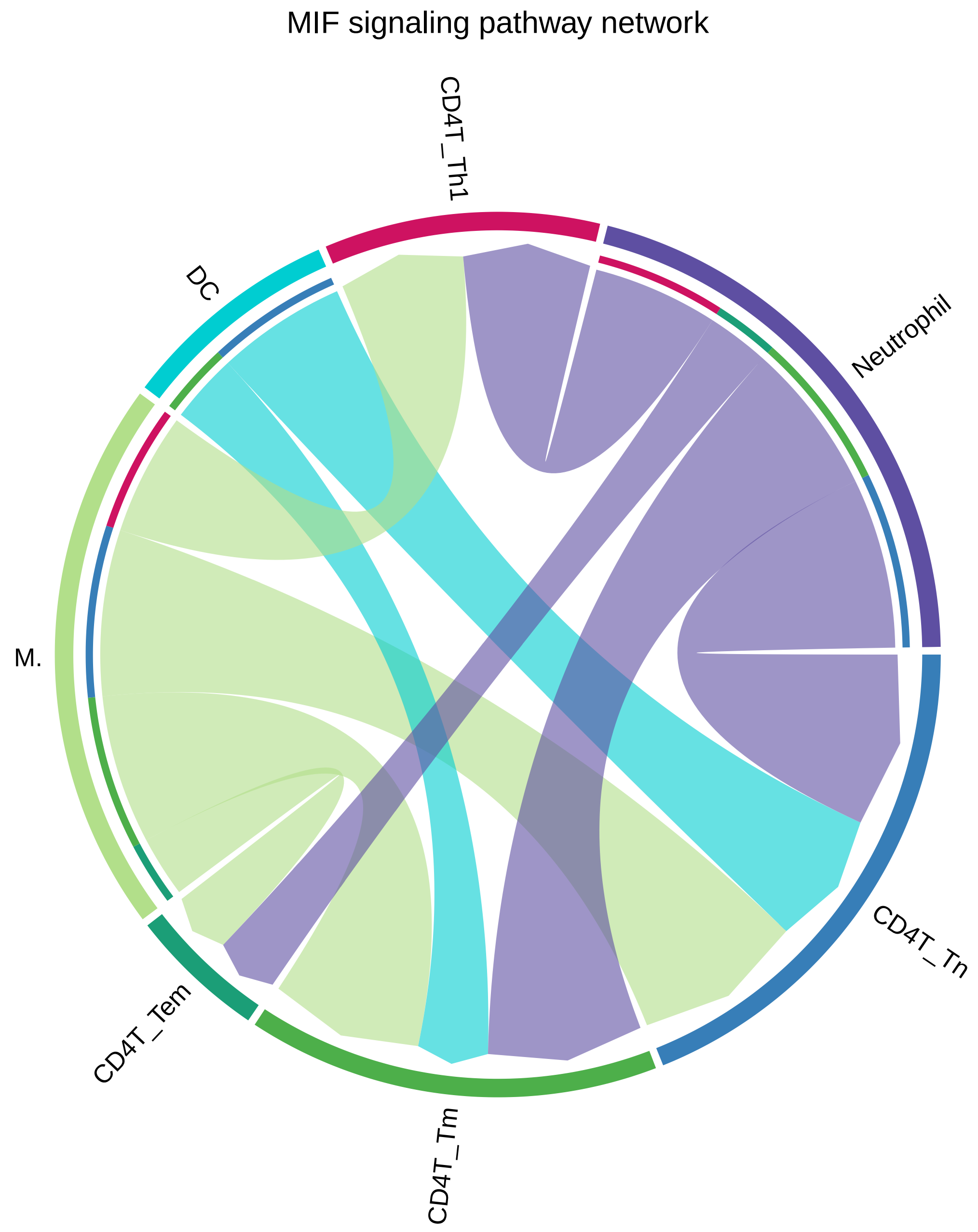

'3

4

CCL5 High : MIF L−R pairs

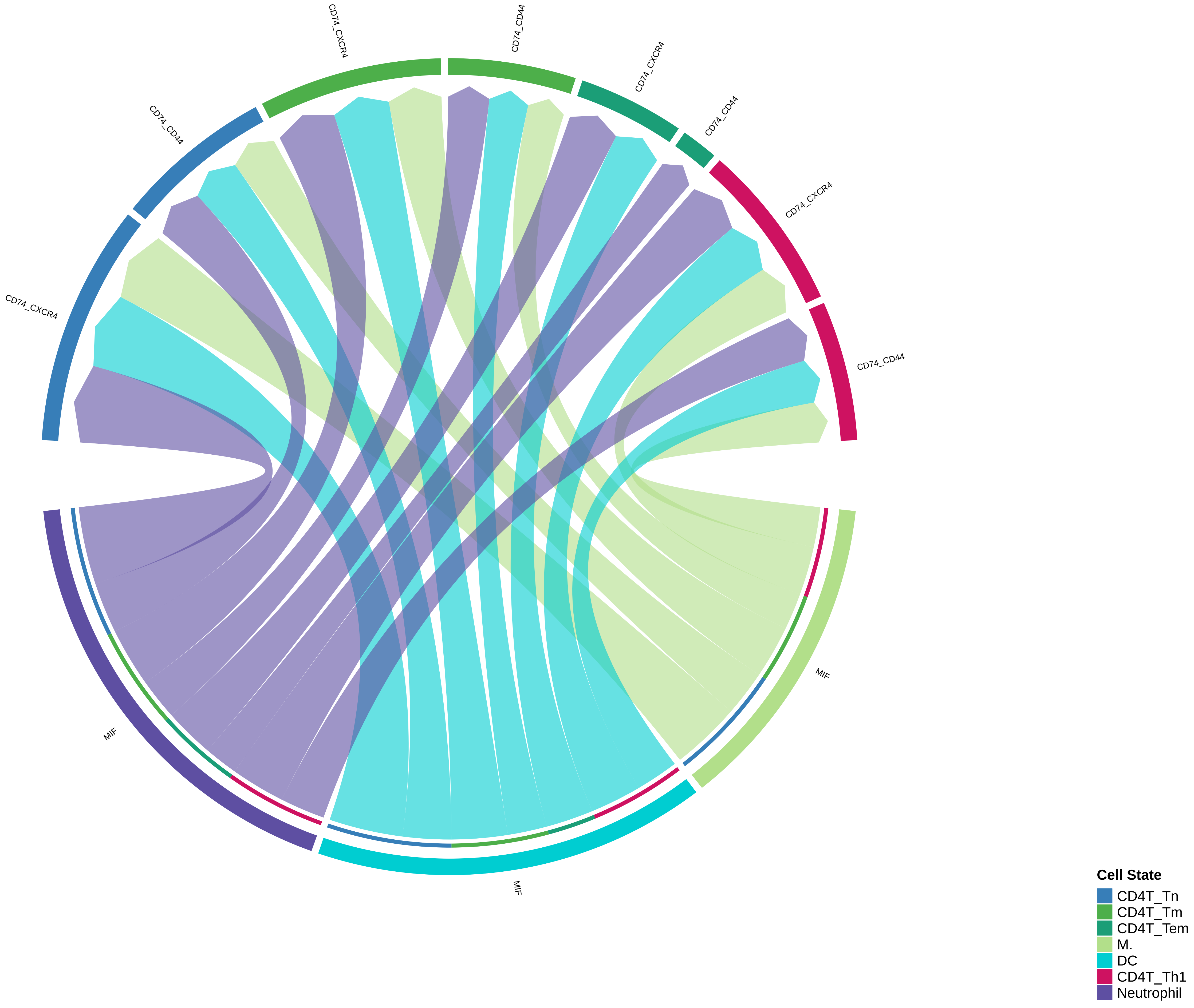

CCL5 Low : MIF L−R pairs

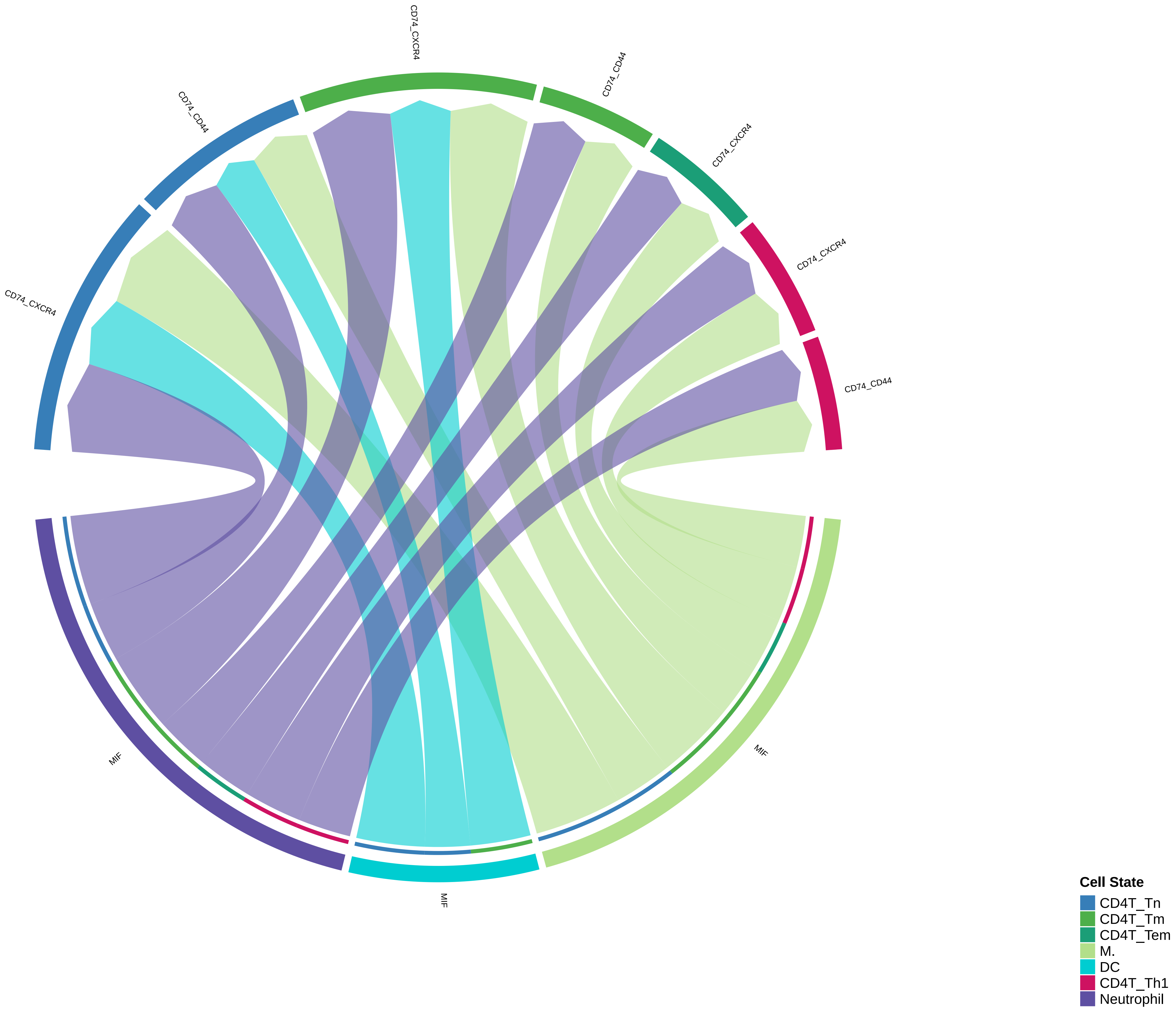

Cell Communication Analysis: Myeloid to Helper_T

Pathway: CLEC

CCL5 High vs Low Expression Group Comparison

CLEC signaling pathway network

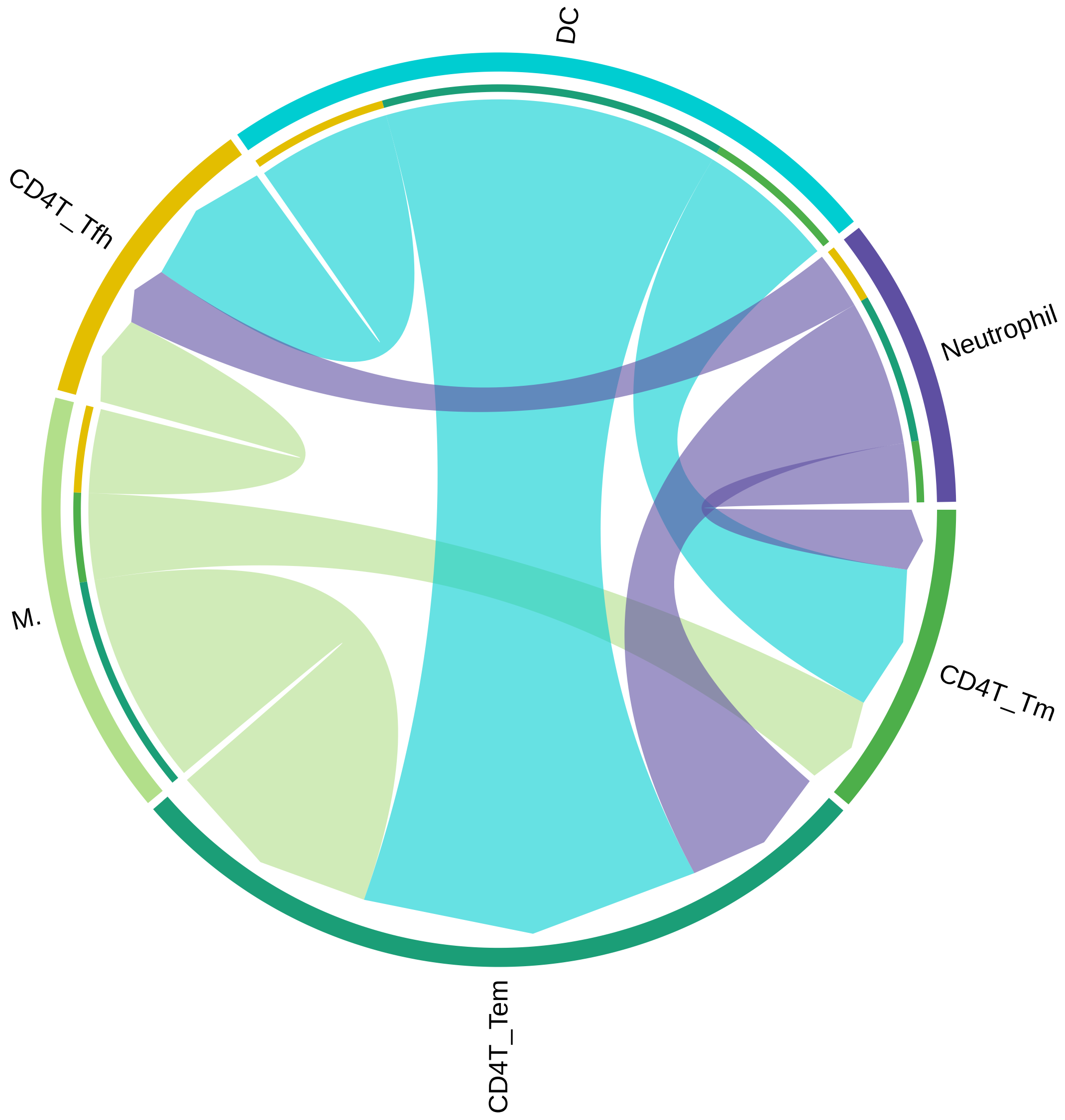

CLEC signaling pathway network

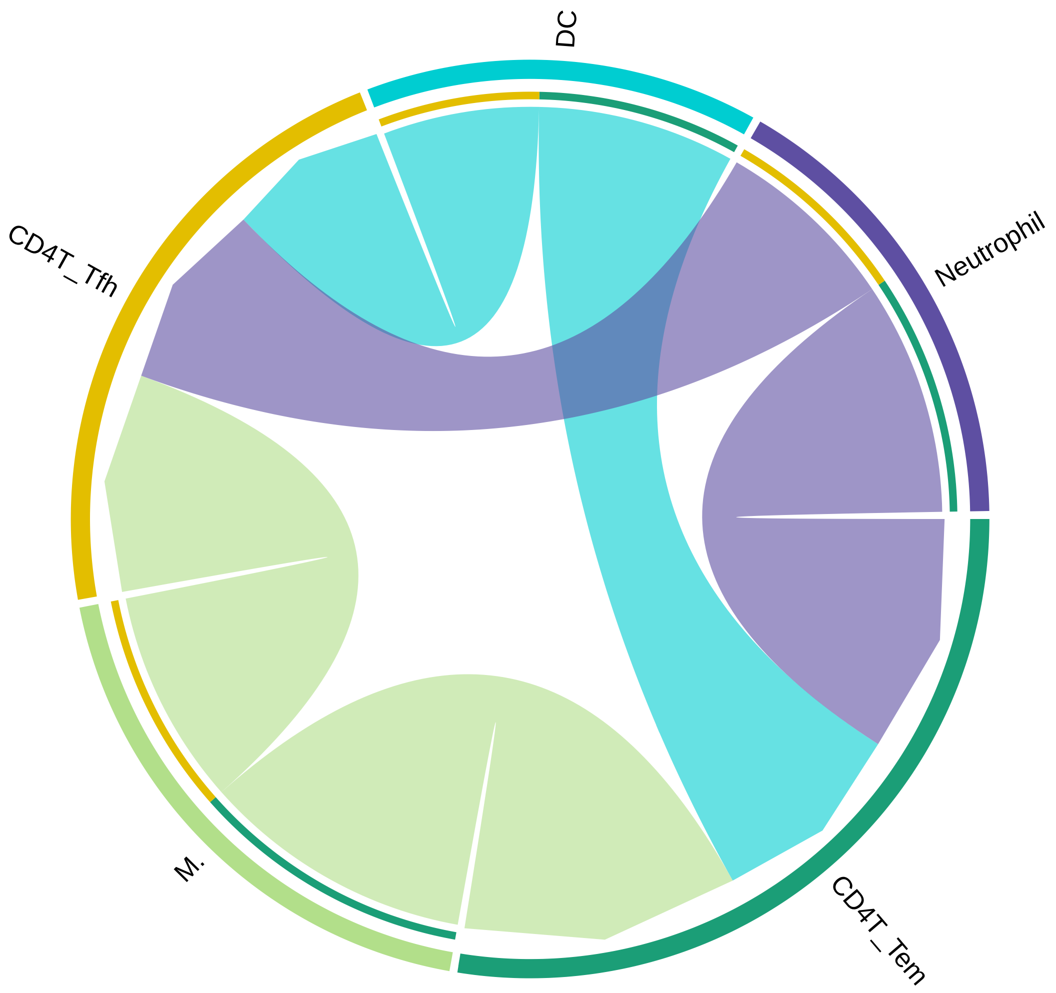

05

39

CCL5 High : CLEC L−R pairs

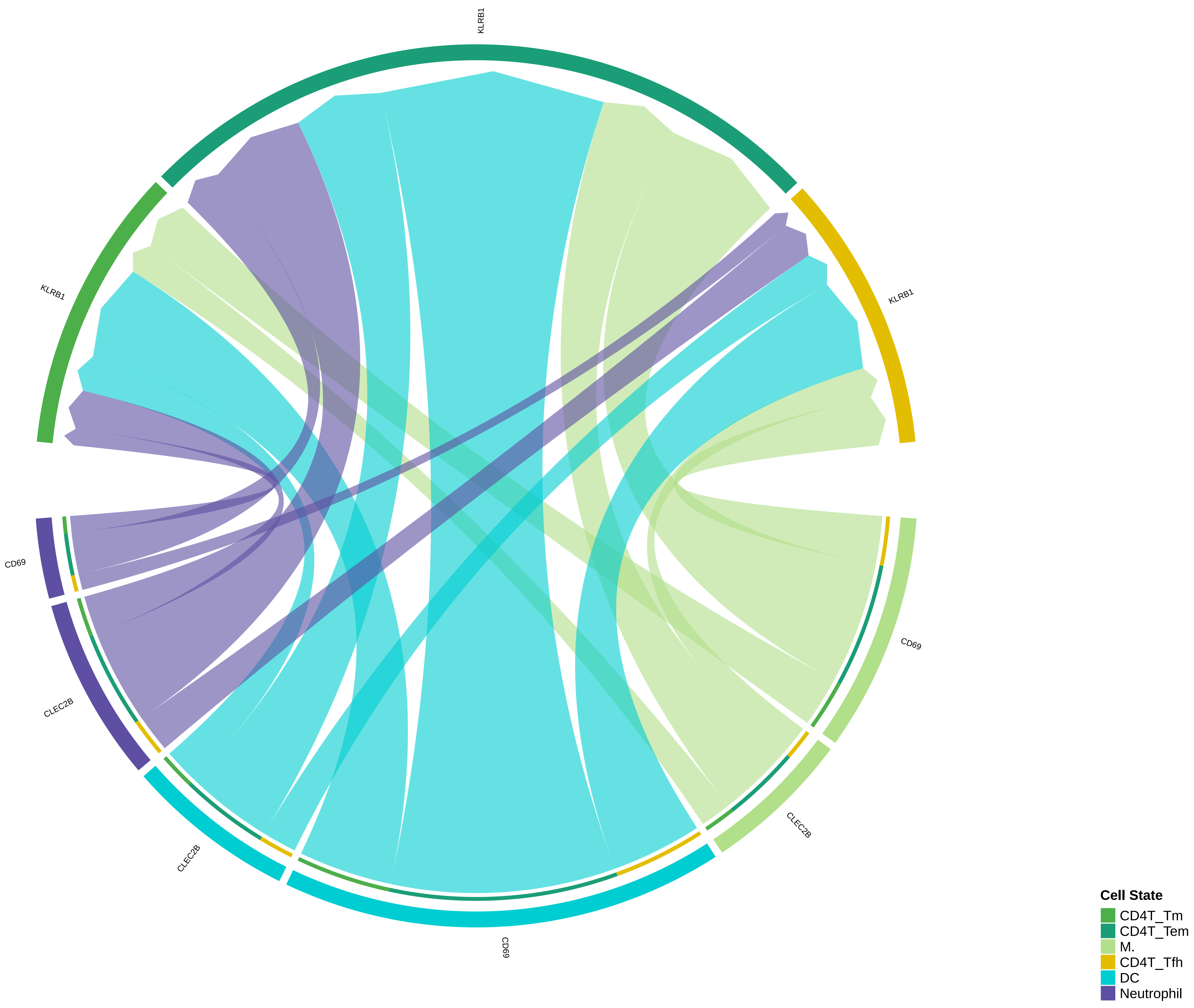

CCL5 Low : CLEC L−R pairs

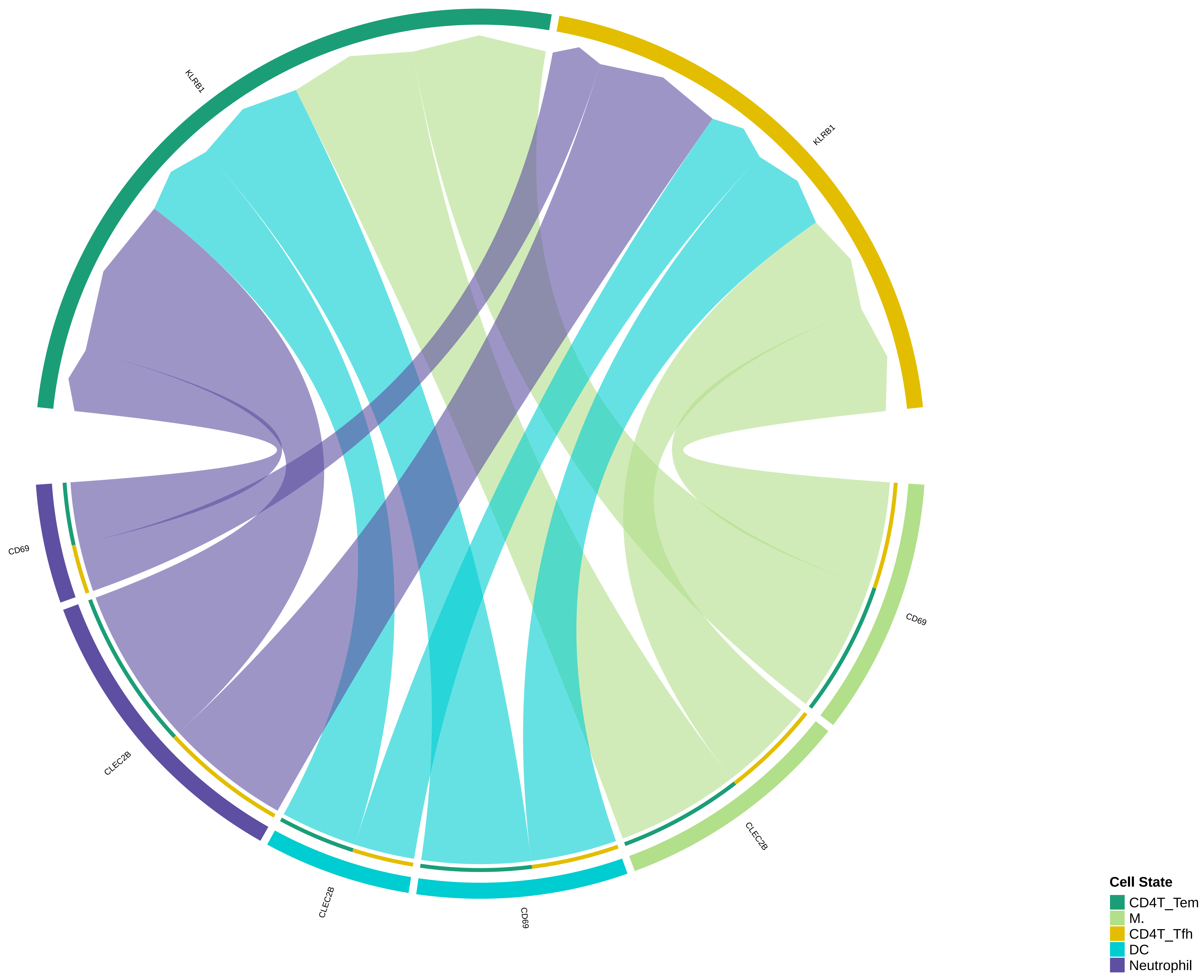

Cell Communication Analysis: Myeloid to Helper_T

Pathway: MIF

CCL5 High vs Low Expression Group Comparison

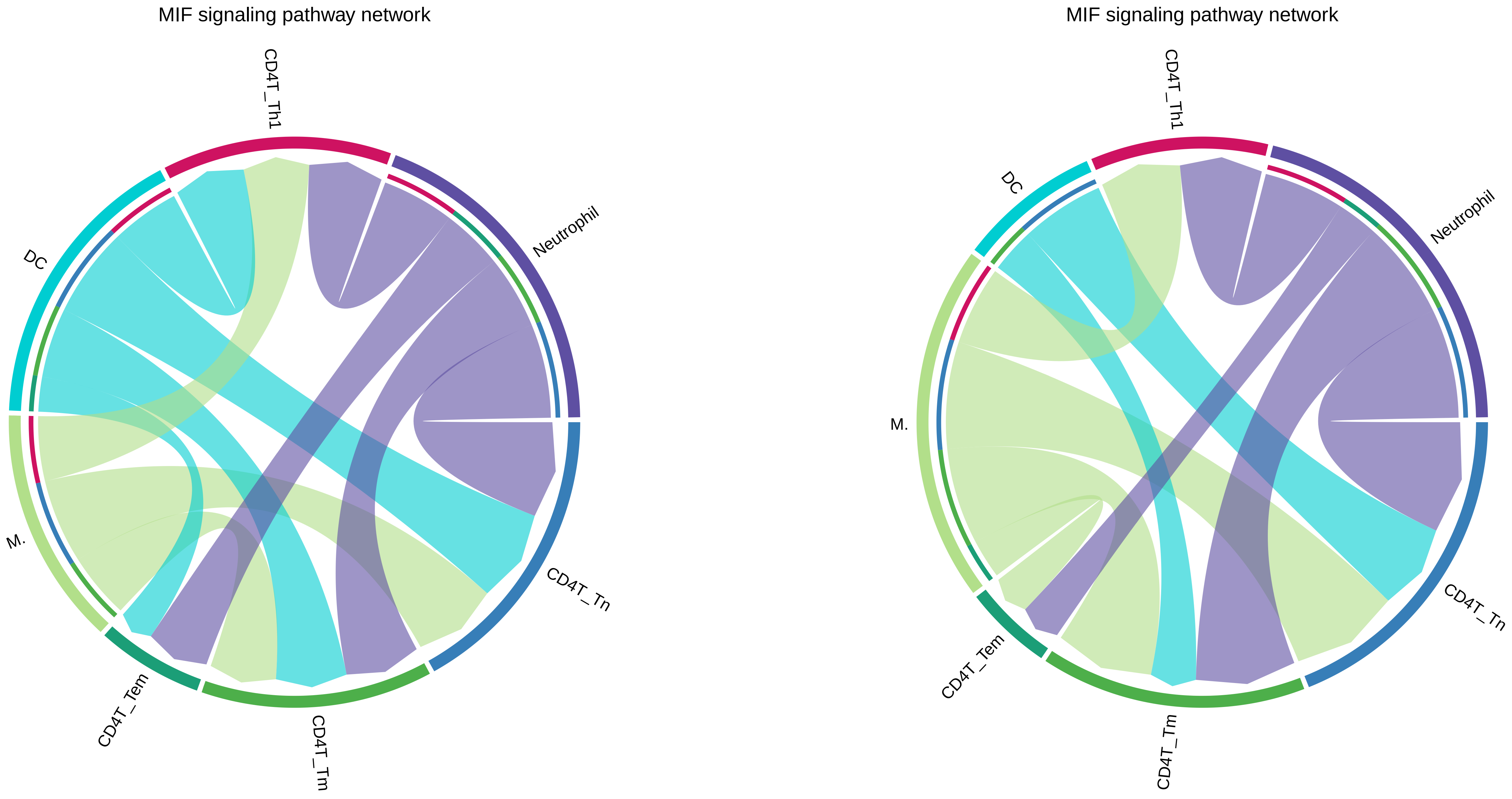

'3 54

CCL5 High : MIF L−R pairs

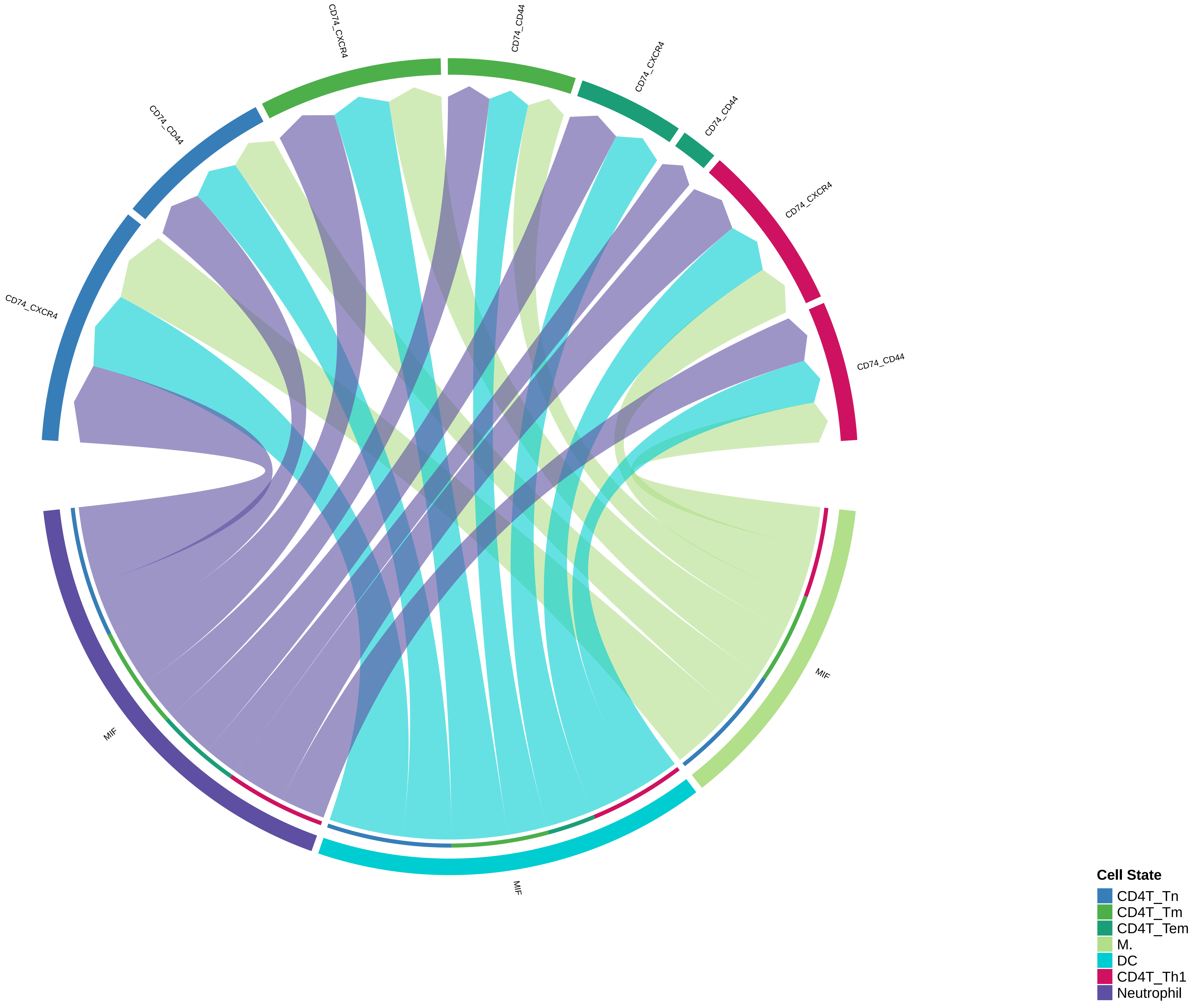

CCL5 Low : MIF L−R pairs

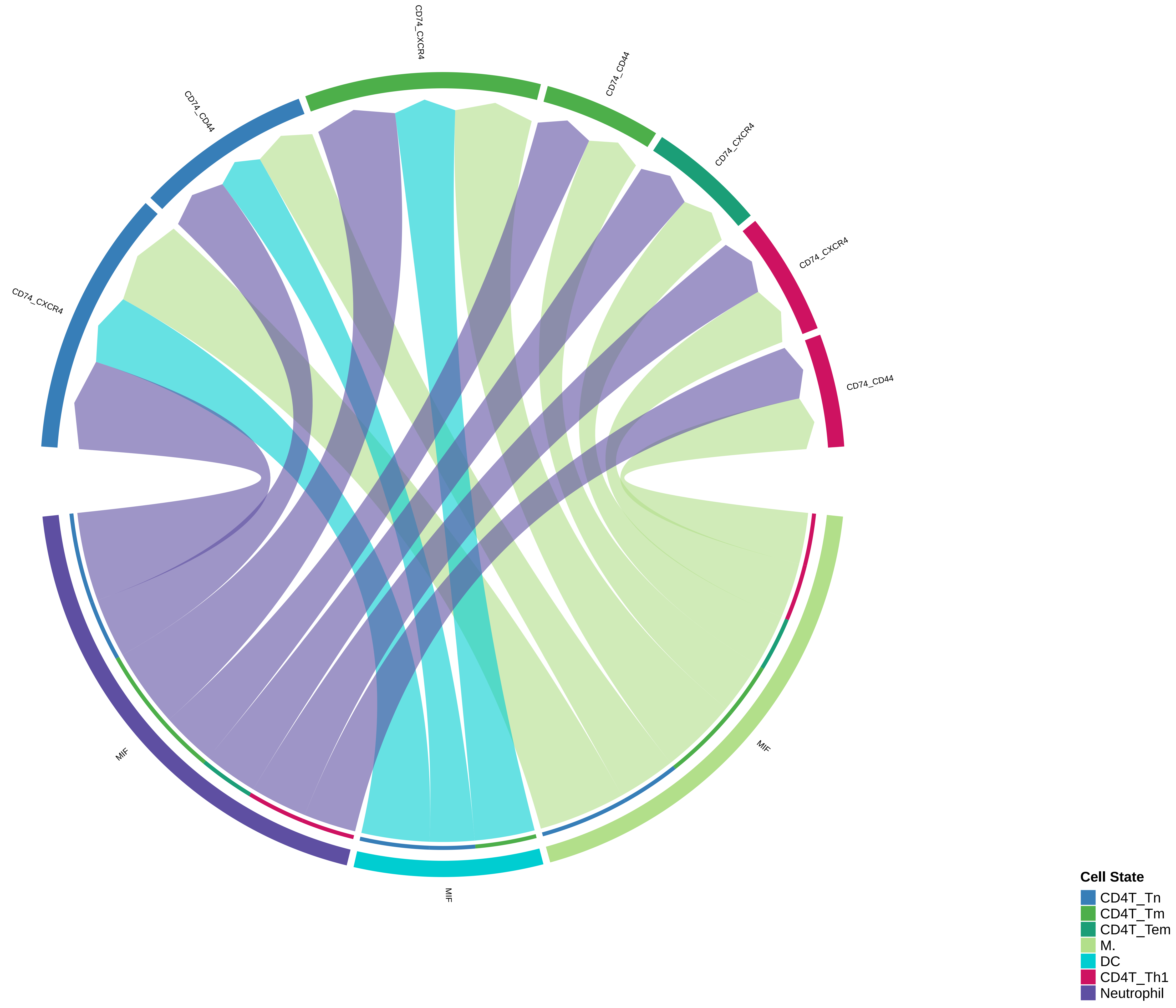

Cell Communication Analysis: Myeloid to Cytotoxic_T

Pathway: MHC−I

CCL5 High vs Low Expression Group Comparison

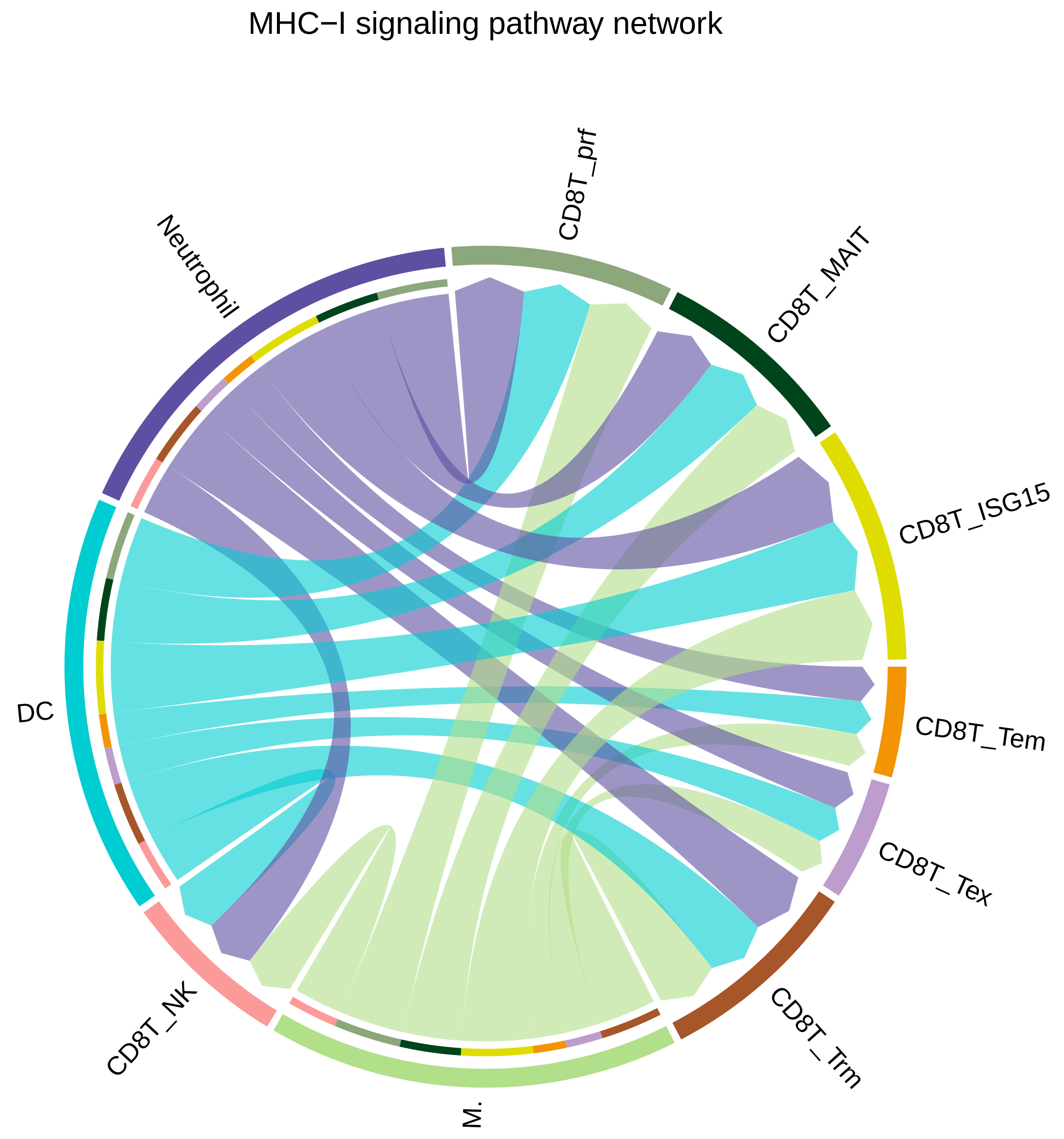

09

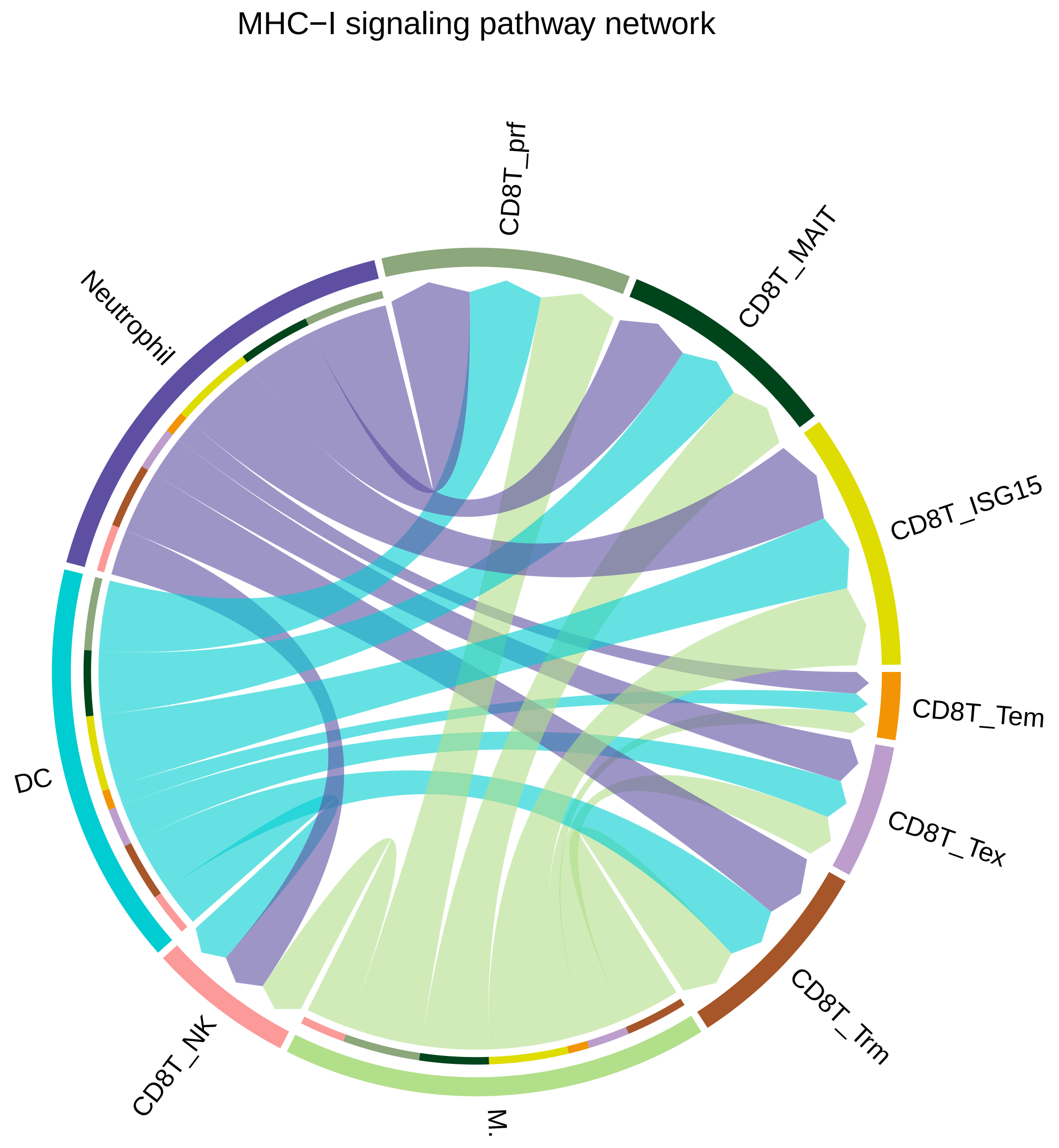

81

CCL5 High : MHC−I L−R pairs

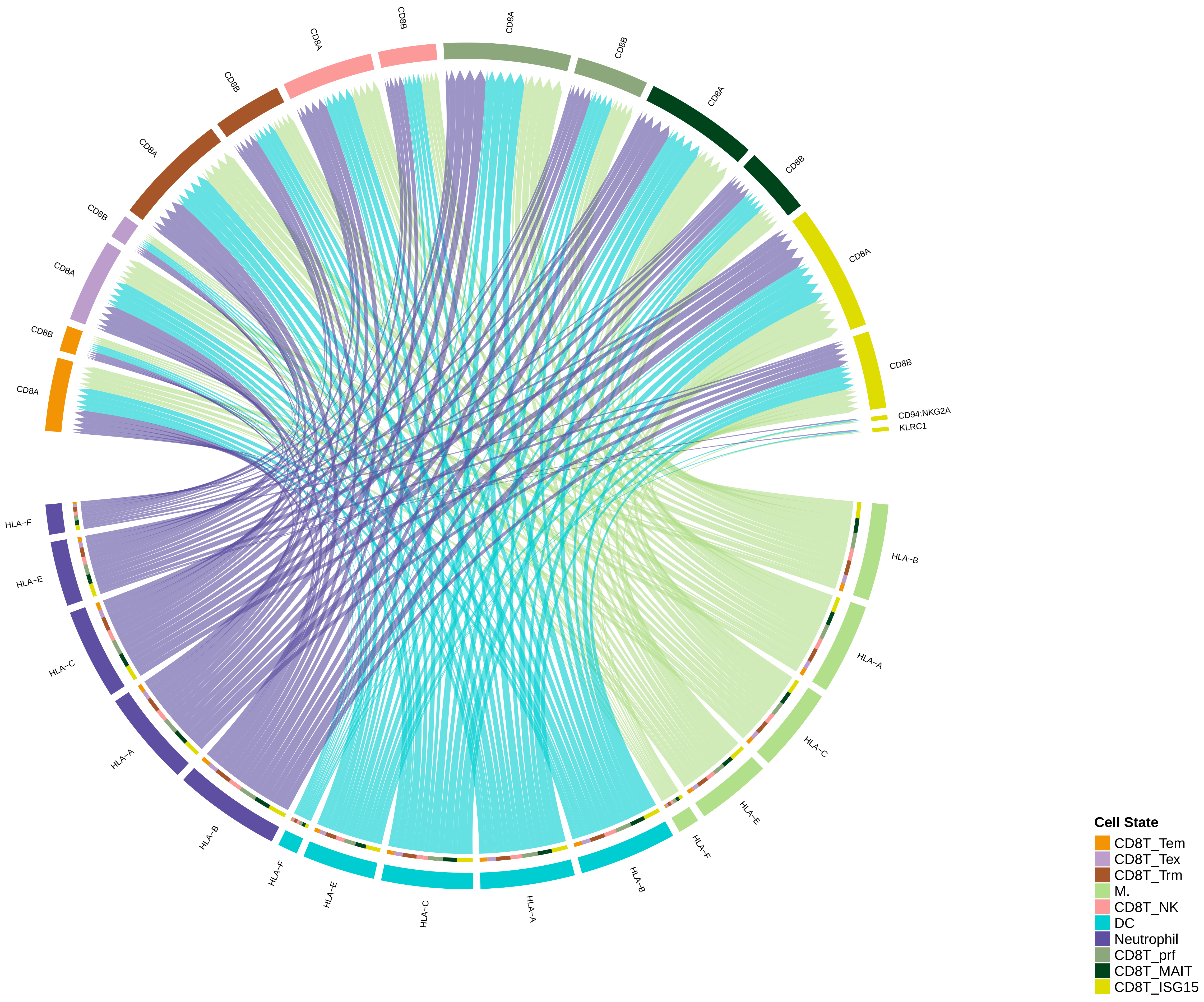

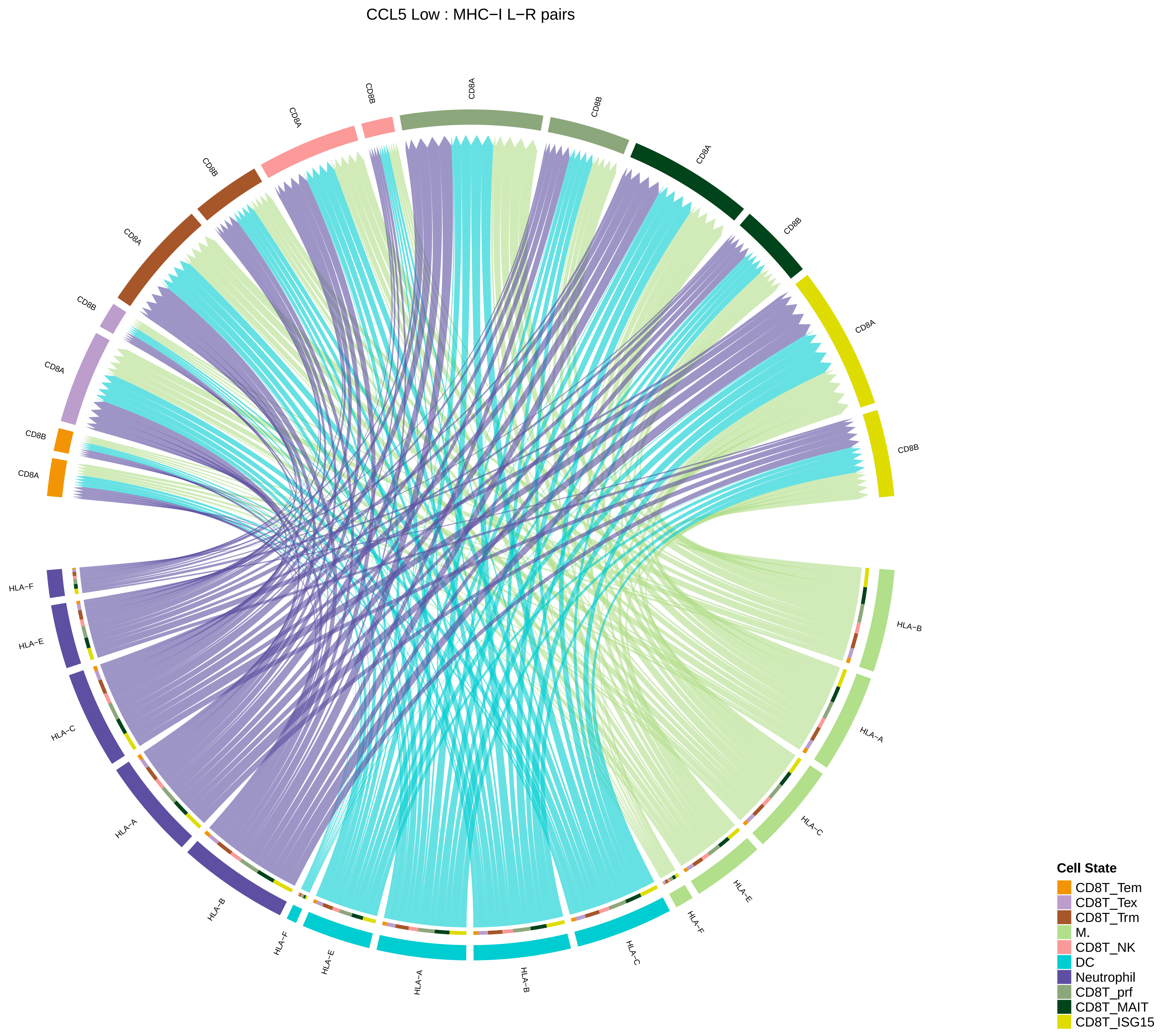
CCL5 Low : MHC−I L−R pairs
CD8A
CD8B
HLA−F
HLA−E
HLA−C
HLA−A
HLA−B
Cell State
CD8T_Tem
CD8T_Tex
CD8T_Trm
M.
CD8T_NK
DC
Neutrophil
CD8T_prf
CD8T_MAIT
CD8T_ISG15

Cell Communication Analysis: Myeloid to Cytotoxic_T

Pathway: MIF

CCL5 High vs Low Expression Group Comparison

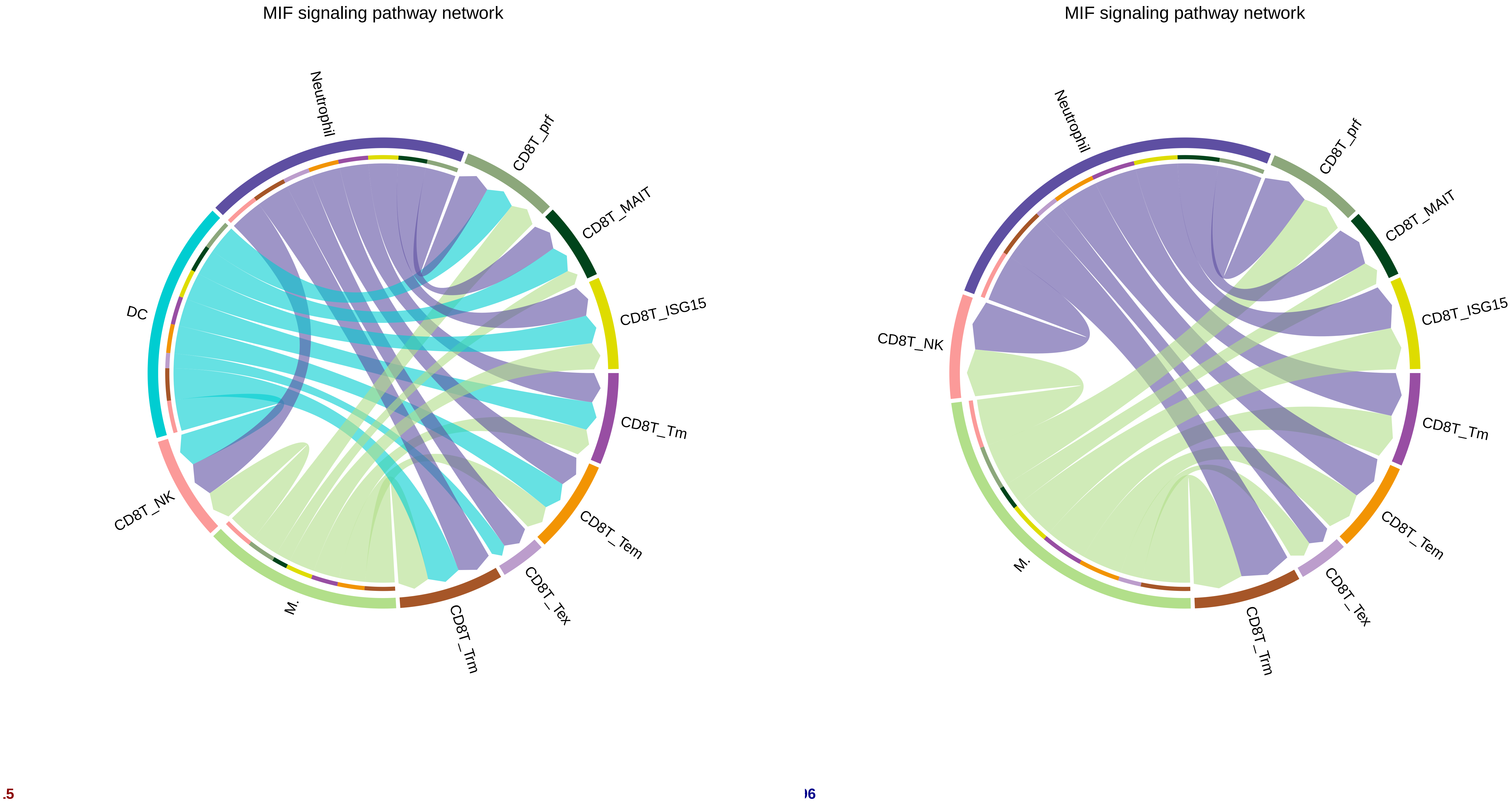

CCL5 High : MIF L−R pairs

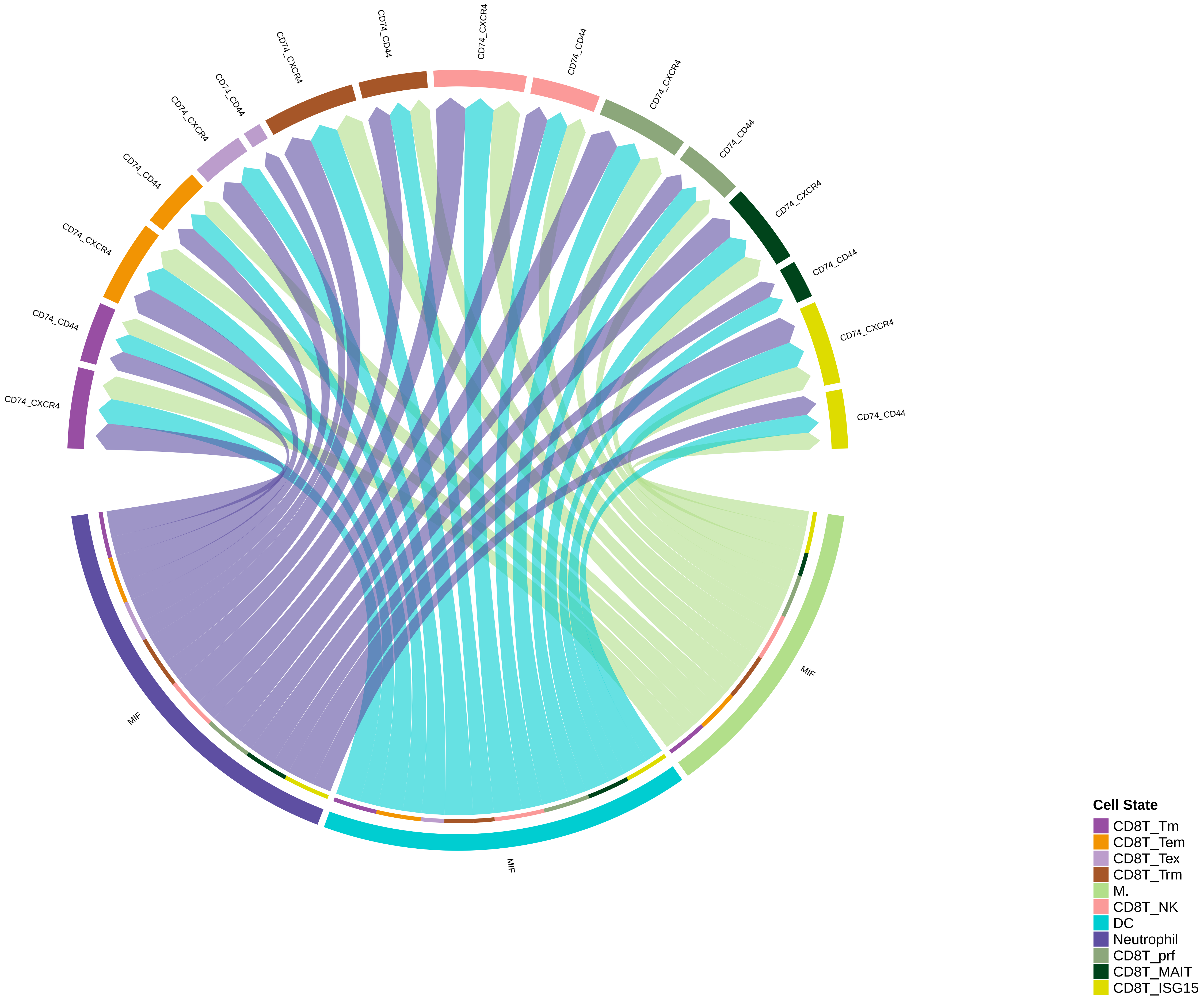

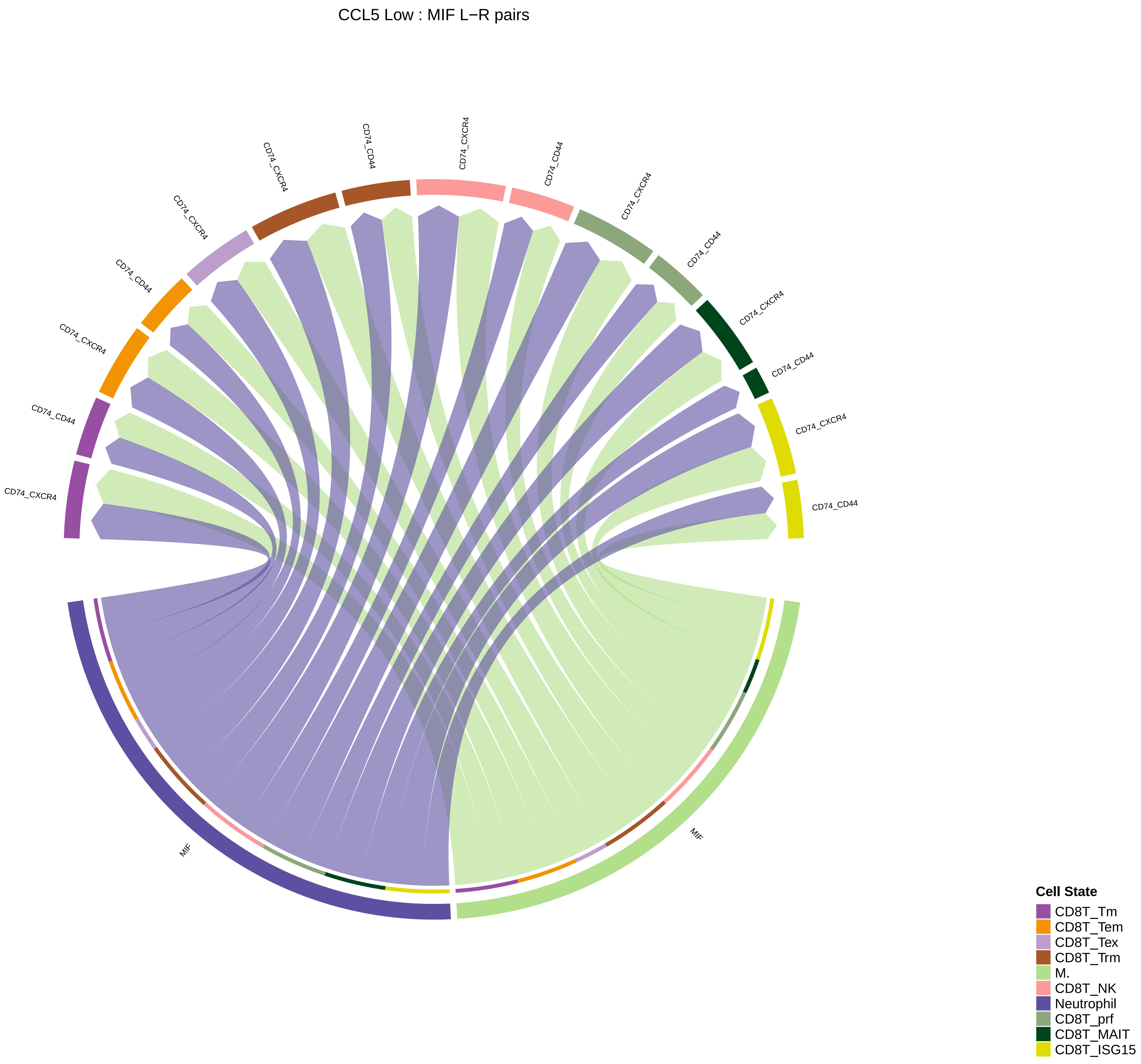
CCL5 Low : MIF L−R pairs
CD74_CXCR4
CD74_CD44
CD74_CXCR4
CD74_CD44
CD74_CXCR4
CD74_CXCR4
CD74_CD44
CD74_CXCR4
CD74_CD44
CD74_CXCR4
CD74_CD44
CD74_CXCR4
CD74_CD44
CD74_CXCR4
CD74_CD44
MIF
MIF
Cell State
CD8T_Tm
CD8T_Tem
CD8T_Tex
CD8T_Trm
M.
CD8T_NK
Neutrophil
CD8T_prf
CD8T_MAIT
CD8T_ISG15

Cell Communication Analysis: Myeloid to Cytotoxic_T

Pathway: MIF

CCL5 High vs Low Expression Group Comparison

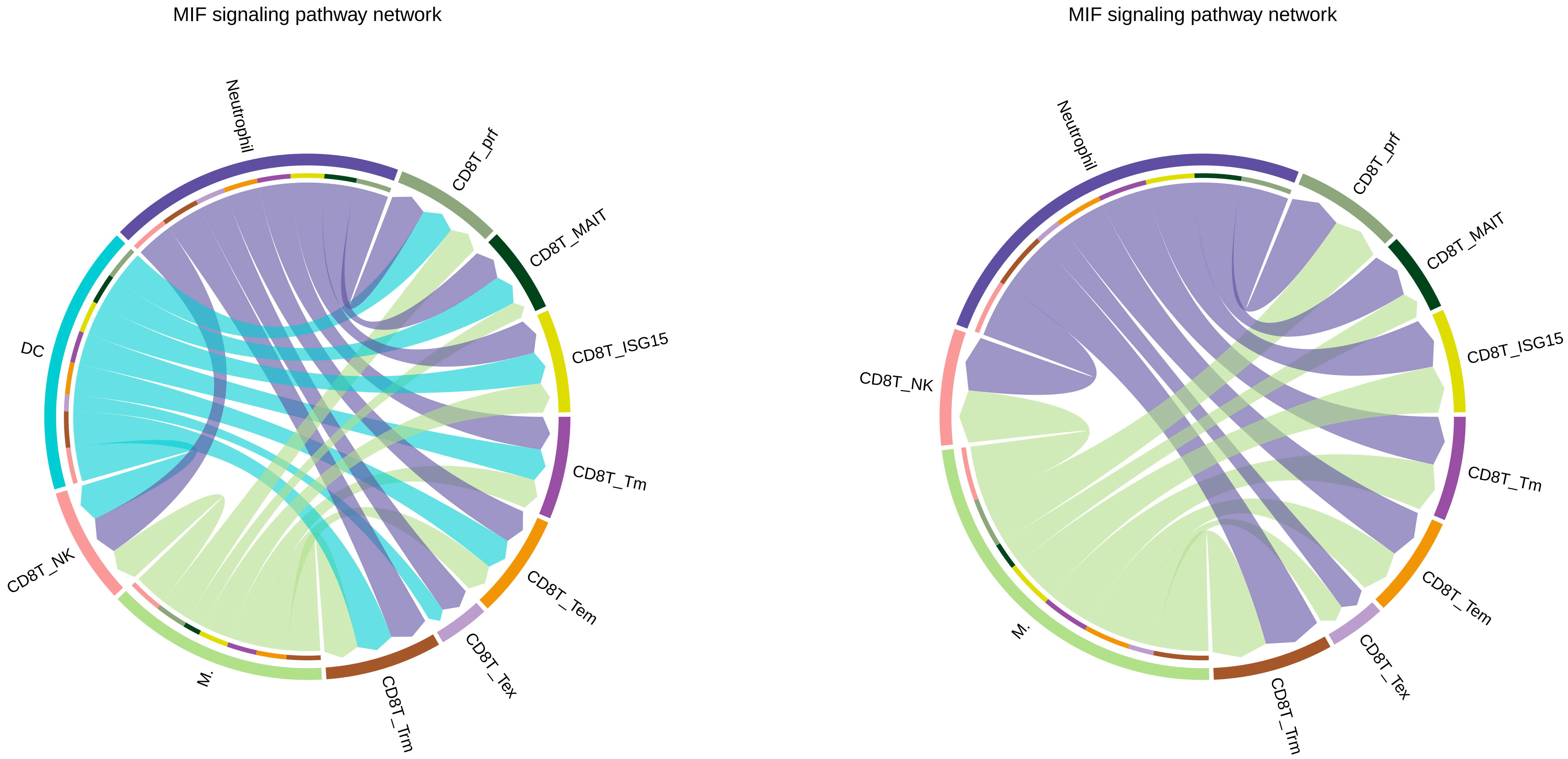

.5 06

CCL5 High : MIF L−R pairs

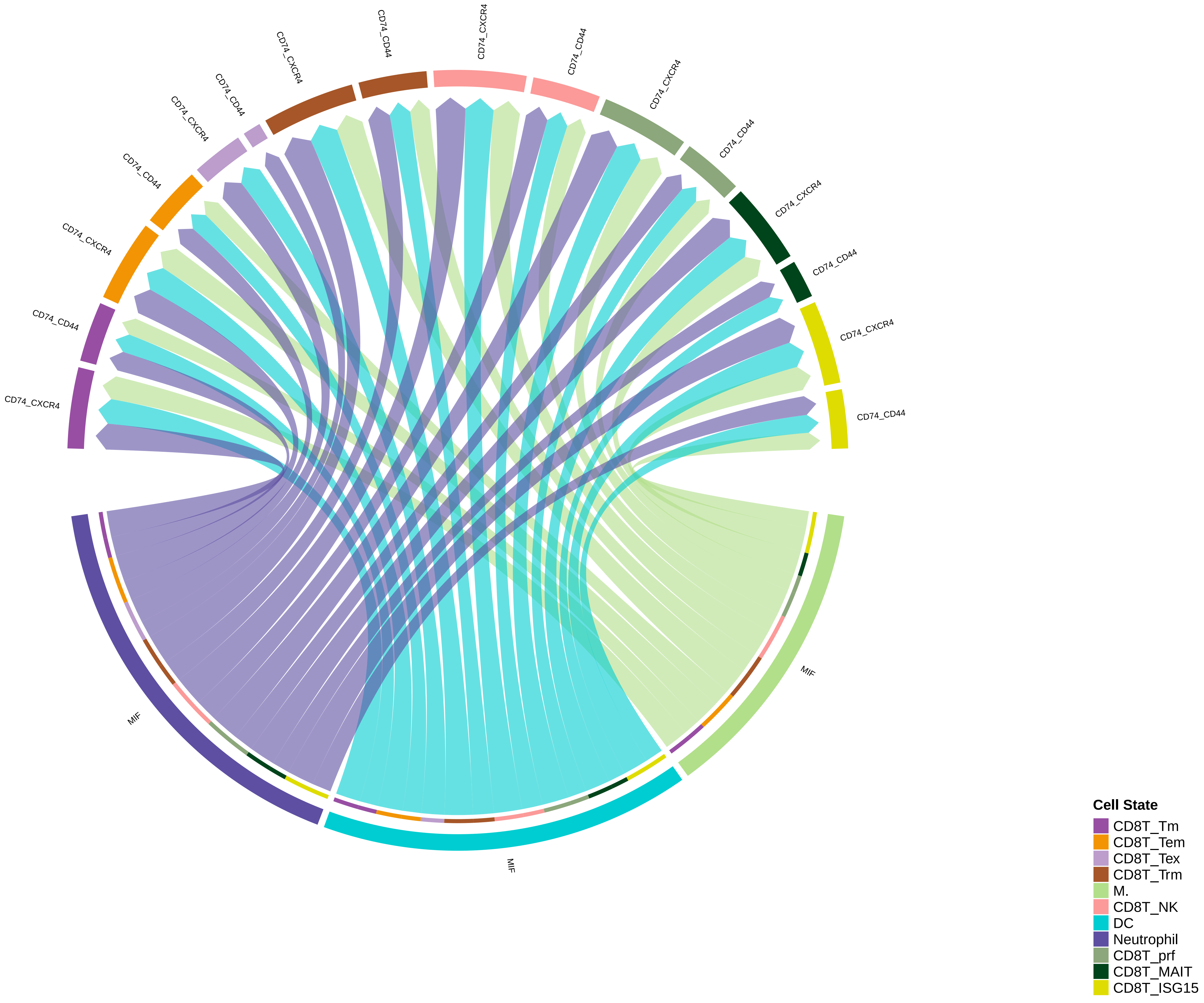

CCL5 Low : MIF L−R pairs

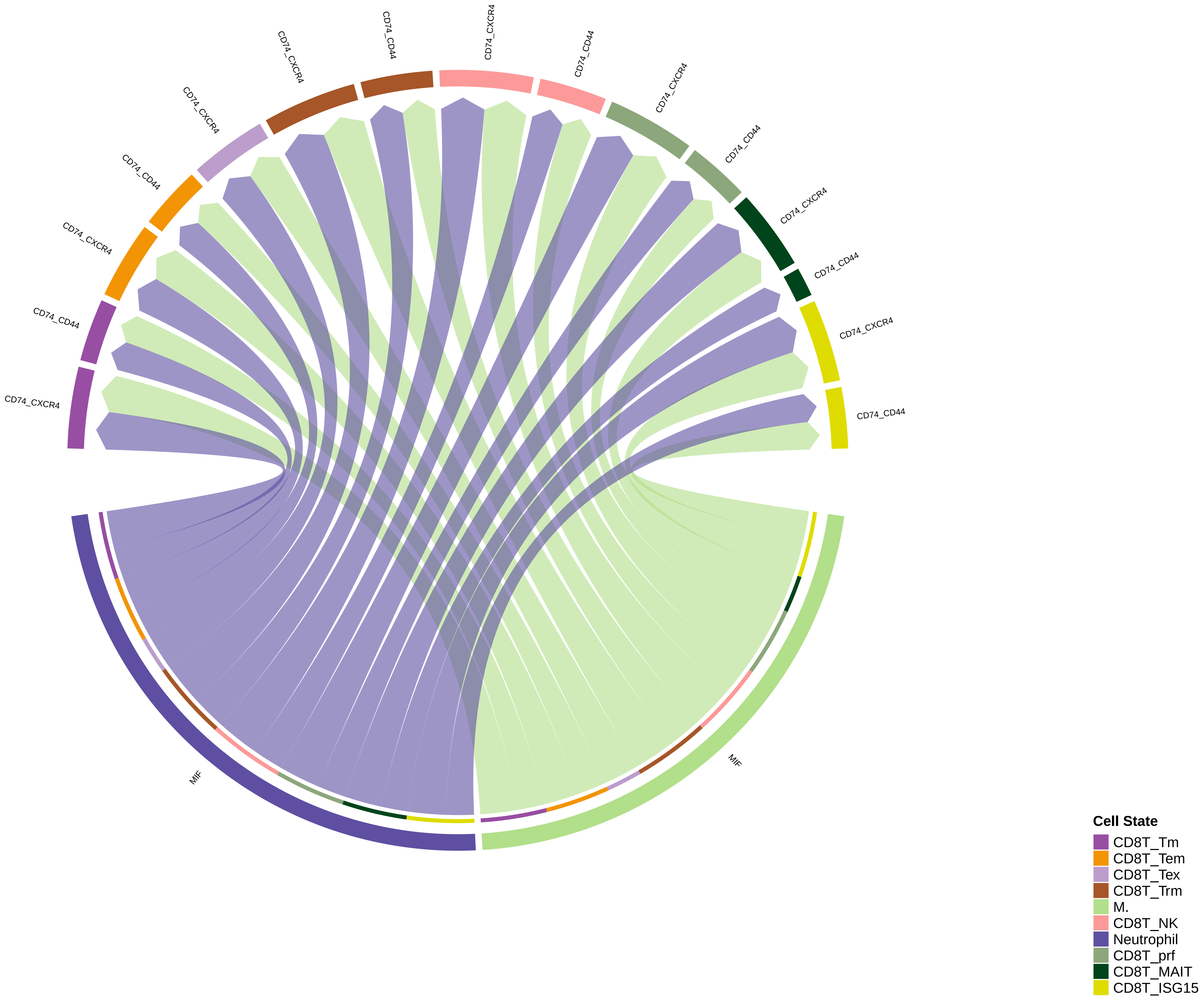

Cell Communication Analysis: Myeloid to Cytotoxic_T

Pathway: MHC−I

CCL5 High vs Low Expression Group Comparison

MHC−I signaling pathway network

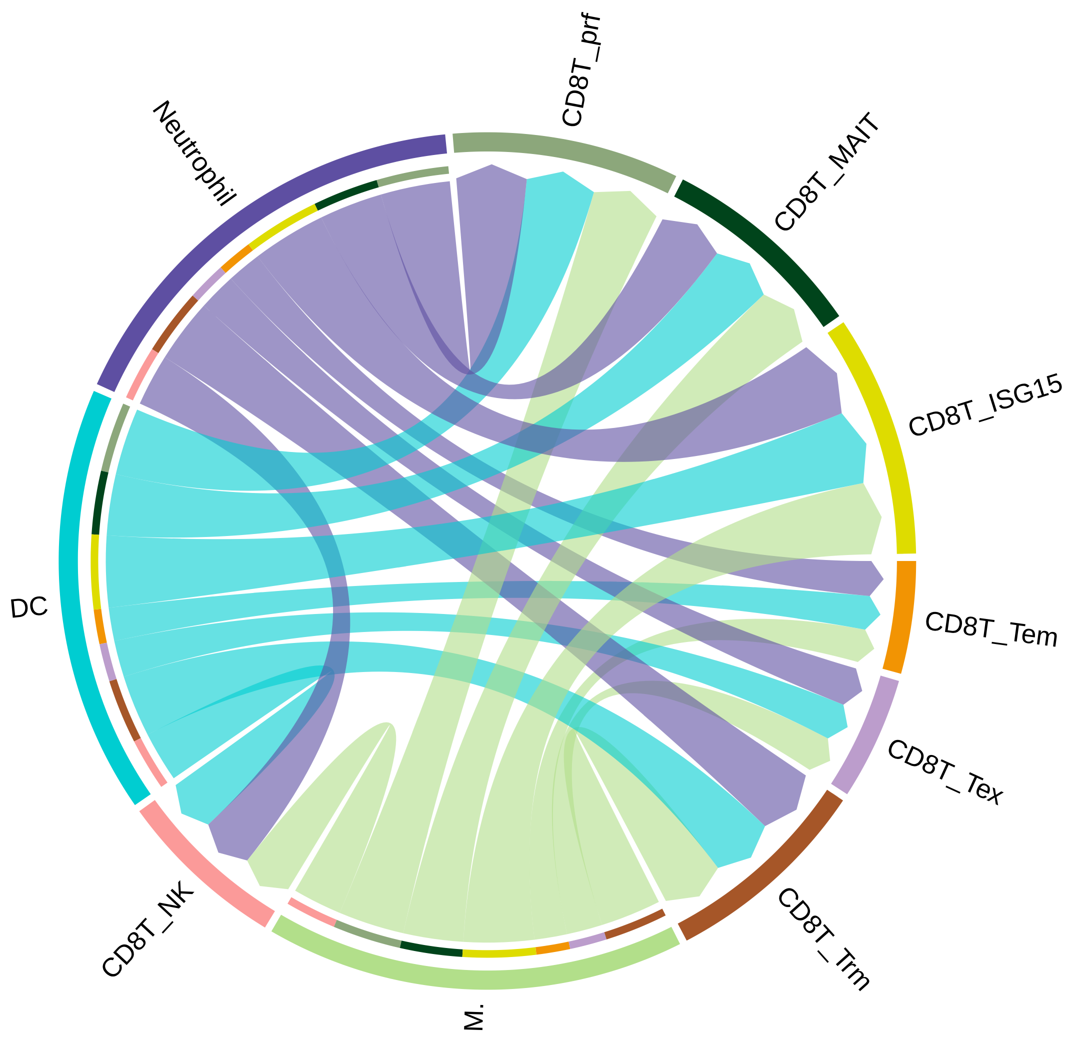

MHC−I signaling pathway network

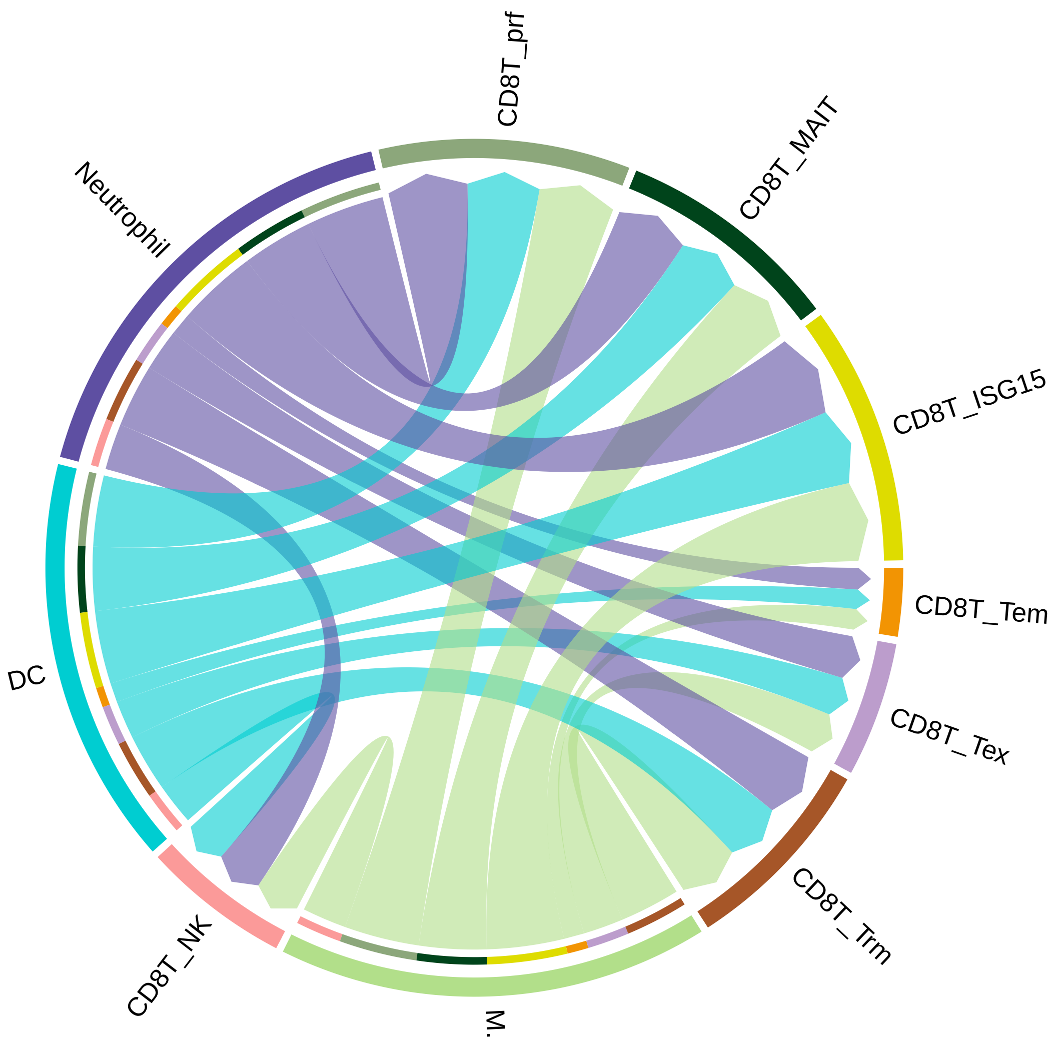

09

81

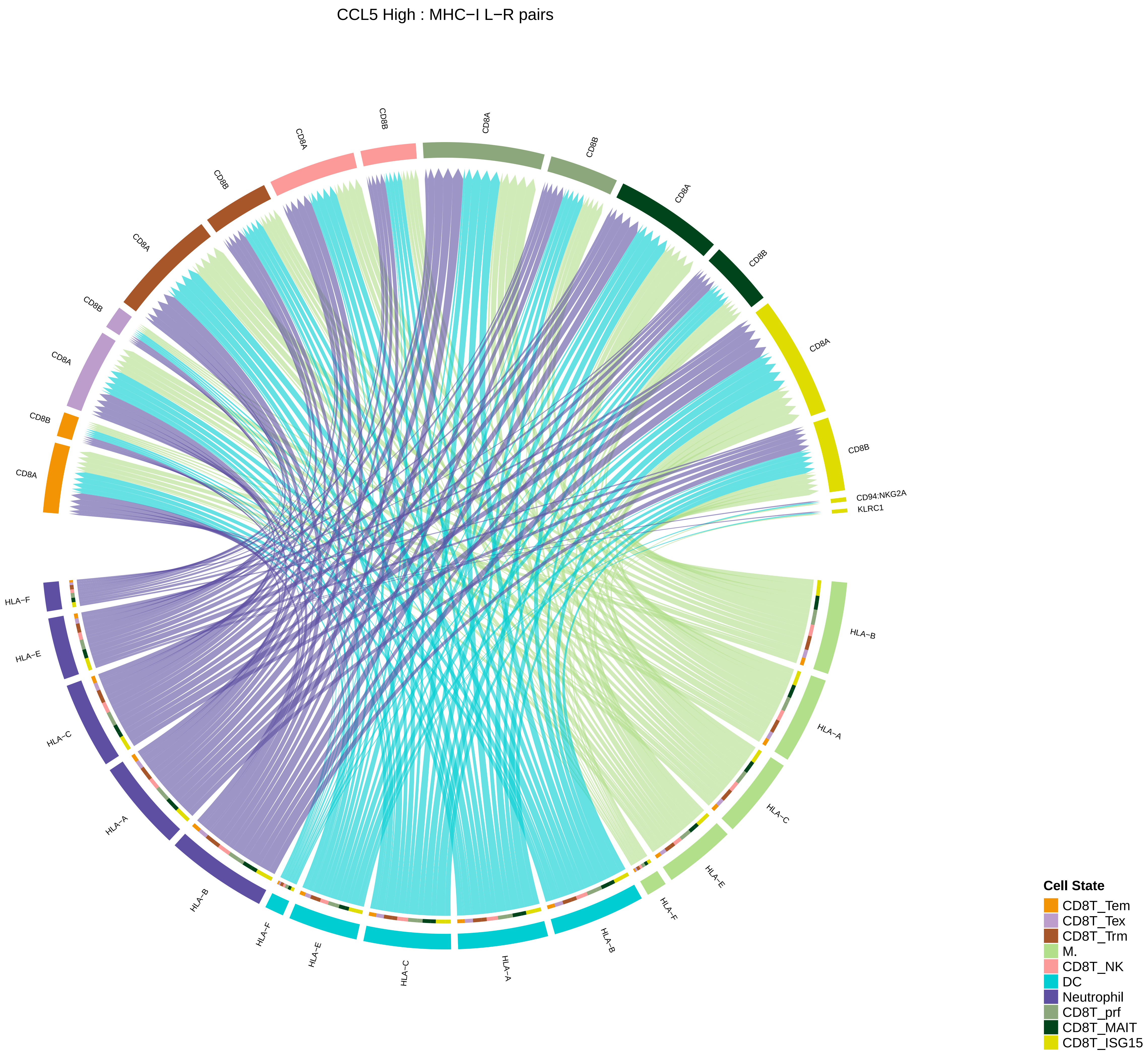

CCL5 High : MHC−I L−R pairs
CD8A
CD8B
CD8A
CD8B
CD8A
CD8B
CD8A
CD8B
CD8A
CD8B
CD8A
CD8B
CD8A
CD8B
CD94:NKG2A
KLRC1
HLA−B
HLA−A
HLA−C
HLA−E
HLA−F
HLA−B
HLA−A
HLA−C
HLA−E
HLA−F
HLA−B
HLA−A
HLA−C
HLA−E
HLA−F
Cell State
CD8T_Tem
CD8T_Tex
CD8T_Trm
M.
CD8T_NK
DC
Neutrophil
CD8T_prf
CD8T_MAIT
CD8T_ISG15

CCL5 Low : MHC−I L−R pairs

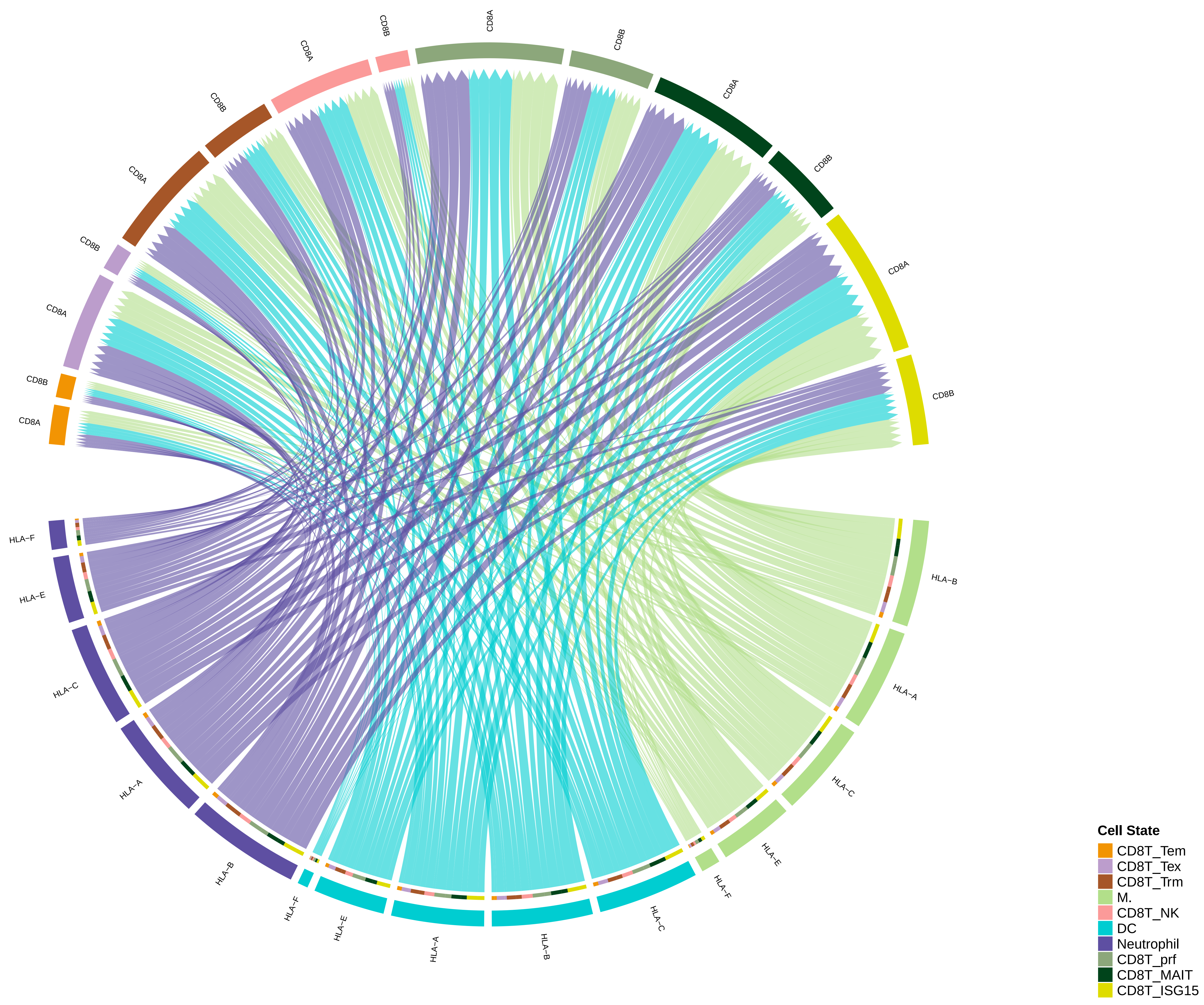

Cell Communication Analysis: Myeloid to Cytotoxic_T

Pathway: MHC−II

CCL5 High vs Low Expression Group Comparison

MHC−II signaling pathway network

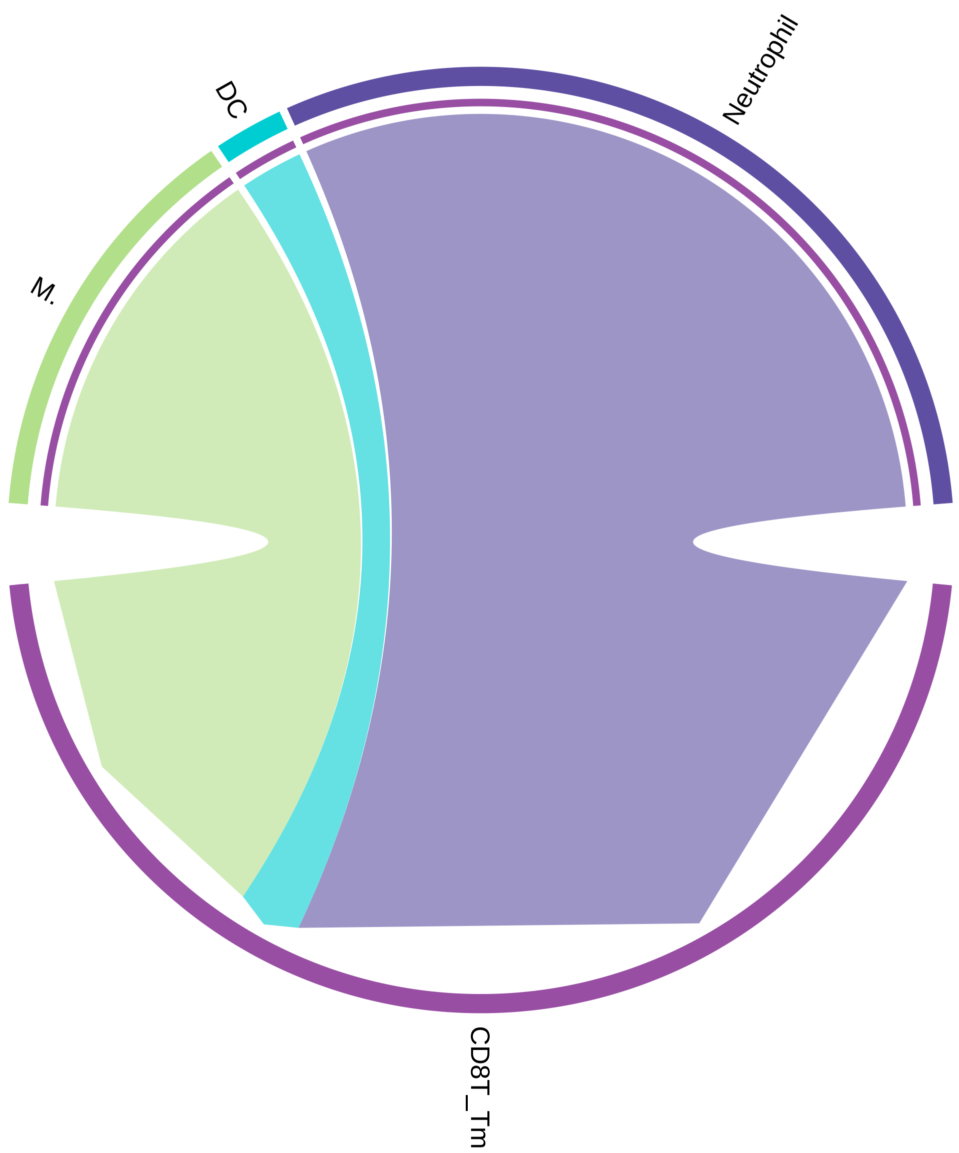

MHC−II signaling pathway network

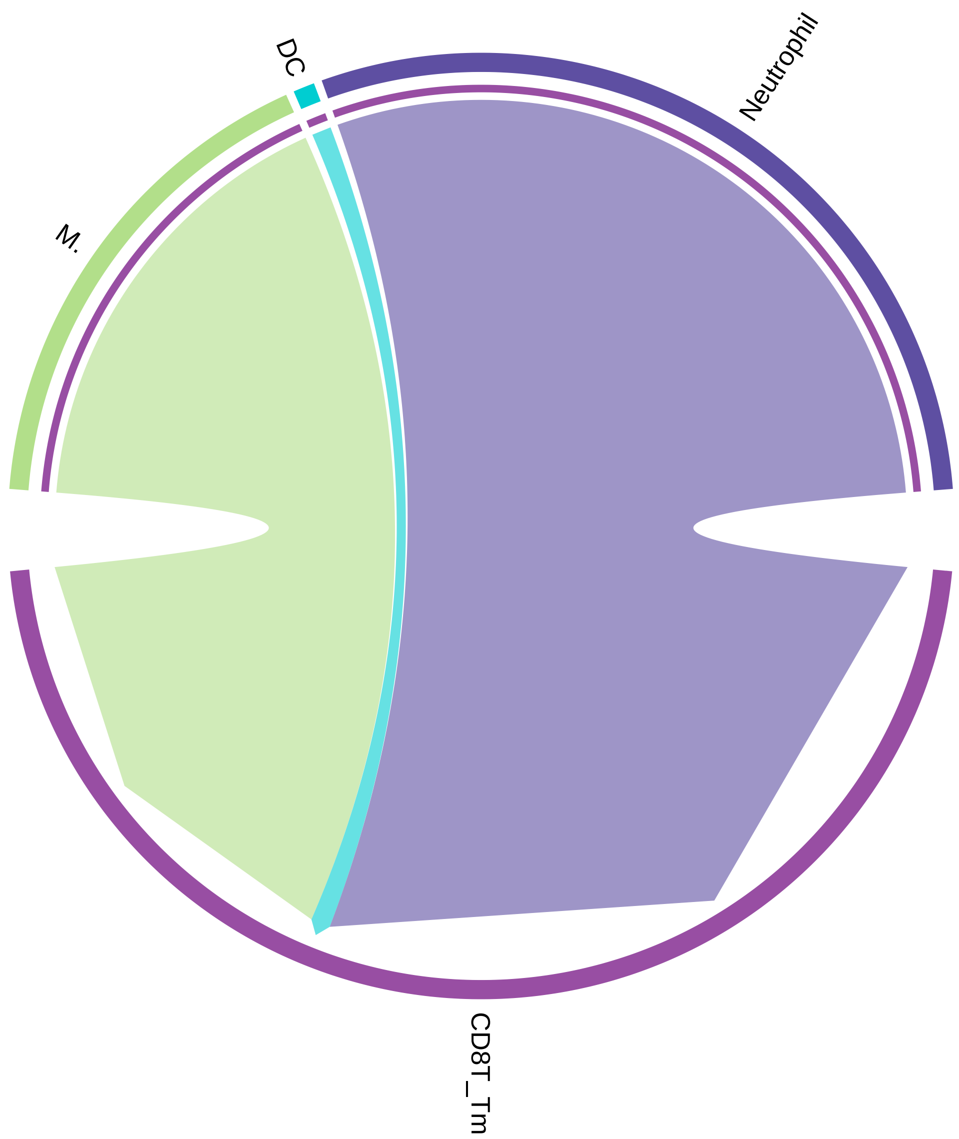

[illegible]4

[illegible]7

CCL5 High : MHC−II L−R pairs

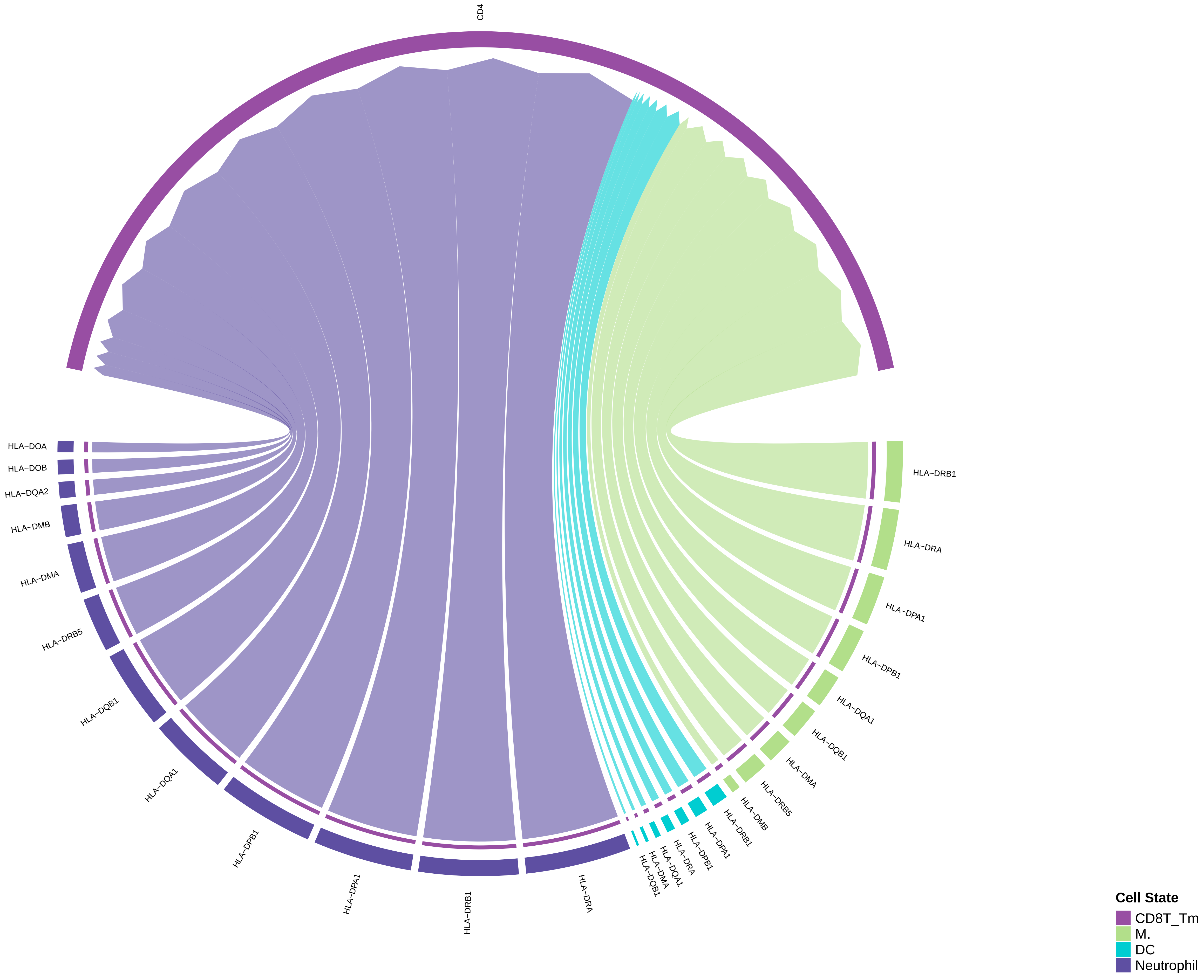

CCL5 Low : MHC−II L−R pairs

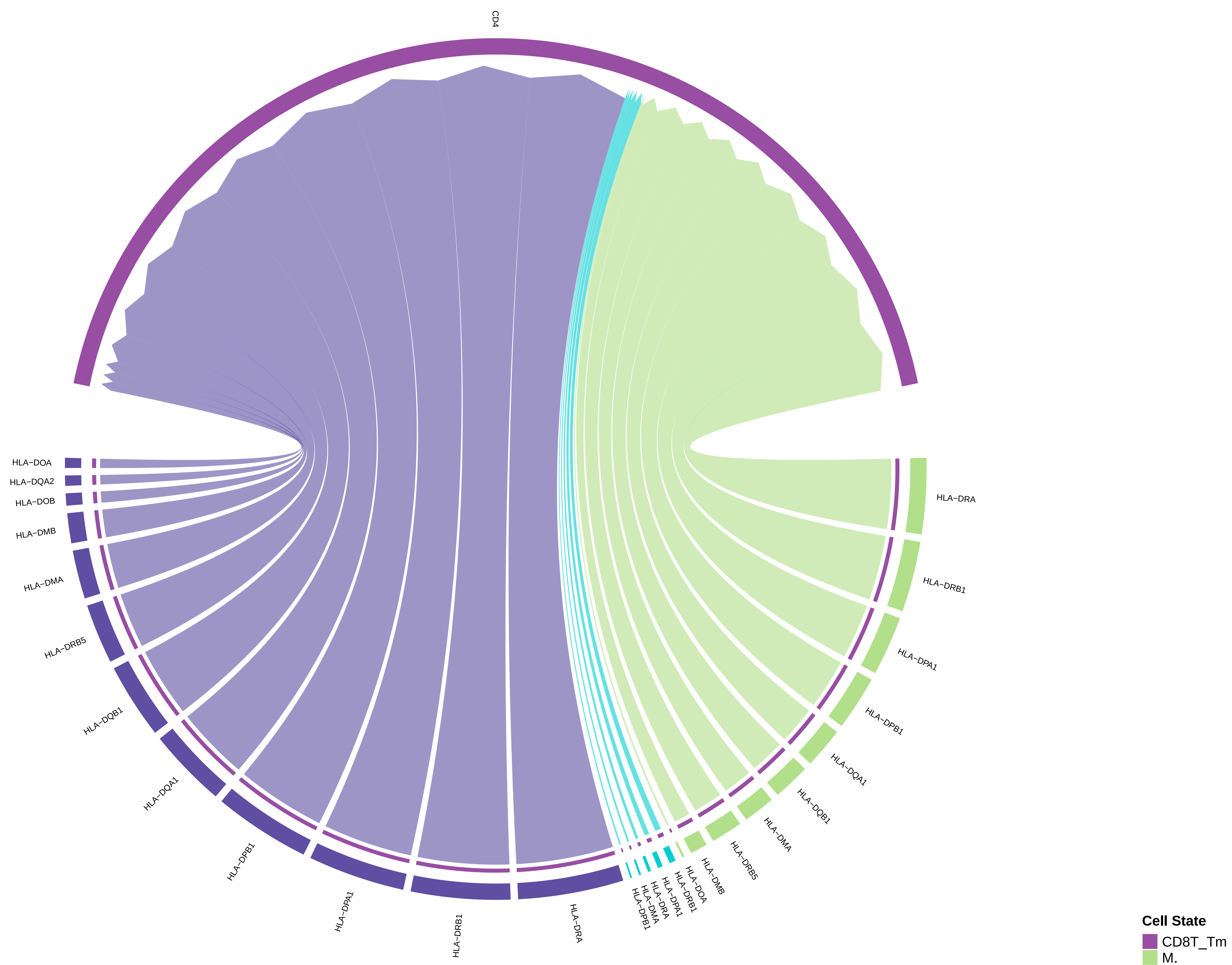

Cell Communication Analysis: Myeloid to Regulatory_T

Pathway: MIF

CCL5 High vs Low Expression Group Comparison

MIF signaling pathway network

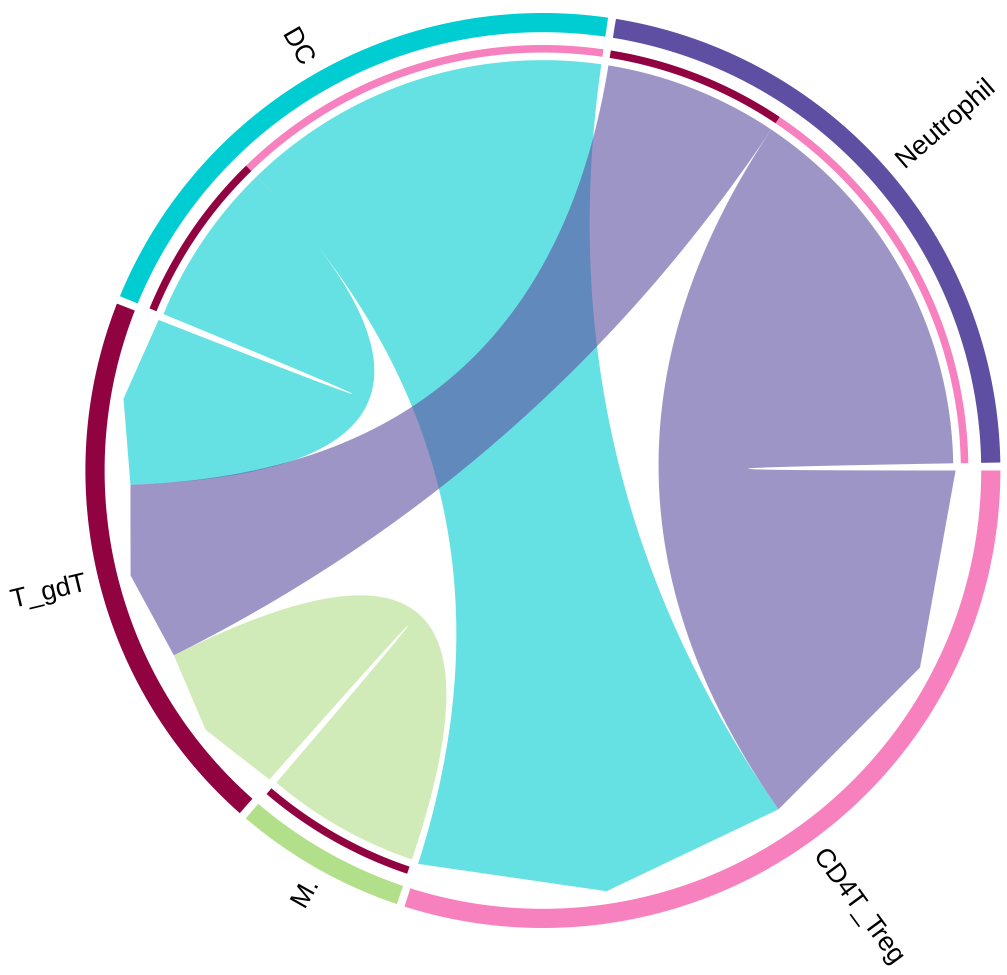

MIF signaling pathway network

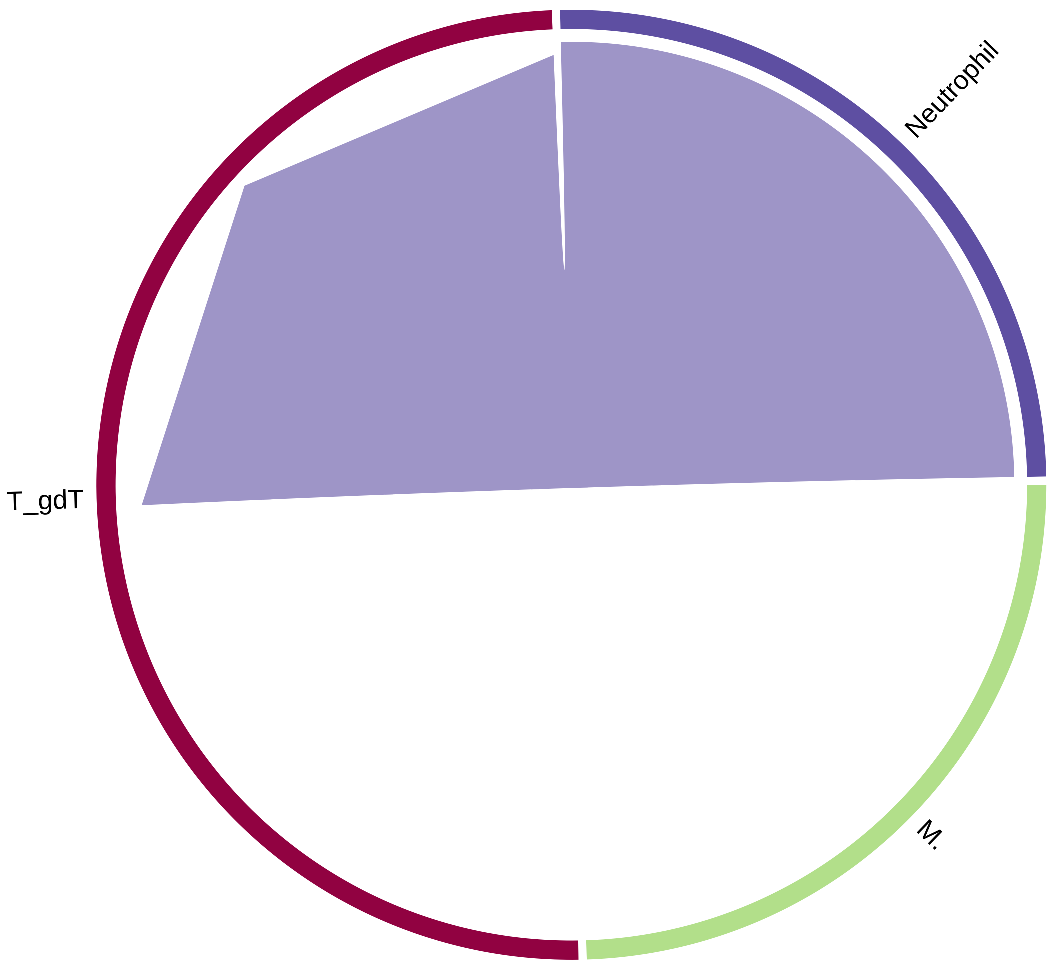

3

.9

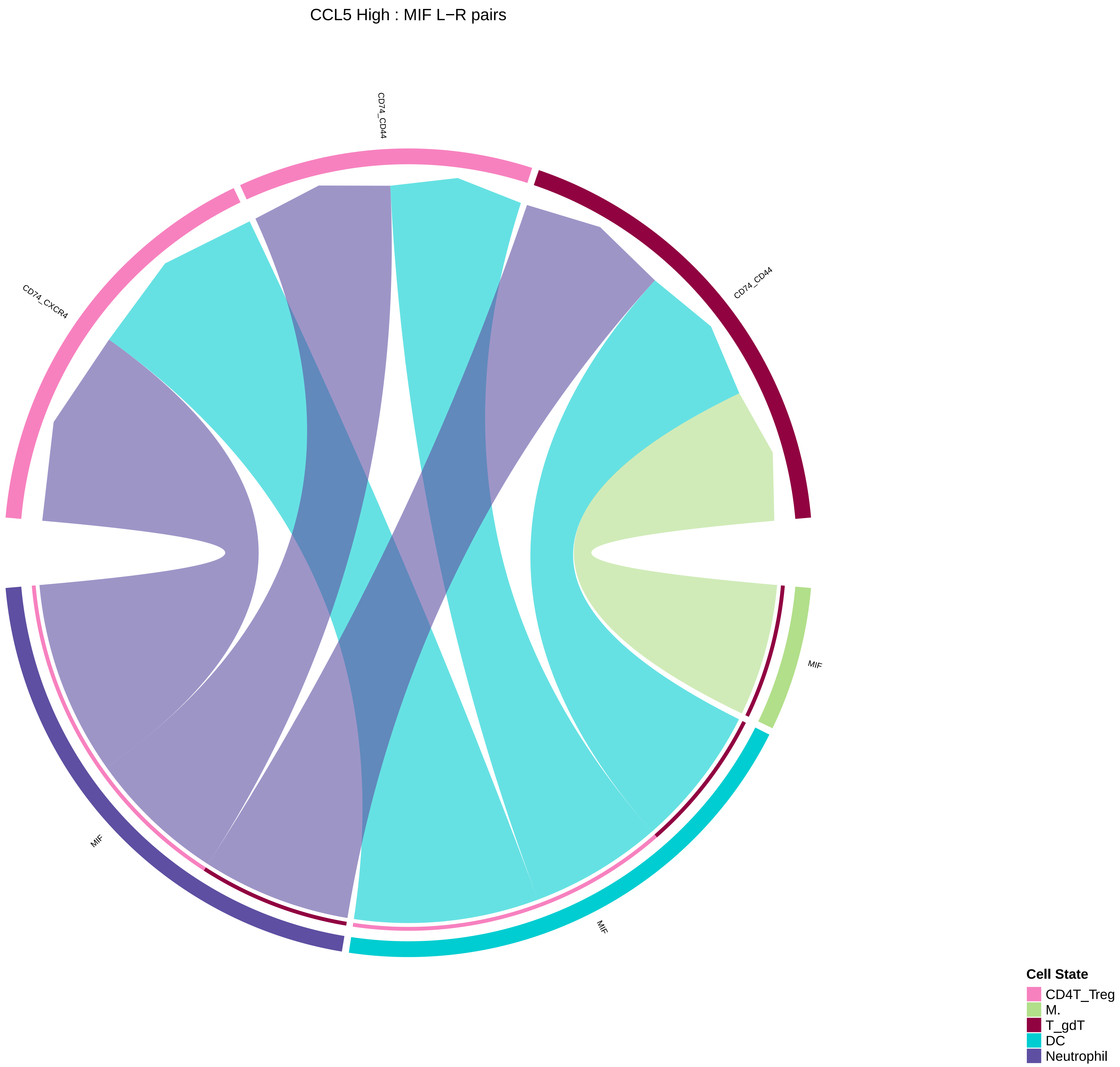
CCL5 High : MIF L−R pairs
CD74_CD44
CD74_CXCR4
CD74_CD44
MIF
MIF
MIF
Cell State
CD4T_Treg
M.
T_gdT
DC
Neutrophil

CCL5 Low : MIF L−R pairs

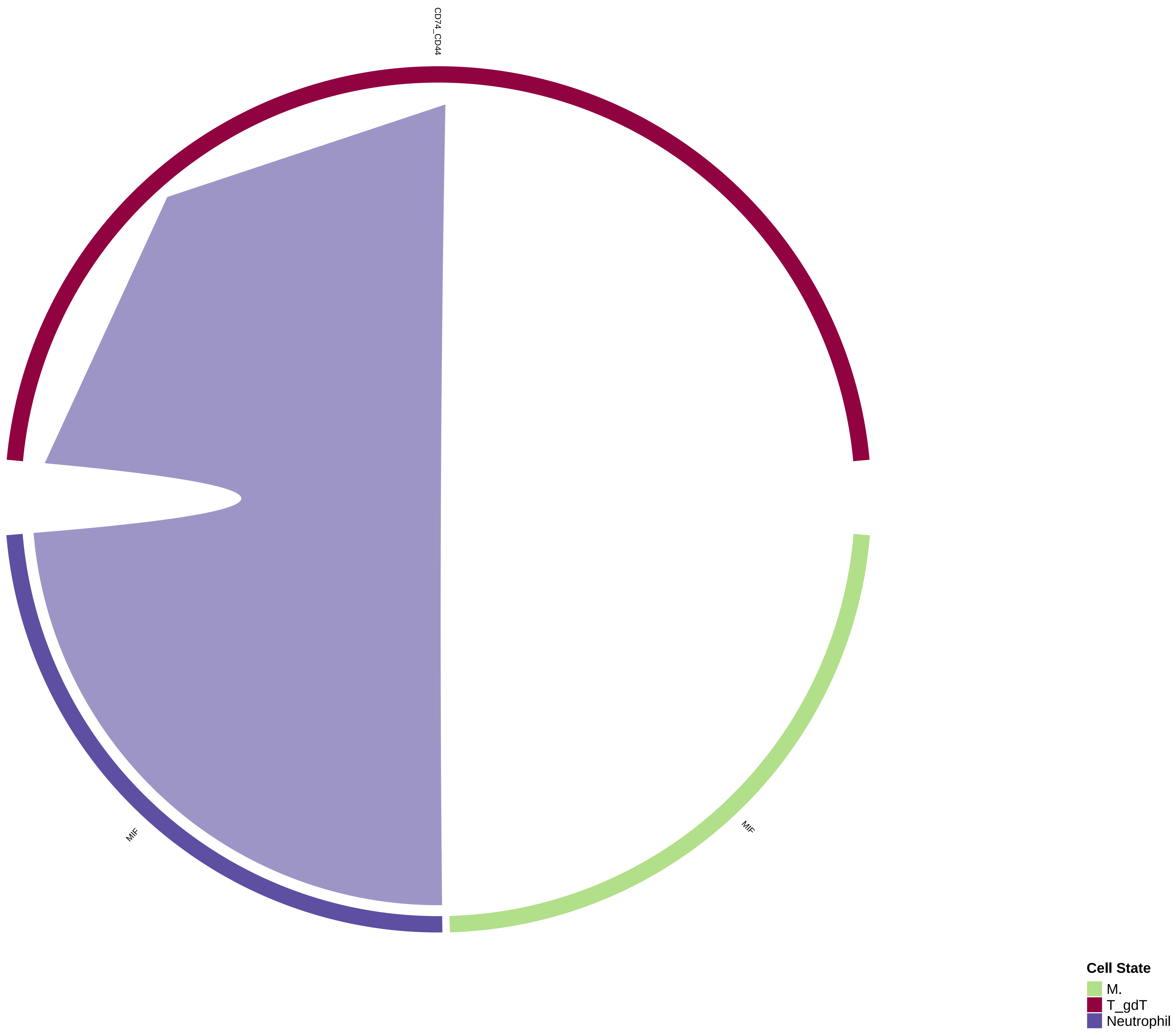

Cell Communication Analysis: Myeloid to Regulatory_T

Pathway: CLEC

CCL5 High vs Low Expression Group Comparison

CLEC signaling pathway network

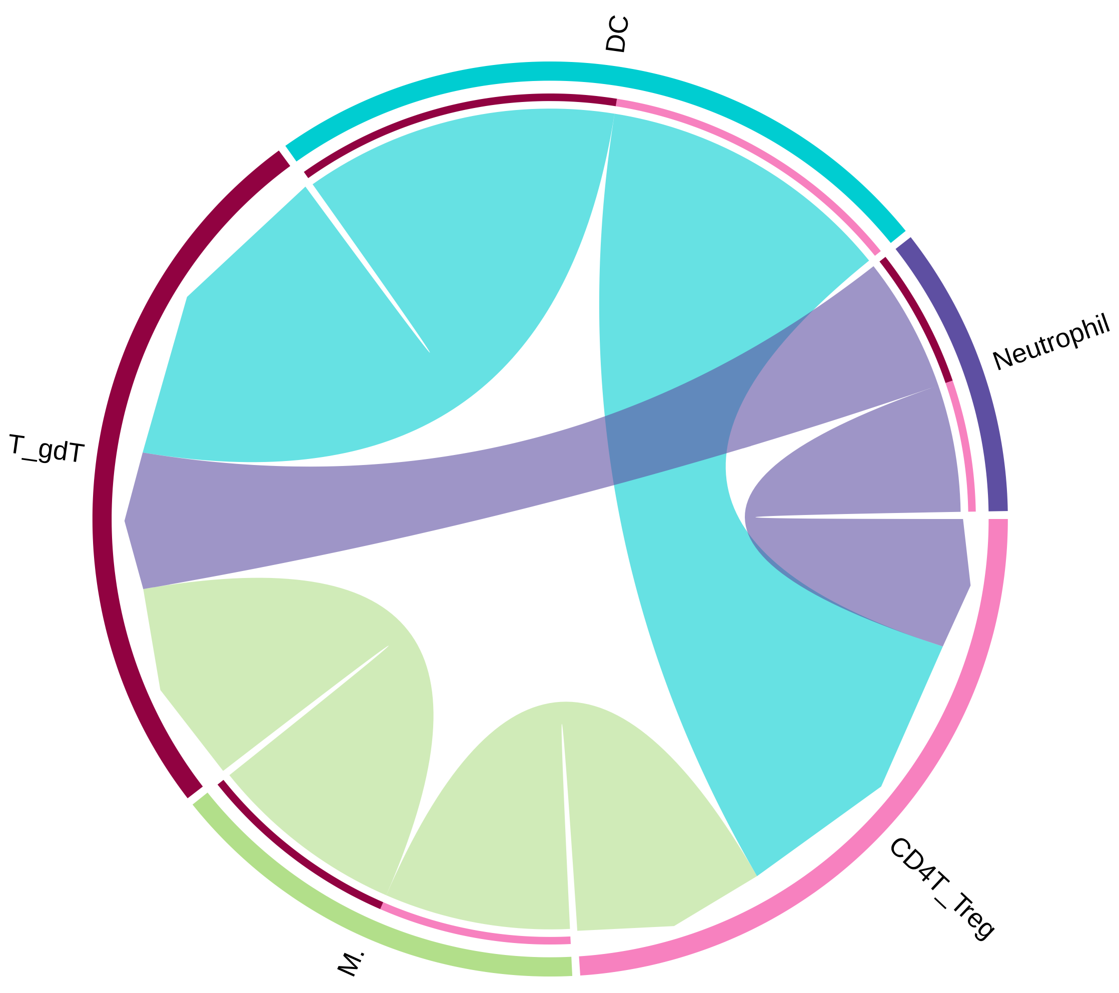

[illegible]1

CLEC signaling pathway network

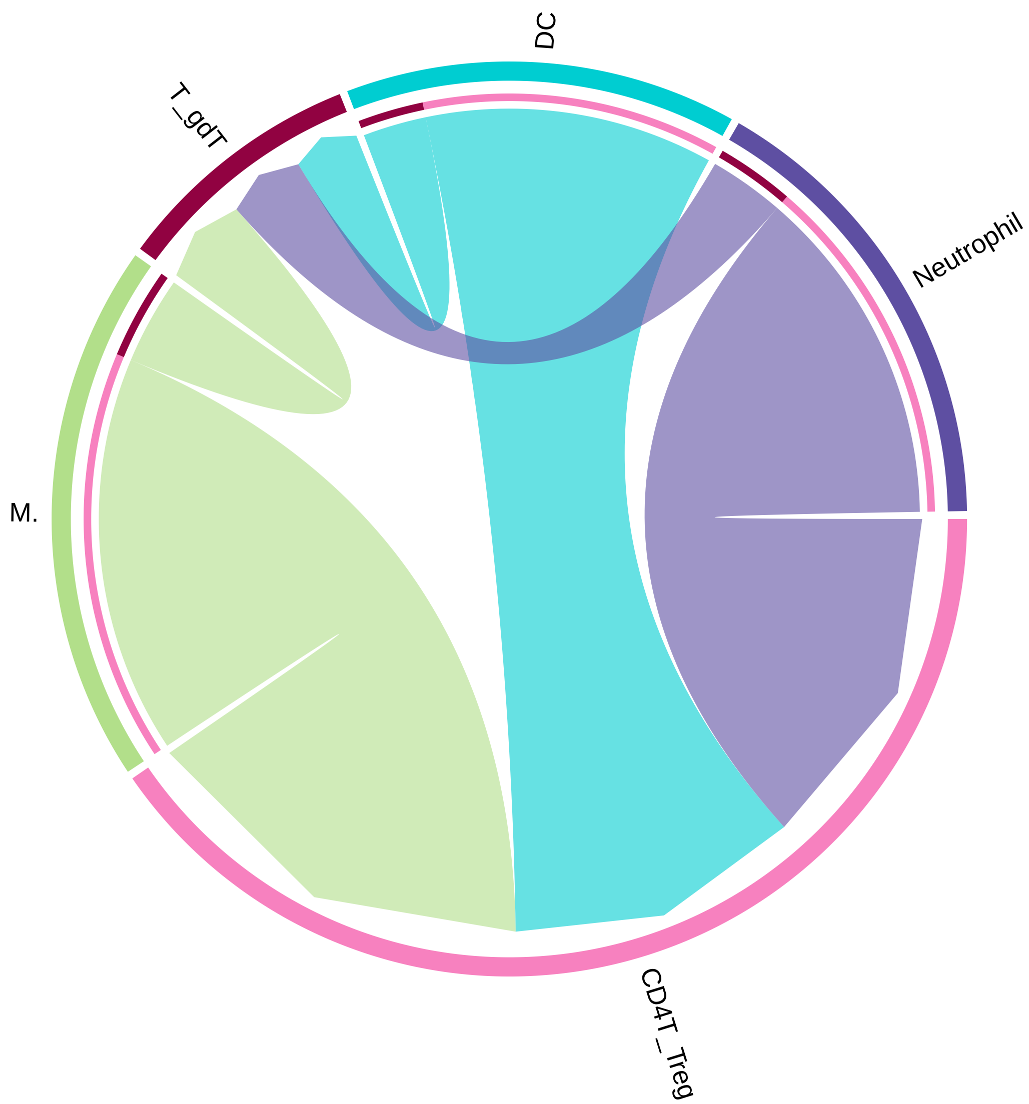

[illegible]1

CCL5 High : CLEC L−R pairs

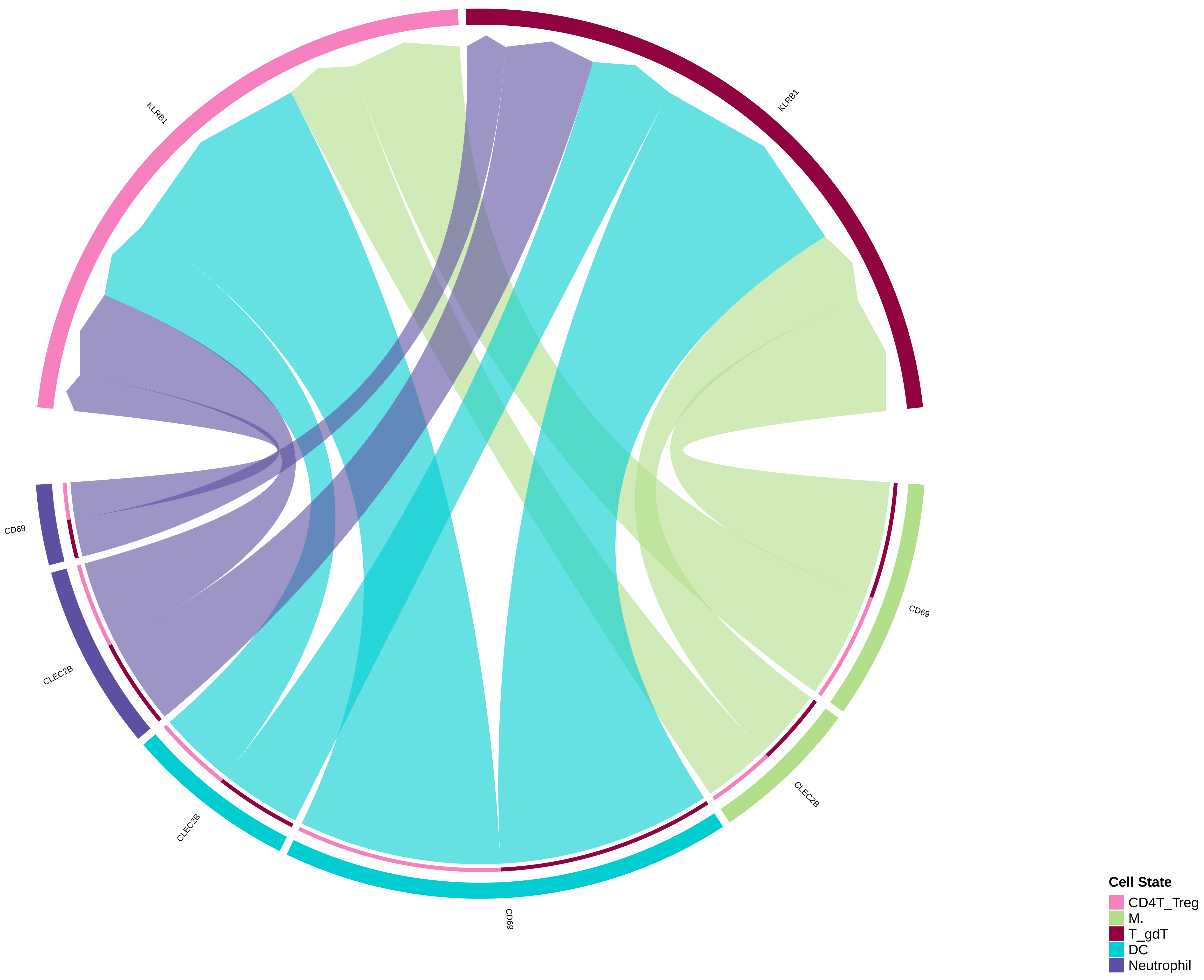

CCL5 Low : CLEC L−R pairs

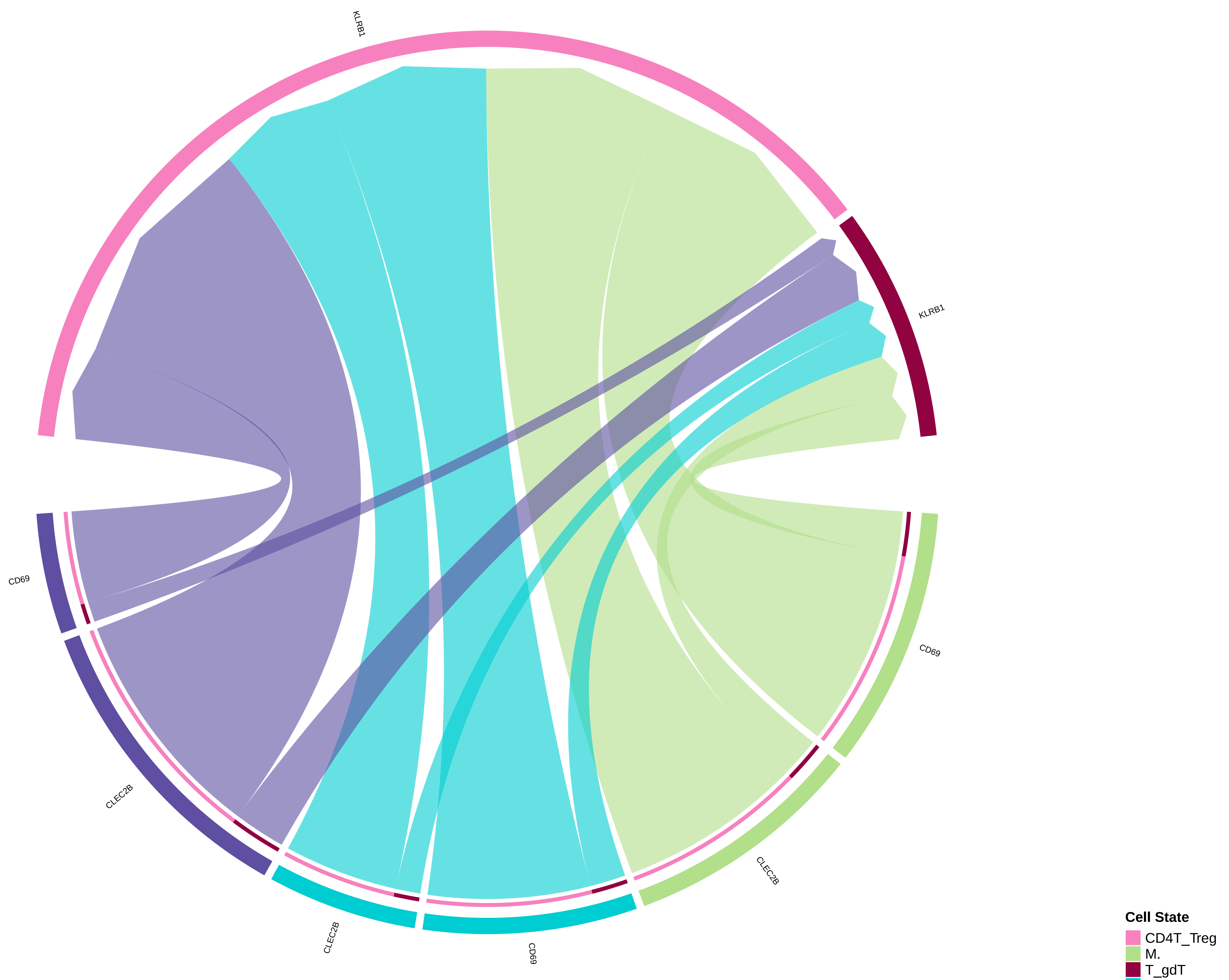

Cell Communication Analysis: Myeloid to Regulatory_T

Pathway: MIF

CCL5 High vs Low Expression Group Comparison

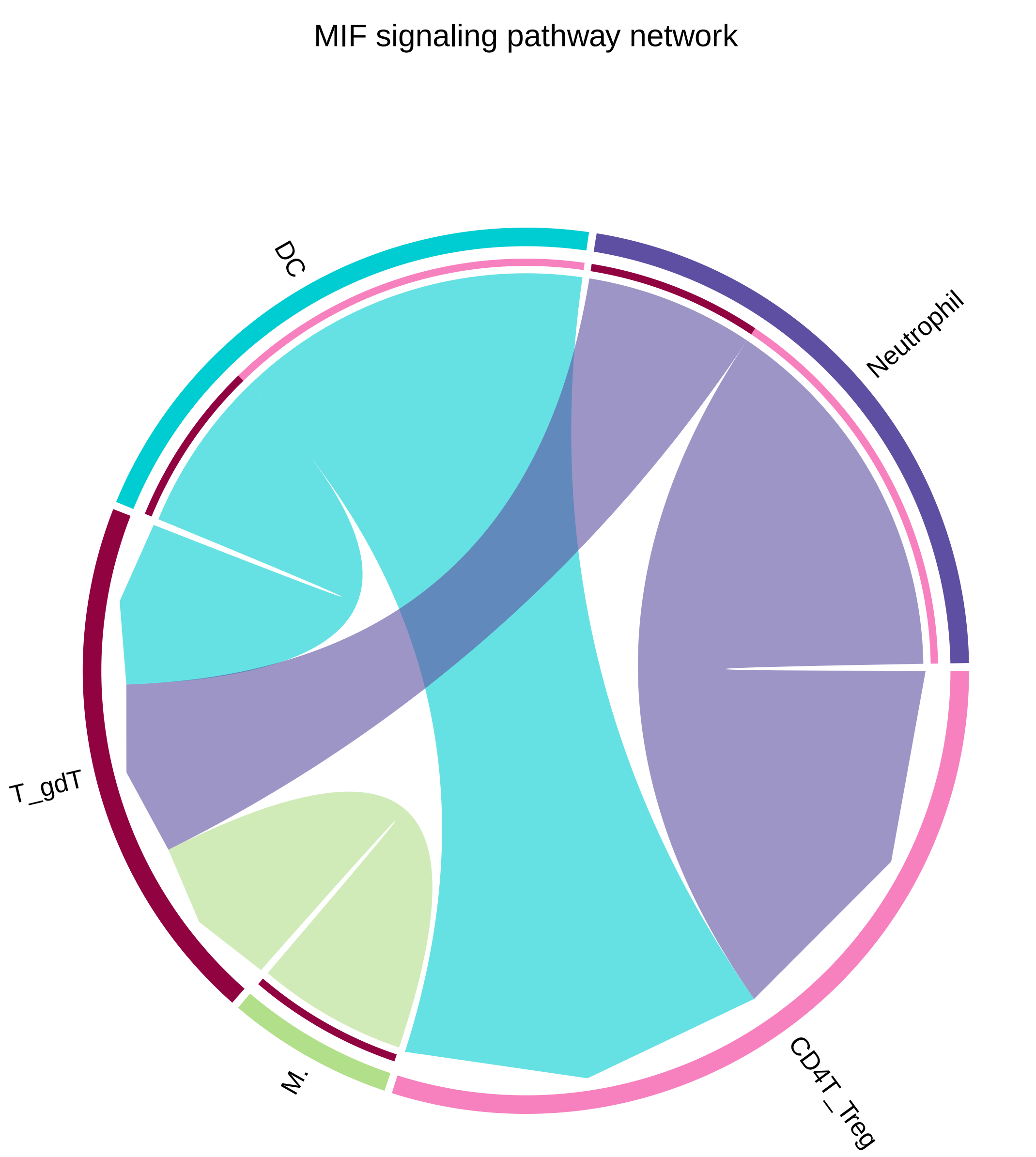

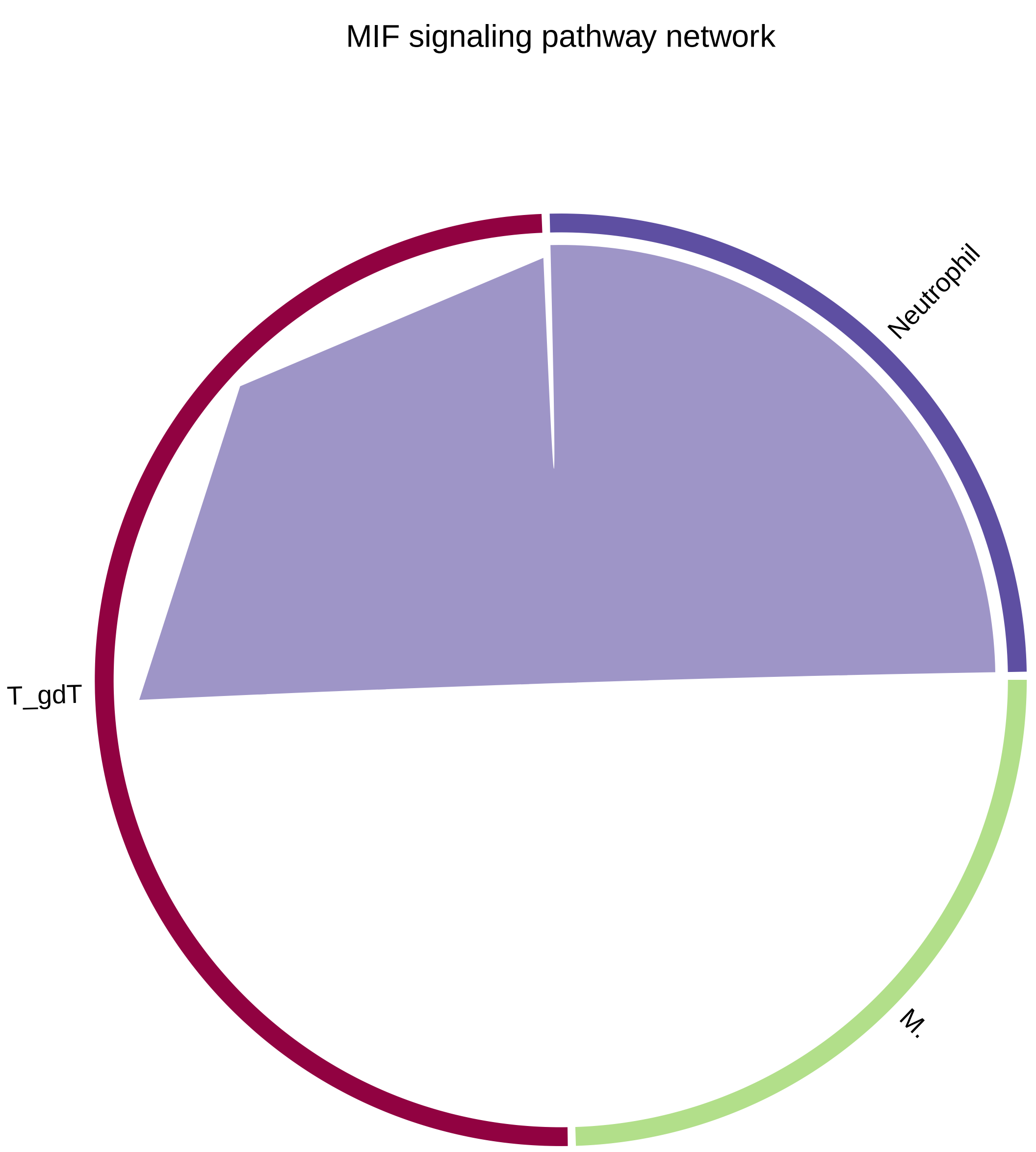

3

.9

CCL5 High : MIF L−R pairs

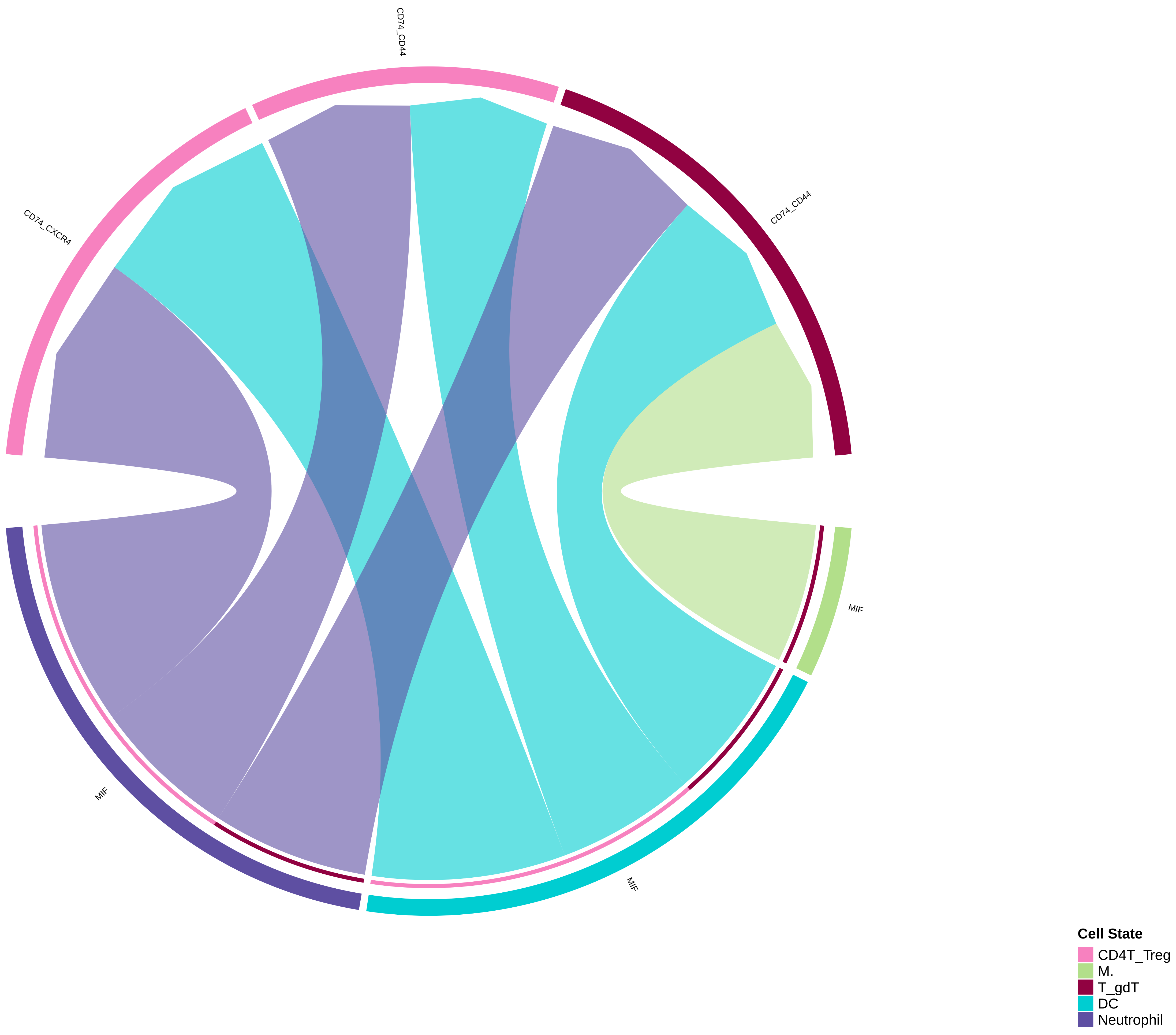

CCL5 Low : MIF L−R pairs

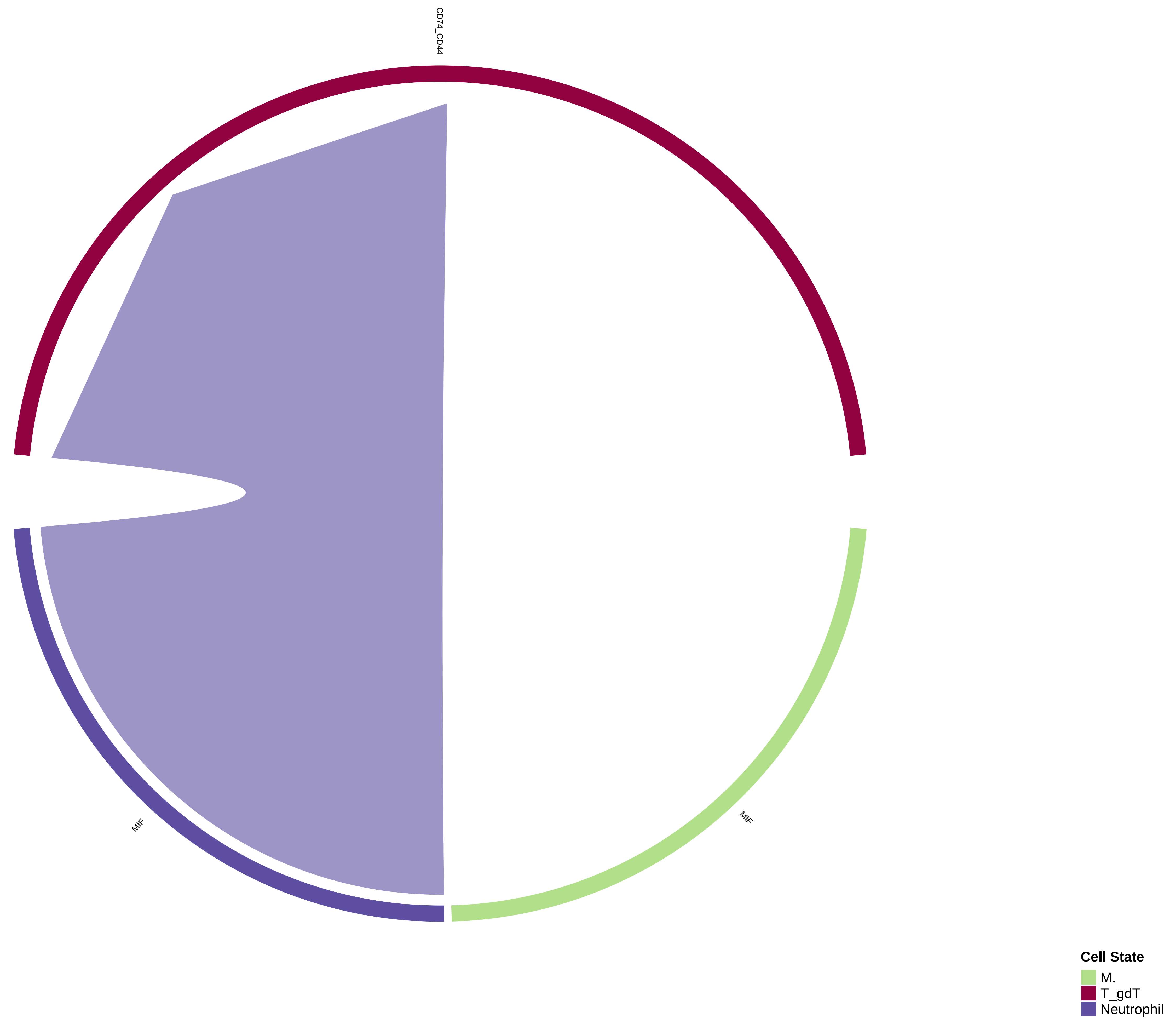

Cell Communication Analysis: Myeloid to Regulatory_T

Pathway: ADGRE5

CCL5 High vs Low Expression Group Comparison

ADGRE5 signaling pathway network

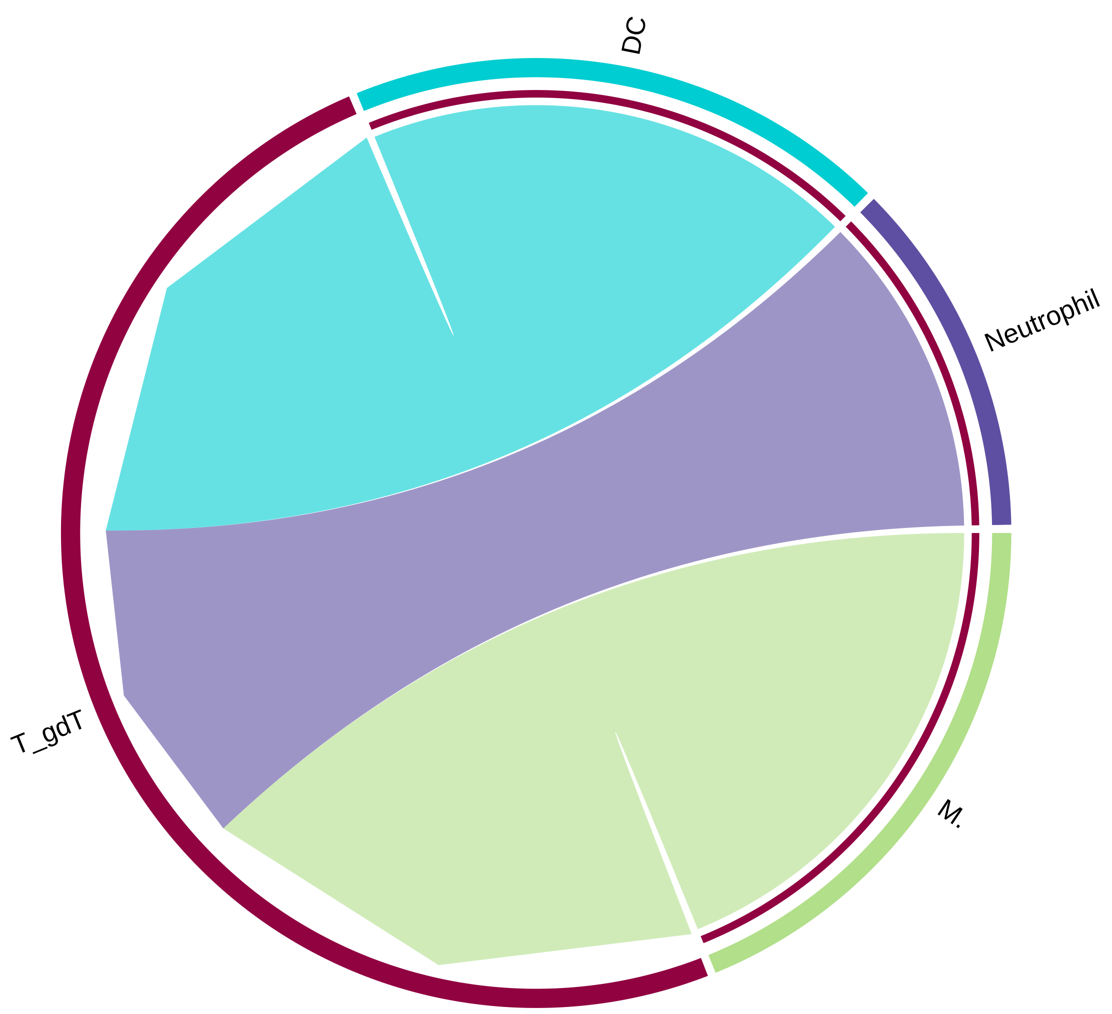

ADGRE5 signaling pathway network

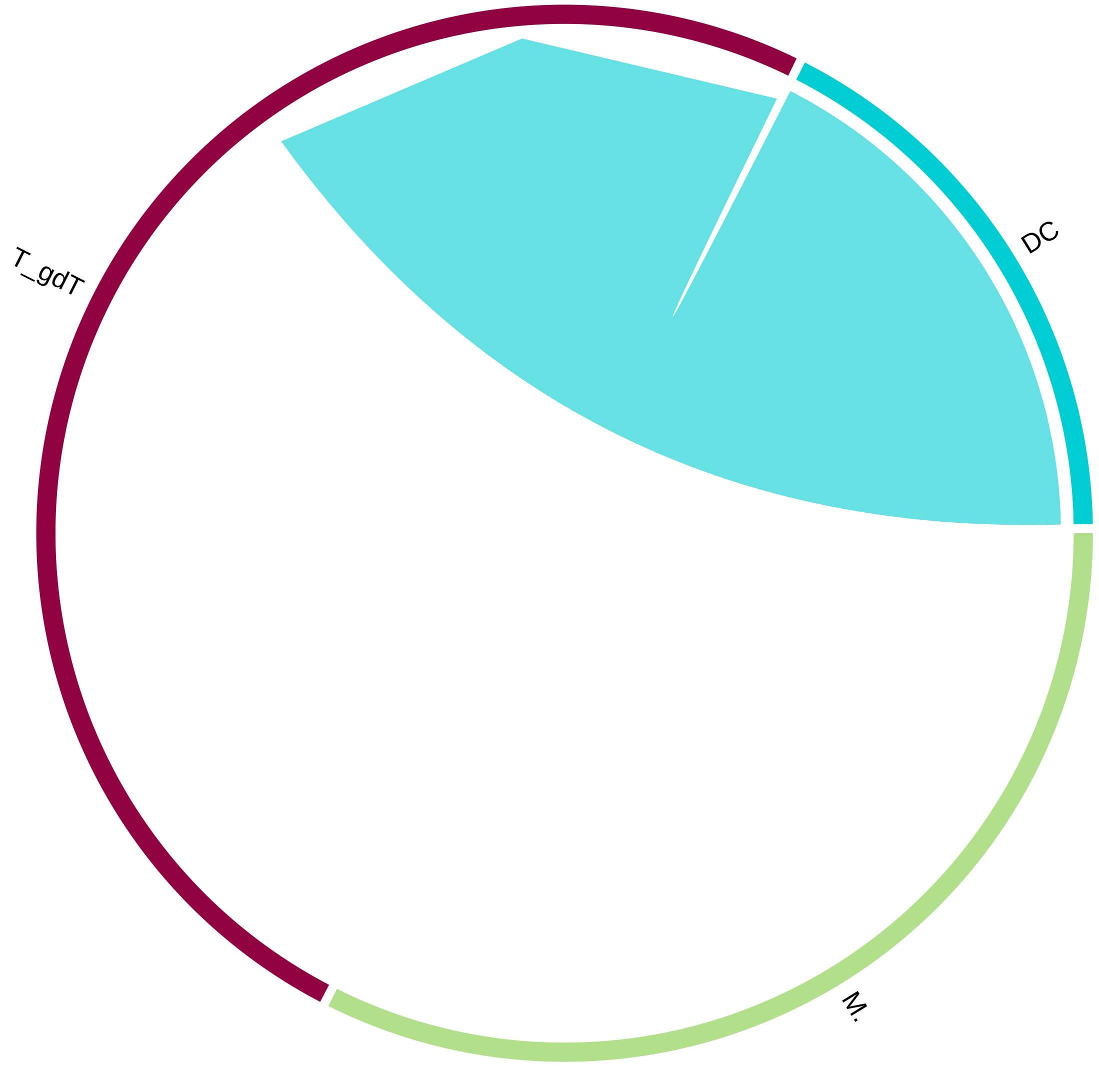

34

48

CCL5 High : ADGRE5 L−R pairs

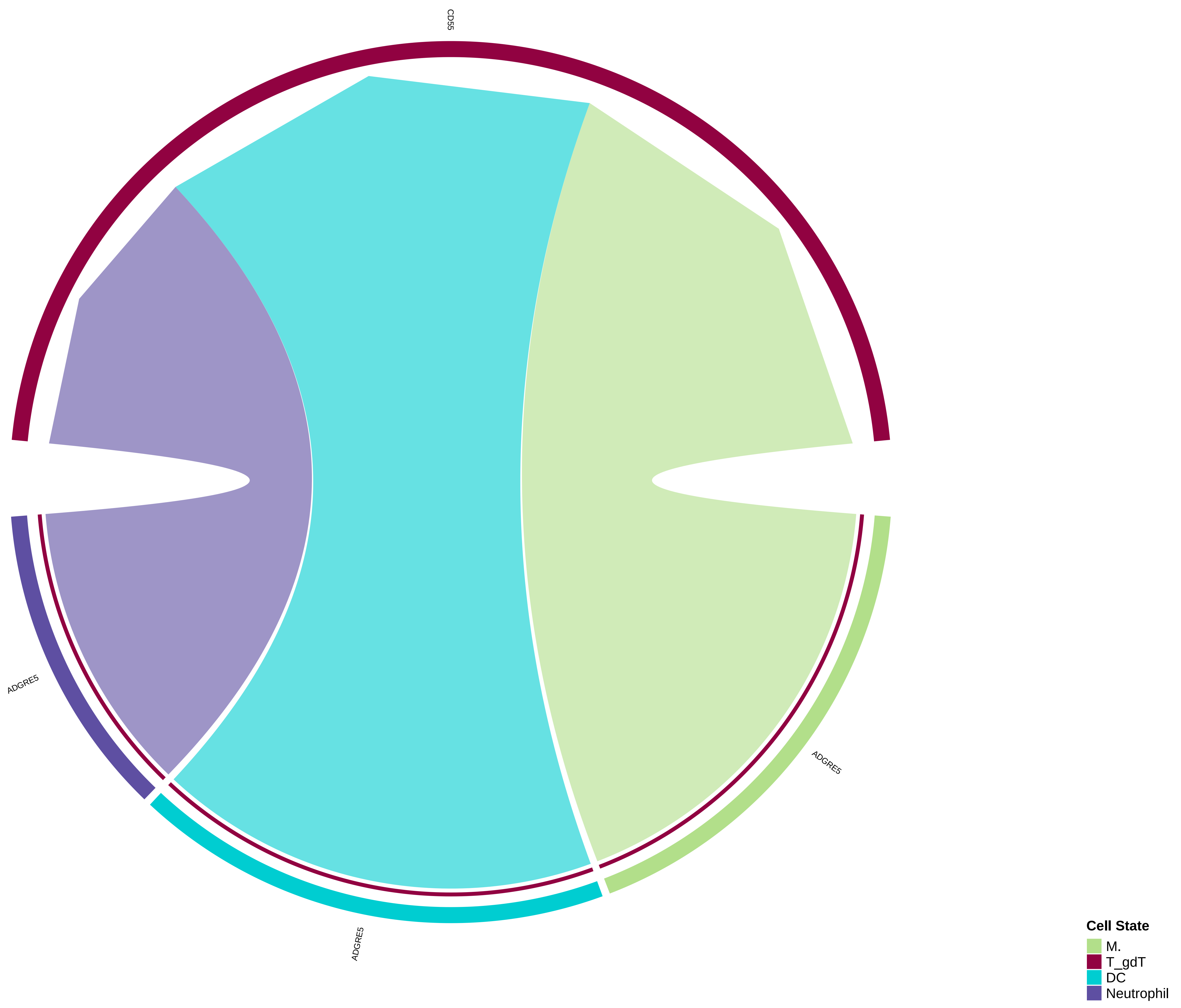

Comprehensive Ligand−Receptor Communication Analysis

CCL5 Low : ADGRE5 L−R pairs

CCL5 High−Upregulated Pathways (n = 5 pathways)

Bubble size = Communication intensity | Color = Expression level

Pathways analyzed: 5

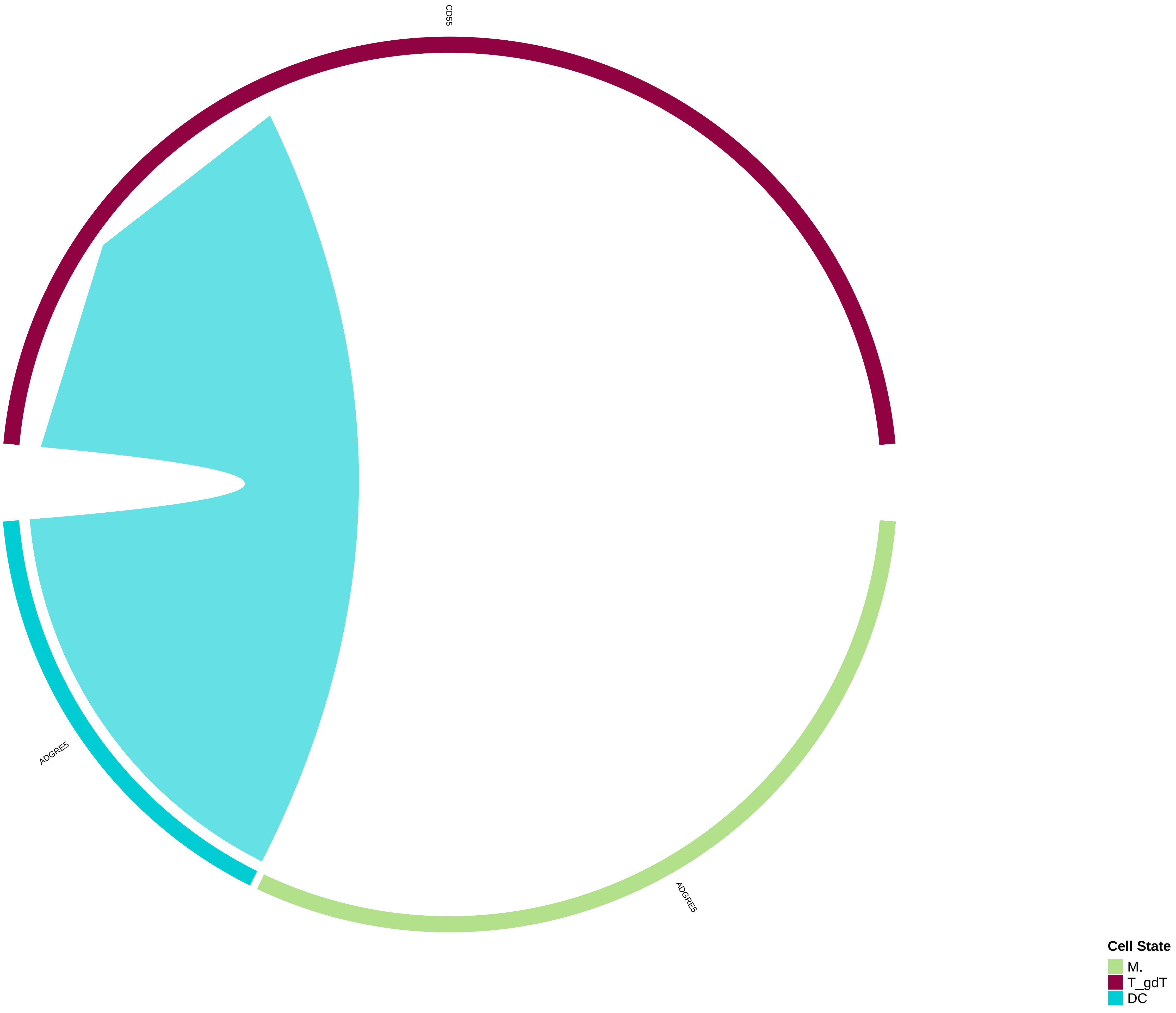

Supplement: Supplementary file 1 [file cancers-18-01271-s001.zip › cancers-4207408-supplementary/cancers-4207408-supplementary/S4_Cellchat_supplementary_materials.pdf]
