# Supplementary material for: CCL5 Orchestrates Paradoxical Immune Landscapes in NSCLC: Simultaneous Recruitment of Effector and Suppressor Cells Shapes Immunotherapy Resistance
Source: Cancers (Basel). 2026 Apr 16;18(8):1271. doi: 10.3390/cancers18081271 (PMC13114919; doi:10.3390/cancers18081271)
Supplement: Supplementary file 1 [file cancers-18-01271-s001.zip › cancers-4207408-supplementary/cancers-4207408-supplementary/Supplementary Figures.pdf]

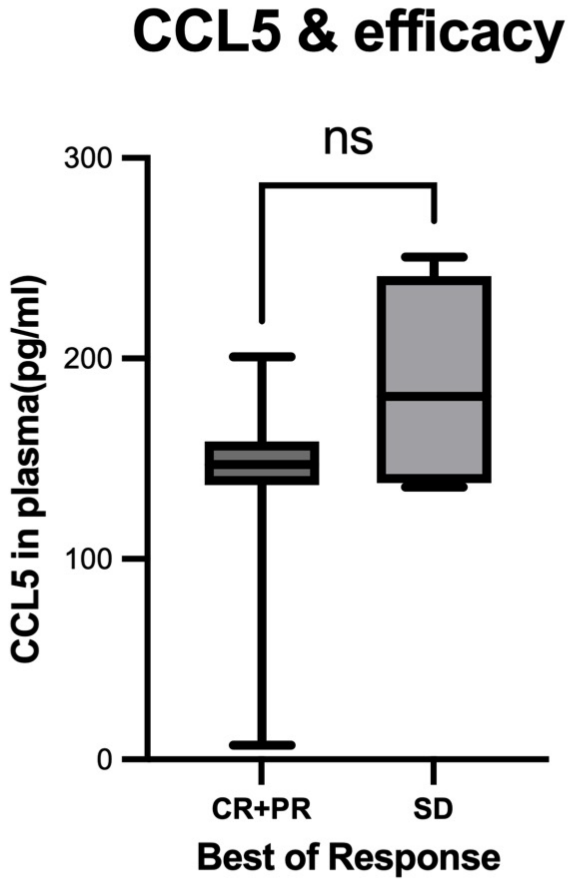

Figure S1. Comparison of plasma CCL5 levels between patients with different responses. Box plots showing plasma CCL5 concentrations (pg/ml) in patients stratified by best of response. CR+PR (complete response & partial response, n=27) represents patients who achieved clinical benefit, while SD (stable disease, n=6) represents patients with stable disease. ns indicates no statistically significant difference between the two groups ($p > 0.05$).

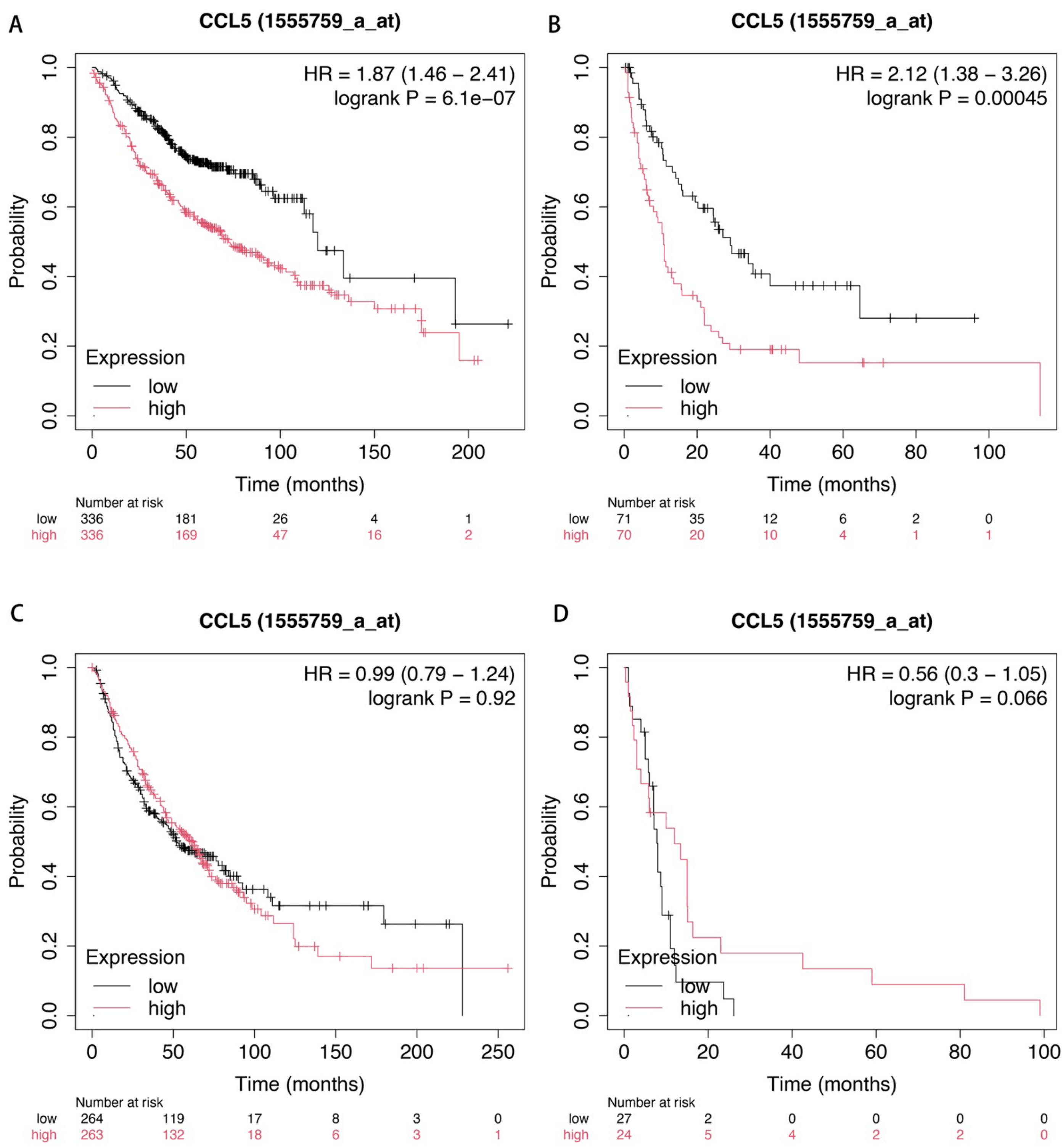

Figure S2. Association between CCL5 expression and survival outcomes in lung adenocarcinoma and lung squamous cell carcinoma. Kaplan-Meier survival curves showing the prognostic value of CCL5 (probe ID: 1555759_a_at) expression in lung cancer patients. A. Overall survival (OS) in lung adenocarcinoma (LUAD) patients stratified by CCL5 expression. High CCL5 expression (red line, n=336) was associated with significantly worse OS compared to low expression (black line, n=336), with a hazard ratio (HR) of 1.87 (95% CI: 1.46-2.41, logrank P = 6.1e-07). B. Progression-free survival (PFS) in LUAD patients. High CCL5 expression (red line, n=70) correlated with poorer PFS compared to low expression (black line, n=71), HR = 2.12 (95% CI: 1.38-3.26, logrank P = 0.00045). C. OS in lung squamous cell carcinoma (LUSC) patients. No significant difference was observed between high (red line) and low (black line) CCL5 expression groups, HR = 0.99 (95% CI: 0.79-1.24, logrank P = 0.92). D. PFS in LUSC patients. High CCL5 expression showed a trend toward better PFS with HR = 0.56 (95% CI: 0.3-1.05, logrank P = 0.066), though not reaching statistical significance. The numbers at risk at different time points are displayed below each curve.

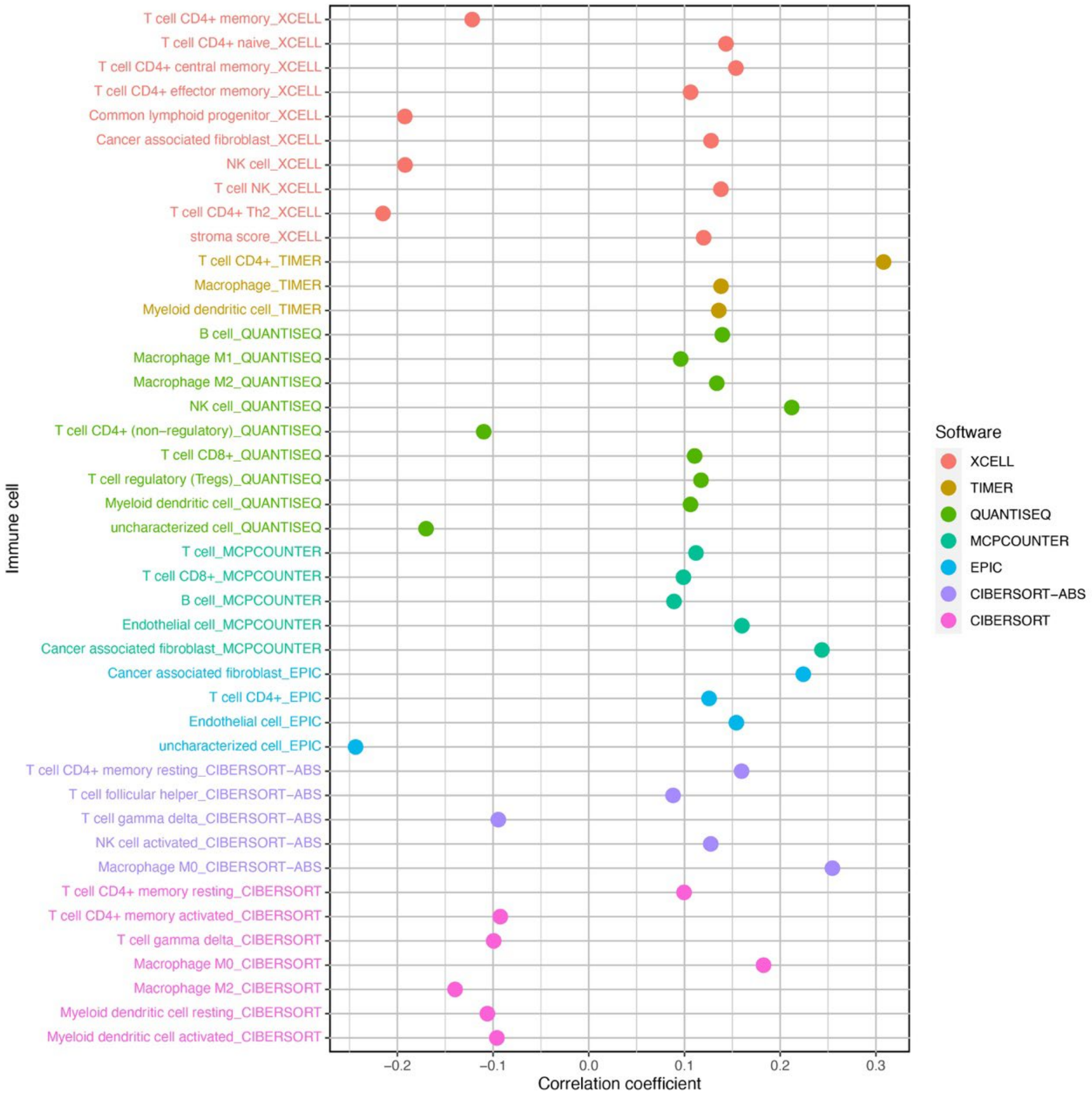

Figure S3. CCL5 orchestrates dual immune cell infiltration patterns in NSCLC. Spearman correlation analysis between CCL5 expression and immune cell infiltration scores across seven algorithms (XCELL, TIMER, QUANTISEQ, MCPCOUNTER, EPIC, CIBERSORT-ABS, CIBERSORT). CCL5-high tumors demonstrated paradoxical immune landscapes with significantly elevated infiltration of both immune effector populations ($CD8^+$ T cells, NK cells, activated dendritic cells, $CD4^+$ effector memory T cells) and immunosuppressive cells (regulatory T cells, myeloid-derived suppressor cells, macrophages). Dot colors represent different algorithms; x-axis shows correlation coefficients.

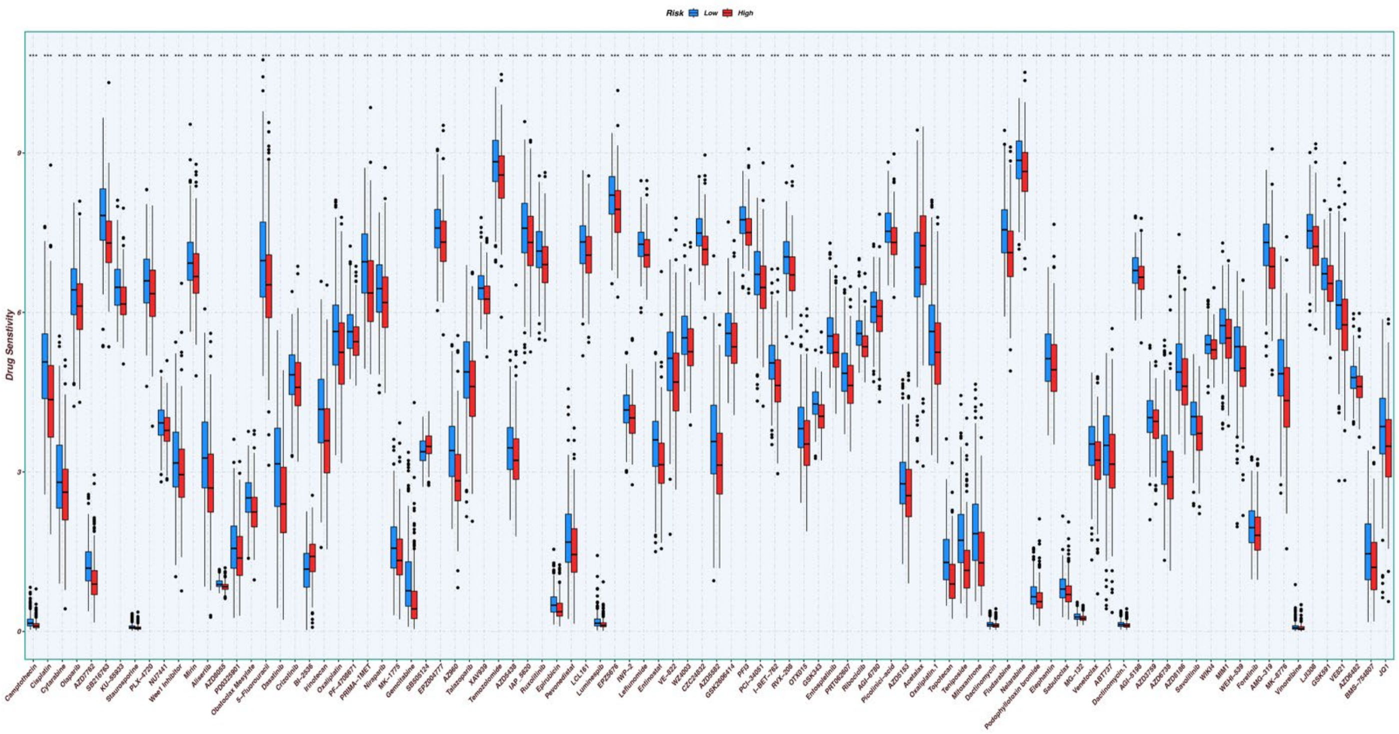

Figure S4. Association between CCL5 expression and drug sensitivity across multiple anti-cancer therapeutic agents. Box plots illustrating the correlation between CCL5 gene expression levels and drug sensitivity for a comprehensive panel of anti-cancer drugs. Each box plot represents a different therapeutic agent, with drugs labeled on the x-axis. The y-axis shows drug sensitivity scores, where higher values indicate greater sensitivity and lower values indicate higher resistance. Blue boxes represent samples with low CCL5 expression, while red boxes represent samples with high CCL5 expression. * P < 0.05, ** P < 0.01, *** P < 0.001, and "ns" indicates no significant difference.

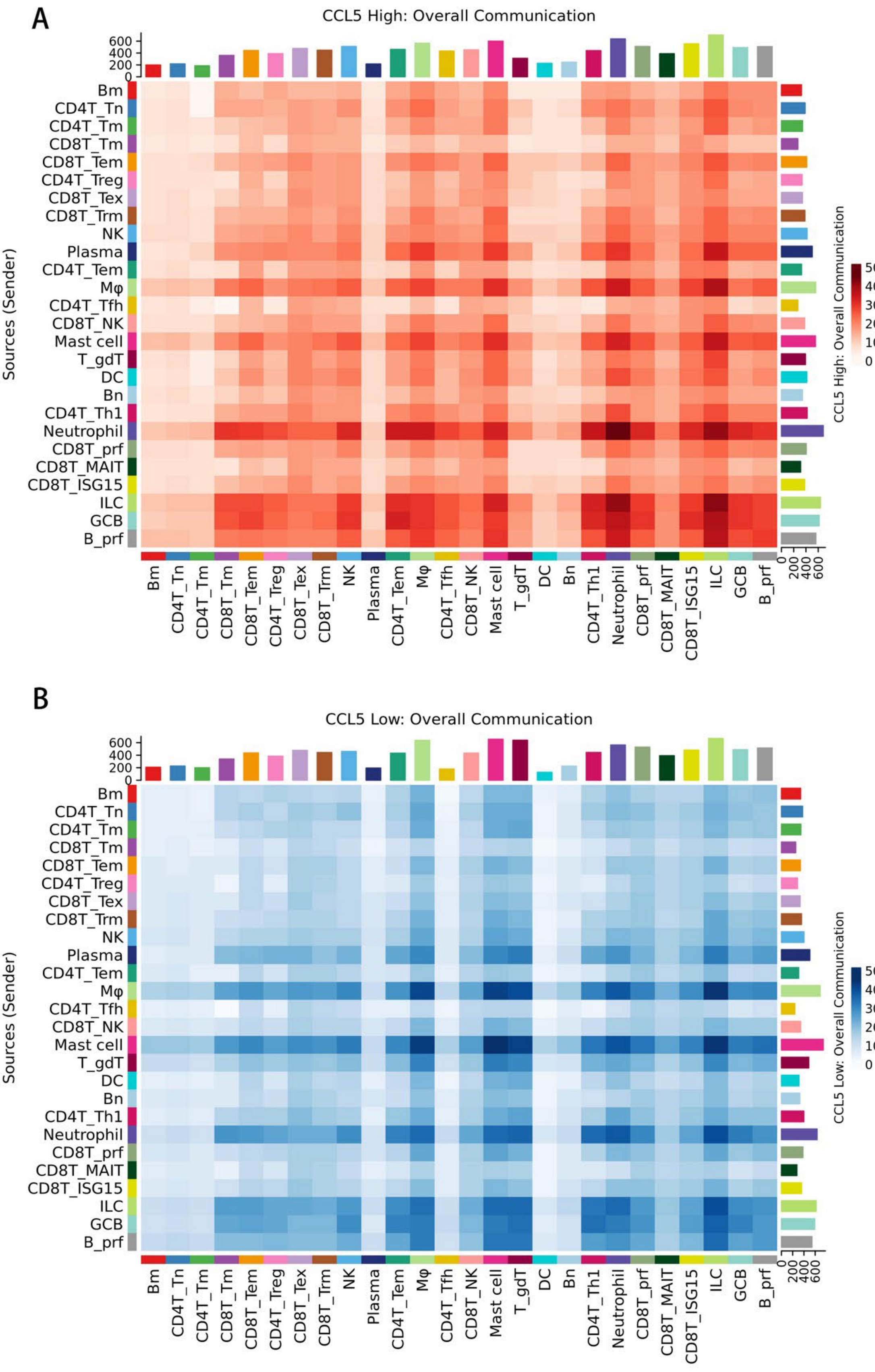

Figure S5. Differential cell–cell communication patterns between CCL5 high and low expression groups revealed with CellChat analysis. Heatmaps illustrating the overall communication probability and strength between different cell types in tumor microenvironment stratified by CCL5 expression levels. A. CCL5 high expression group showing overall communication patterns. The heatmap displays sender cells (rows) and receiver cells (columns), with color intensity representing the communication strength. B. CCL5 low expression group showing overall communication patterns with the same layout and color scheme.

Macrophage (Mφ) to T cell communication: In the CCL5 high group, macrophages show enhanced signaling to multiple T cell subsets including $CD4^+$ T cells (Tn, Tm, Tem, Treg, Tfh) and $CD8^+$ T cells (Tm, Tem, Tex, Trm, NK), with stronger red intensity compared to the weaker blue signals in the CCL5 low group.

Neutrophil-T cell axis: Neutrophils exhibit more robust communication with CD8+ T cell subsets (particularly CD8T_prf and CD8T_MAIT) in the CCL5 high group, as evidenced by darker red coloring versus lighter blue in the CCL5 low group.

Dendritic cell (DC) interactions: DC-mediated signaling to various T cell populations shows enhanced activity in the CCL5 high expression context, suggesting improved antigen presentation and T cell priming.

Bidirectional communication: T cells (both $CD4^+$ and $CD8^+$ subsets) also show increased outgoing signals to myeloid cells (Mφ, DC, Neutrophil) in the CCL5 high group, indicating enhanced reciprocal crosstalk that may promote anti-tumor immunity.

Cell type abbreviations: Bm (B memory), CD4T_Tn ($CD4^+$ T naive), CD4T_Tm ($CD4^+$ T memory), CD8T_Tm ($CD8^+$ T memory), CD8T_Tem ($CD8^+$ T effector memory), CD4T_Treg ($CD4^+$ T regulatory), CD8T_Tex ($CD8^+$ T exhausted), CD8T_Trm ($CD8^+$ T resident memory), NK (natural killer), Mφ (macrophage), CD4T_Tem ($CD4^+$ effector memory), CD4T_Tfh ($CD4^+$ T follicular helper), CD8T_NK ($CD8^+$ NK-like T), Mast cell, T_gdT (gamma-delta T), DC (dendritic cell), Bn (B naive), CD4T_Th1 ($CD4^+$ Th1), Neutrophil, CD8T_prf ($CD8^+$ proliferating), CD8T_MAIT (mucosal-associated invariant T), CD8T_ISG15 ($ISG15^+$ $CD8^+$ T), ILC (innate lymphoid cell), GCB (germinal center B), B_prf (B proliferating).

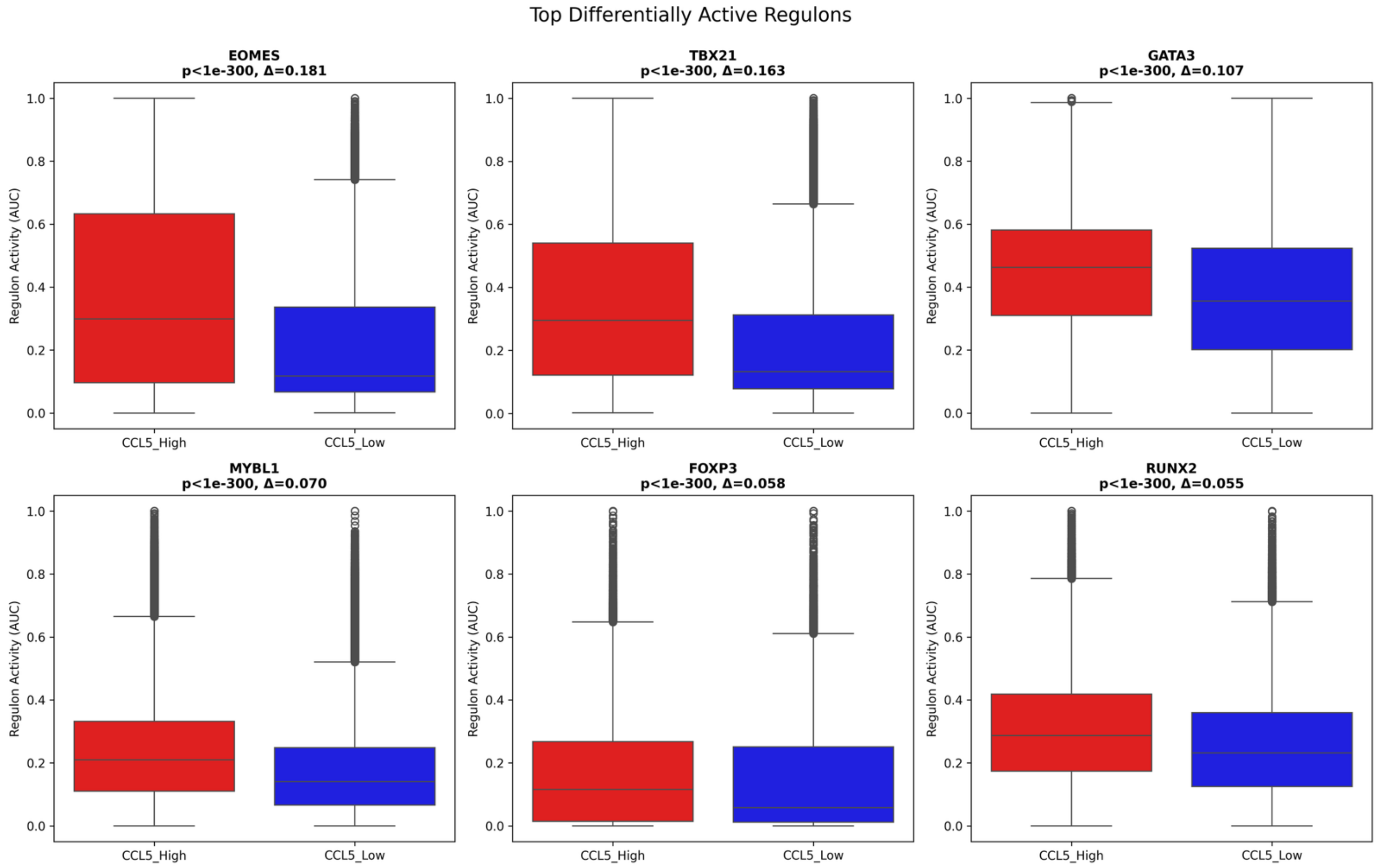

Figure S6. Comparison of regulon activity between high and low CCL5 expression groups in top six differentially active regulons. Box plots showing the regulon activity (measured as Activity Units, AUC) for six transcription factor regulons that exhibit significant differential activity between CCL5-high (red) and CCL5-low (blue) groups. The delta (Δ) values represent the effect size of differential activity between groups.

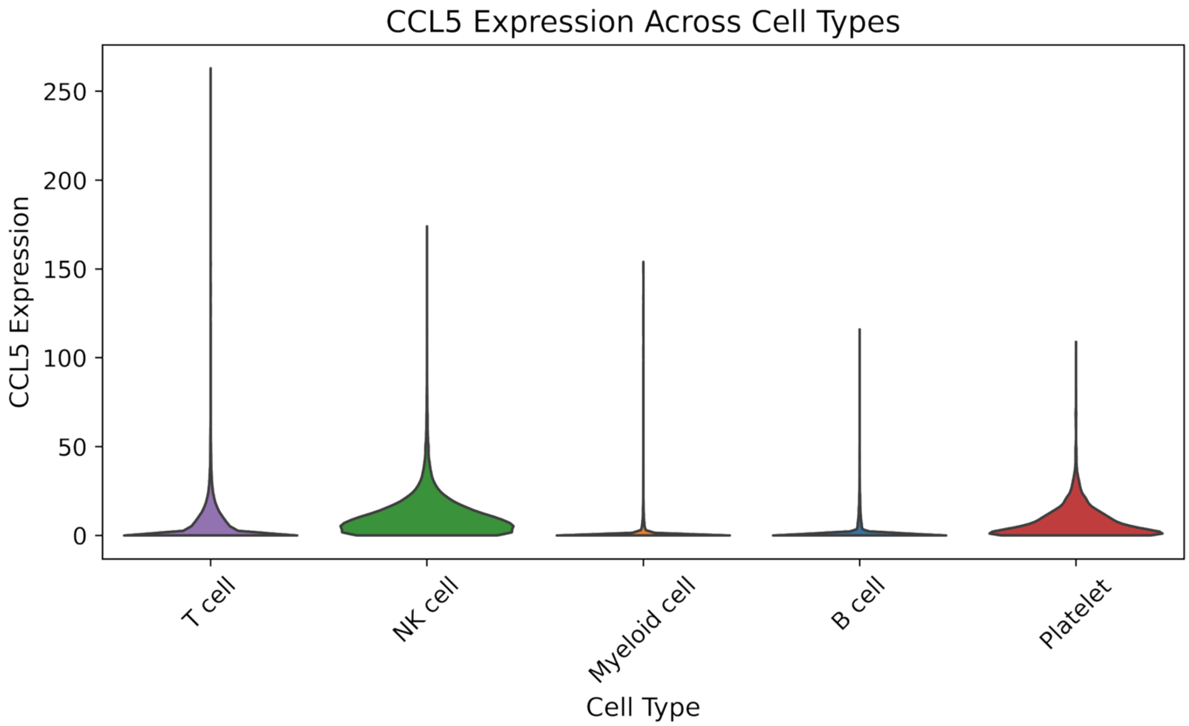

Figure S7. Violin plot showing CCL5 mRNA expression across major immune cell types in PBMCs. scRNA-seq dataset was re-analyzed from previously published dataset [29]. CCL5 is predominantly expressed in T cells and NK cells.
